# Investigating Factors Influencing Disease Progression in Patients With Non-Alcoholic Fatty Liver Disease

**DOI:** 10.14740/jocmr6424

**Published:** 2026-02-28

**Authors:** Yi-Chieh Tseng, Rewadee Jenraumjit, Ming-Jong Bair, Chung-Yu Chen, Fu-Shih Chen

**Affiliations:** a Department of Pharmacy, Taitung Mackay Hospital, Taitung, Taiwan, Republic of China; b Master Program in Clinical Pharmacy, School of Pharmacy, Kaohsiung Medical University, Kaohsiung, Taiwan, Republic of China; c Department of Pharmaceutical Care, Faculty of Pharmacy, Chiang Mai University, Chiang Mai, Thailand; d Master of Science Program (Mental Health), Multidisciplinary and Interdisciplinary School, Chiang Mai University, Chiang Mai, Thailand; e Division of Gastroenterology, Department of Internal Medicine, Taitung Mackay Memorial Hospital, Taitung, Taiwan, Republic of China; f Graduate School of Pharmaceutical Sciences, Nihon Pharmaceutical University, Japan; g Faculty of Pharmaceutical Sciences, Nihon Pharmaceutical University, Japan

**Keywords:** Cluster analysis, Non-alcoholic fatty liver disease, Electronic health records, Unsupervised learning, Clinical phenotypes

## Abstract

**Background:**

With no approved pharmacological treatments for non-alcoholic fatty liver disease (NAFLD) in Taiwan, identifying protective and risk factors is crucial for preventing disease progression. Given the clinical heterogeneity of NAFLD, this study aimed to identify clinically meaningful NAFLD phenotypes using electronic medical records (EMRs) and unsupervised clustering, stratify risk across different clusters, identify factors associated with disease progression, and derive a parsimonious set of predictors for high-risk phenotypes.

**Methods:**

This study was a retrospective cohort study conducted in three steps with iterative model training. In step 1, patients diagnosed with NAFLD were identified, and all relevant patient data were extracted, followed by clustering analysis using the k-prototype algorithm. In step 2, survival analysis and Cox regression were applied to perform risk stratification across clusters. In step 3, Lasso regression, logistic regression, and receiver operating characteristic (ROC) curve analysis were used to identify potential protective and risk factors associated with NAFLD and to derive a parsimonious set of predictors for high-risk phenotypes across different risk strata.

**Results:**

Step 1: The analysis of 6,023 patients identified four distinct phenotypic clusters. The first cluster had the most severe disease, the second the least. Step 2: Among 4,998 patients, the first cluster faced the highest risk for all outcomes, with a median survival of 3.06 years, significantly different from the others. There was no significant risk difference between the second and third clusters. Step 3: A comparison of the highest-risk and lowest-risk clusters finally identified 17 potential variables.

**Conclusions:**

Using multiple analytical models, this study identified 17 potential risk factors associated with NAFLD progression. Their combined assessment may inform future risk stratification and hypothesis generation. Further validation is required before clinical application.

## Introduction

Non-alcoholic fatty liver disease (NAFLD) is one of the most prevalent liver disorders in clinical practice. If uncontrolled, NAFLD may progress to non-alcoholic steatohepatitis (NASH), liver fibrosis, cirrhosis, or liver cancer. Based on the meta-analysis of epidemiological data, the overall prevalence of NAFLD in Taiwan is approximately 7.9% to 63.8% [1, 2]. Research predicted that by 2020 to 2030, patients with liver cirrhosis due to NAFLD would become the leading indication for liver transplants (LT) [3, 4]. In Taiwan, the NAFLD population has a significantly higher incidence of liver cancer compared to the general population (adjusted hazard ratio (aHR) 6.08; 95% confidence interval (CI) 4.73–7.48).

Given the lack of approved treatments for NAFLD in Taiwan and many unknown aspects of the disease [5–7], exploring additional protective or risk factors is crucial for improving clinical care. Recent studies have leveraged unsupervised machine learning, a popular method that allows clustering of large, unlabeled datasets to identify patient characteristics within different clusters [8]. This approach can uncover unknown protective or risk factors more effectively. Unsupervised machine learning has been successfully applied in medicine, such as in a prospective cohort study that used k-means clustering and generalized low-rank modeling to identify four coronary artery disease (CAD) clusters with distinct features, linking them to clinical outcomes [9]. The study further investigated the relationship between clusters and clinical outcomes. Another study employed k-prototype clustering to analyze healthcare data, identifying diagnostic characteristics, variability in doctors’ specialties, and medication usage patterns. This approach identified potential factors in each cluster, greatly aiding future medical applications [10]. Compared with traditional statistical methods, the study results revealed that machine learning techniques might be used to discover an integrated risk assessment for the distinctive subgroups within a complex population.

The study aimed to identify clinically meaningful NAFLD phenotypes using electronic medical records (EMRs) and unsupervised clustering, stratify risk across different clusters, identify potential factors associated with disease progression, and derive a parsimonious set of predictors for high-risk phenotypes progressing to severe outcomes, including liver fibrosis, liver cirrhosis, hepatocellular carcinoma (HCC), or mortality. These findings may provide valuable insights for clinicians to slow NAFLD progression without established treatments. To address the presumption and achieve the study aim above, the objective was to build multiple models to explore the factors of interest (including protective or risk factors). We also tested different models for the research and conducted sensitivity analysis to examine the effects of different combinations. This approach helped us identify key factors influencing NAFLD progression, aiming to use them for future clinical assessment of NAFLD patients.

## Materials and Methods

### Data source

The research utilized data to conduct the study between January 1, 2016, and December 31, 2022 from the Kaohsiung Medical University Hospital Research Database (KMUHRD). KMUHRD is a multicenter database that includes patient medical data from three hospital campuses. The study was conducted according to the Declaration of Helsinki and was approved by the Institutional Review Boards of Kaohsiung Medical University Hospital (Research Ethics Committee No. KMUHIRB-E(I)-20220303), which waived the requirement for written informed consent.

The database included medical records of hospitalization, outpatient care, prescriptions, laboratory tests, medical imaging examinations, operation notes, and death information. The diagnoses were coded based on the International Classification of Diseases, Ninth Revision, Clinical Modification (ICD-9-CM) codes, and International Classification of Diseases, Tenth Revision, Clinical Modification (ICD-10-CM) codes. On the other hand, the procedures were documented using the ICD-9-CM codes and the ICD-10 Procedure Coding System (ICD-10-PCS) codes. The prescription for medications was recorded in KMUHRD according to the Anatomical Therapeutic Chemical (ATC) codes, which included the trade name, active pharmaceutical ingredients, dosage, date of prescription, and drug use period.

### Study design and study population

**Figure 1:**
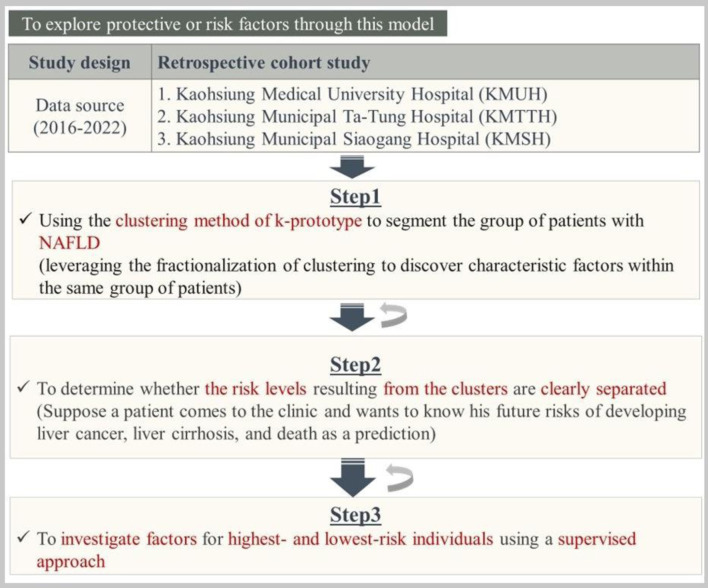
The overall of the study methods.

Figure 1 outlines the retrospective cohort study design, which combines three steps to build multiple models. Step 1 used the k-prototype clustering method to segment NAFLD patients and identify characteristic factors. Step 2 assessed whether risk levels between clusters were clearly separated; if not, reclustering was performed. Step 3 investigated protective or risk factors in the highest- and lowest-risk clusters using a supervised approach, iterating between steps as needed to refine the model.

### Study step 1

#### Study design

In this study, step 1 used the k-prototype algorithm, an unsupervised machine learning method, to define distinct patient clusters. NAFLD patients from the KMUHRD database were screened between January 1, 2017, and December 31, 2021, and those meeting eligibility criteria were included. After confirming the eligible population, cluster analysis was performed. A brief overview of the step 1 design is shown in Supplementary Material 1 (jocmr.elmerjournals.com).

#### Study population

The preliminary study population was defined as diagnosed NAFLD patients confirmed by diagnosis (ICD-9-CM codes 571.8; ICD-10-CM codes K75.81, K76.0). The earliest occurrence between at least one outpatient visit or at least one inpatient visit between the years 2017 and 2021 was considered as the diagnosis date of NAFLD.

Although biopsy was the gold standard for diagnosing NAFLD, clinical practice predominantly used ultrasound, and there were no specific blood tests for direct confirmation of NAFLD, raising concerns about diagnostic accuracy. Therefore, according to Harrison’s Chapter 343 [11], confidence in NAFLD diagnosis was enhanced by identifying associated risk factors (e.g., high body mass index (BMI), insulin resistance/type 2 diabetes mellitus (T2DM), and metabolic syndrome indicators). Additionally, this study included only individuals who had concurrent risk factor-related diseases within 1 year preceding the NAFLD diagnosis date. Therefore, the diagnosis date of NAFLD, along with the presence of the aforementioned risk factors in the previous year, was used to include the patients. The inclusion date was referred to as the population entry date. The NAFLD patients who were aged at least 18 years on the population entry date were enrolled. On the other hand, this study excluded the NAFLD patients without gender or age information throughout the KMUHRD. To limit the potential bias in observational studies, the study population was further screened according to eligibility criteria. Individuals who underwent LT surgery within 365 days before the population entry date or those who underwent bariatric surgery either within 365 days before the population entry date or within 180 days after the population entry date were excluded. Furthermore, to minimize the influence of lingering effects on observed outcome events in this study, patients who had HCC or liver cirrhosis within 1 year before the population entry date were excluded. All inclusion and exclusion criteria are shown in Supplementary Material 2 (jocmr.elmerjournals.com). In study step 1, for every NAFLD patient who was eventually included, the population entry date plus 365 days was denoted as the cluster entry date for each patient. The period from the population entry date to the population entry date plus 365 days (the cluster entry date) was referred to as the “follow-up period”.

#### Data collection

Before the cluster analysis, all available clinical variables from KMUHRD were collected. The follow-up period spanned from the population entry date to the cluster entry date. Demographic information (age, sex) and medical department were taken from the population entry date, while medical history, interventions, and medications were gathered from all records within the follow-up period. Laboratory data and liver echo findings were taken from the closest record to the cluster entry date.

#### Data pre-processing

To minimize investigator-imposed bias, we intentionally limited *a priori* variable selection and adopted a data-driven clustering strategy using high-dimensional EMR data. The detailed data pre-processing steps are summarized in Supplementary Material 3 (jocmr.elmerjournals.com). First, patients’ age, sex, and medical department were directly included in the cluster analysis. Second, in the medical history, medical intervention history, and medication data, variables present in less than 1% of patients were excluded, except for liver cancer and liver cirrhosis, to observe the association between NAFLD patients and liver deterioration. Missing data were labeled as “No,” and liver echo findings without tests were marked as “No test.” All these data were included in the cluster analysis. Third, laboratory data were adjusted, with values like “> 10” or “< 0.1” set to “10” and “0.1,” respectively, and synonymous terms were standardized (e.g., GPT and ALT were unified as ALT). Variables with over 90% missing data were excluded, while those with acceptable missing rates were imputed. Categorical missing values were imputed as “No,” and continuous variables were handled using K-Nearest Neighbors Imputation (KNNI). The imputed datasets were pooled and combined with other data, including demographics, department, history, interventions, medications, and liver echo, resulting in 482 input variables in the final dataset.

#### Statistical analysis

Normality was assessed using the Kolmogorov–Smirnov test. Continuous variables were expressed as medians with interquartile ranges, and categorical variables as counts with percentages. For non-normal data, the Kruskal–Wallis test was used for continuous variables, and the Chi-square or Fisher’s exact test for categorical variables with low expected counts. Data conversion was done in Excel 2016, and Python 3.10.9 was used for multiple imputation and k-prototype clustering analysis.

### Study step 2

#### Study design

In study step 2, retrospective cohort studies were conducted for survival analysis. Patients entered the cohort at the time of NAFLD diagnosis (index date). Baseline clinical variables collected during the first 365 days after the index date (hereafter referred to as the follow-up period) were used for unsupervised clustering. Patients who developed cirrhosis, HCC, underwent bariatric surgery or liver transplantation, or died during this follow-up period were excluded. The landmark (cluster entry) date was defined as 365 days after index, and follow-up until they reached the study endpoint, were lost to follow-up, or until December 31, 2022. The study design for step 2 is shown in Supplementary Material 4 (jocmr.elmerjournals.com) (this study adopted a landmark design with a fixed landmark at 365 days after population entry). This step was aimed at observing the differences in risk levels between different clustering and determining whether the risks among the different clustering had been separated.

#### Study population

The study population in step 2 differed from step 1. Cluster 0 was excluded from the main analyses due to its advanced clinical features at baseline (Supplementary Material 5, jocmr.elmerjournals.com). Patients were also excluded if they developed liver cirrhosis, liver cancer, or experienced mortality from any cause or liver-related causes during the follow-up period. Those who underwent LT or bariatric surgery during the follow-up period were also excluded to maintain result clarity. To ensure at least 1 year of follow-up, patients with a population entry date after December 31, 2020 were also excluded. The remaining patients were included in step 2.

#### Outcome ascertainment

In step 2, outcomes were categorized as primary and secondary, the identification of the outcomes were based on the ICD diagnostic codes available in the KMUHRD, and only events occurring after the landmark date were considered. Patients with a documented diagnosis of liver fibrosis/cirrhosis, HCC, or death prior to the landmark date were excluded from the outcome analyses. Detailed diagnostic codes and outcome ascertainment algorithms are provided in Supplementary Material 6 (jocmr.elmerjournals.com). Primary outcomes included two composites: primary 1 (liver cirrhosis/fibrosis, liver cancer, and all-cause mortality) and primary 2 (liver cirrhosis/fibrosis, liver cancer, and liver-associated mortality). Secondary outcomes consisted of liver cirrhosis/fibrosis, liver cancer, all-cause mortality, and liver-associated mortality, each analyzed separately.

#### Statistical analysis

Survival rates for primary and secondary outcomes were measured in each cluster, with survival curves being plotted using the Kaplan–Meier method and compared using the log-rank test. The incidence rate per 10,000 person-years was used to assess the association between clusters and clinical outcomes. The Cox proportional hazards model estimated the association, reporting hazard ratios (HRs) with 95% CI. A two-tailed P-value of less than 0.05 was considered statistically significant for all tests.

### Study step 3

#### Study design

Step 3 aimed to explore potential factors associated with the highest- and lowest-risk clusters identified in step 2. Patients from these two clusters were initially selected with all available variables included. As illustrated in Supplementary Material 7 (jocmr.elmerjournals.com), step 3 consisted of seven stages, during which variables exhibiting substantial collinearity were identified and removed to reduce redundancy and improve interpretability in subsequent analyses. This post-clustering refinement was data-driven and was conducted without using outcome information or applying *a priori* clinical selection.

#### Study population

The same as step 2 population.

#### Outcome ascertainment

We took the highest risk of the cluster as the outcome to be observed. This allowed us to understand that when the explored factors were combined with the outcome, we could determine under which factors the patient was at the highest risk of falling into the highest-risk cluster. (The supervised model was designed to classify patients into high- versus low-risk phenotypic clusters derived from prior data-driven clustering, with cluster risk levels established through survival analyses. The supervised analysis was exploratory and classification-oriented, with predictors evaluated for their ability to discriminate cluster membership rather than to directly predict liver fibrosis, HCC, or mortality, or to establish causal effects)

#### Statistical analysis

Lasso regression, a type of linear regression with regularization, improves model prediction accuracy and interpretability by reducing complexity and selecting features. Logistic regression estimated the association between factors and outcomes, with results reported as odds ratios (ORs) and 95% CIs; P-values below 0.05 were considered significant. For continuous predictors, ORs were initially estimated per unit increase. To enhance clinical interpretability, these estimates may be interpreted using clinically meaningful increments (e.g., per 10 units), except for continuous variables in which a one-unit increment is clinically substantial. Stepwise logistic regression used a stepwise method to select significant variables, aiming to simplify the model, reduce overfitting, and enhance interpretability by focusing on key predictors. The receiver operating characteristic (ROC) curve evaluated the diagnostic ability of a binary classifier, with the area under the curve (AUC) summarizing its performance. The best cutoff point on the AUC was determined using Youden’s Index.

#### Sensitivity analysis

The first sensitivity analysis involved grouping drugs of the same class and repeating all primary analysis stages in step 3 to compare the outcomes of individual medications versus combined class effects.

In the second sensitivity analysis, since cirrhosis, liver cancer, death, liver transplantation, and bariatric surgery were excluded after clustering in the primary analysis to preserve patient data completeness, it examined whether excluding these factors before clustering would affect the original clustering. The second sensitivity analysis was designed to assess the robustness of the final variable set retained in step 3, rather than to evaluate reproducibility of cluster assignments. This analysis involved excluding these factors occurring within 365 days after the population entry date in step 1, then re-clustering the population to check for differences from the original clusters. Steps 2 and 3 were performed, and the retained variables were compared with those in the primary analysis. Consistency in variables between both analyses would further confirm their importance and validate the clustering approach used in the study.

## Results

### Study step 1

The study population was first defined according to predefined inclusion and exclusion criteria, followed by unsupervised k-prototype clustering to identify distinct NAFLD phenotypes and a comparison of baseline characteristics across the resulting clusters.

#### Eligible study population

**Figure 2:**
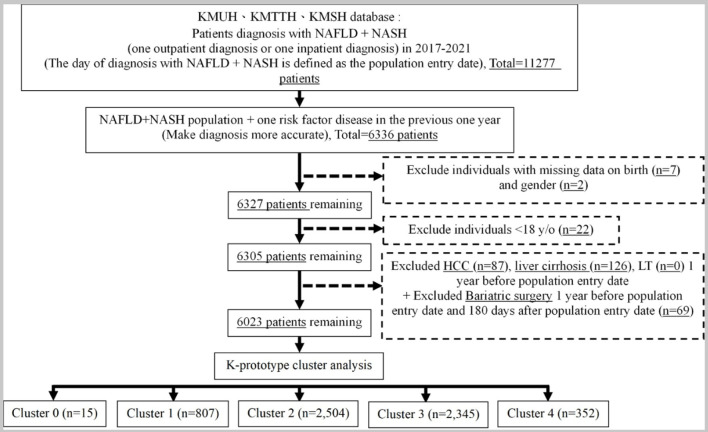
The result in step 1 of the study.

This study included a group diagnosed with NAFLD, following inclusion and exclusion criteria. Finally, the total number of eligible study population was 6,023 patients. The flow chart of study step 1 is illustrated in Figure 2.

#### K-prototype cluster analysis

This subsection evaluates the optimal number of clusters using the elbow method based on changes in within-cluster sum of squares (WCSS). Supplementary Material 8 (jocmr.elmerjournals.com) shows the elbow plot, where WCSS dropped significantly by 3.4% from k = 0 to 1 and leveled off after k = 3. The WCSS decreased by only 1.1% from k = 5 to 6, a smaller change compared to the 1.5% drop from k = 4 to 5. Thus, the elbow point was identified at k = 5. (To assess the stability and robustness of the identified clusters, several complementary approaches were considered. The number of clusters was determined using the elbow method based on within-cluster sum of squares, supporting the selected clustering solution. The resulting clusters demonstrated coherent and clinically interpretable profiles across key demographic, metabolic, and laboratory characteristics, suggesting meaningful separation rather than random partitioning. In addition, the observed gradients in clinical outcomes across clusters were directionally consistent with their baseline risk profiles, providing indirect support for the robustness of the phenotypic structure. Formal quantitative validation metrics, such as bootstrapping or silhouette analysis, were not performed and should be considered in future studies)

#### Baseline characteristics of each cluster

The baseline characteristics of the four clusters (excluding cluster 0 from the discussion) are schematized in Supplementary Material 9 (jocmr.elmerjournals.com). It can be seen that patients in cluster 1 were the most severe, while those in cluster 2 were the mildest group. Detailed information on each cluster is provided in Supplementary Material 10 (jocmr.elmerjournals.com).

### Study step 2

In this step, clinical outcomes were evaluated across the identified clusters by sequentially describing the eligible study population, comparing survival outcomes, summarizing incidence rates, and estimating relative risks using Cox regression analysis.

#### Eligible study population

In step 1, the initial population comprised 6,023 patients. After excluding those with liver cirrhosis (n = 33), HCC (n = 15), LT (n = 0), who died (n = 1), who underwent bariatric surgery (n = 5), cluster 0 (n = 9), and those with a population entry date after December 31, 2020 (n = 972), the remaining population in step 2 was 4,988 patients.

#### Survival analysis of clinical outcomes across clusters

**Figure 3:**
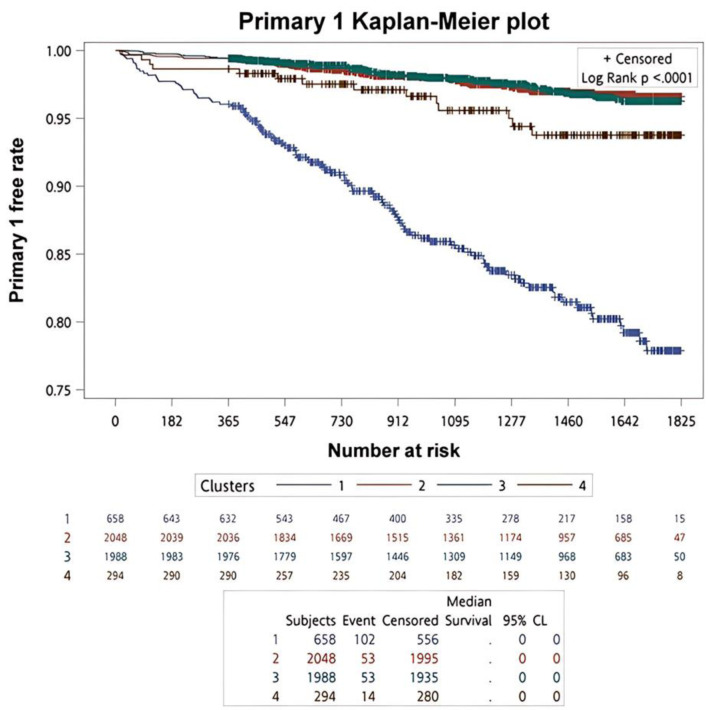
The survival analysis of the primary outcomes for each cluster.

**Figure 4:**
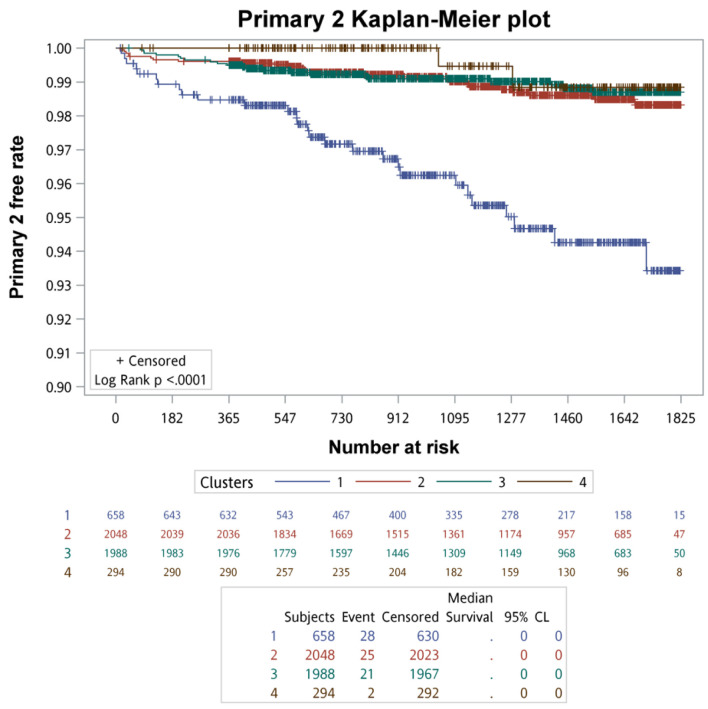
The survival analysis of the secondary outcomes for each cluster.

Kaplan–Meier survival analysis with log-rank testing was used to compare primary and secondary clinical outcomes across the four clusters. The Kaplan–Meier survival curves of primary clinical outcomes across the four clusters are demonstrated in Figure 3, whereas that of secondary clinical outcomes is depicted in Figure 4. In summary of the Kaplan–Meier plot and log-rank test, the survival probability was the lowest in cluster 1 for all outcomes, and it showed significant differences compared to the other clusters. The risk between cluster 2 and cluster 3 showed no significant differences in any outcome comparisons. Additionally, the risk in cluster 4 varied across different outcomes, in primary 1 (LC + HCC + all-cause mortality) and secondary 3 (all-cause mortality) outcomes, the survival probability was the second lowest and it showed significant differences compared to the other clusters, including cluster 2 and cluster 3.

#### Incidence rates of clinical outcomes across clusters

Incidence rates of primary and secondary clinical outcomes across the four clusters are summarized in Table 1. In summary, cluster 1 had the highest incidence rate for any outcome, while cluster 4 ranked second in all-cause mortality (129.35 per 10,000 person-years), and the incidence rates of liver-related outcomes were the lowest, except for HCC. Although the incidence rates of cluster 2 and cluster 3 were similar, a closer look revealed that cluster 2 ranked second in the primary 2, secondary 4, and secondary 1 outcomes, whereas cluster 3 ranked second in the primary 1, secondary 2, and secondary 3 outcomes. Therefore, the incidence rates of liver-related outcomes in cluster 2 were generally higher than those in cluster 3, except for HCC. In terms of all-cause mortality, cluster 3 (46.80 per 10,000 person-years) had a higher rate compared to cluster 2 (44.33 per 10,000 person-years).

**Table 1:** Incidence Rates and Hazard Ratios of Clinical Outcomes Between the Four Clusters

Clinical endpoints	Clusters	Number of events	Follow-up^a^	Incidence rate^b^	Hazard ratio (95% CI)	
					Crude	P-value
Primary 1	2	53	7,171	73.91	1.000 (Reference)	-
	1	102	2,006	508.47	6.918 (4.963, 9.645)	< 0.0001
	3	53	7,001	75.70	1.027 (0.702, 1.502)	0.8920
	4	14	1,002	139.72	1.896 (1.052, 3.417)	0.0332
Primary 2	2	25	7,171	34.86	1.000 (Reference)	-
	1	28	2,006	139.58	3.964 (2.310, 6.803)	< 0.0001
	3	21	7,001	30.00	0.861 (0.482, 1.539)	0.6143
	4	2	1,002	19.96	0.572 (0.135, 2.414)	0.4467
LC	2	19	7,171	26.50	1.000 (Reference)	-
	1	18	2,006	89.73	3.320 (1.741, 6.331)	0.0003
	3	14	7,001	20.00	0.756 (0.379, 1.507)	0.4262
	4	2	1,002	19.96	0.750 (0.175, 3.219)	0.6987
HCC	2	6	7,210	8.32	1.000 (Reference)	-
	1	12	2,024	59.29	7.097 (2.661, 18.928)	< 0.0001
	3	8	7,032	11.38	1.370 (0.475, 3.950)	0.5596
	4	1	1,00	9.97	1.200 (0.145, 9.972)	0.8657
All-cause mortality	2	32	7,219	44.33	1.000 (Reference)	-
	1	85	2,049	414.84	9.554 (6.360, 14.350)	< 0.0001
	3	33	7,052	46.80	1.056 (0.650, 1.718)	0.8249
	4	13	1,005	129.35	2.934 (1.540, 5.589)	0.0011
Liver-associated mortality	2	2	7,219	2.77	1.000 (Reference)	-
	1	4	2,049	19.52	7.459 (1.364, 40.786)	0.0204
	3	0	7,052	0.00	NA	NA
	4	0	1,005	0.00	NA	NA

^a^Follow-ups were presented by person-years. ^b^Incidence rates were presented by per 10,000 person-years. CI: confidence interval; HCC: hepatocellular carcinoma; LC: liver cirrhosis.

#### Cox regression analysis of clinical outcomes across clusters

Based on univariate Cox regression analysis, cluster 2 was used as the reference due to its patients having milder disease severity combined with step 1. HR details are shown in Table 1. For all other outcomes, the risk in cluster 1 was significantly higher compared to cluster 2. The risk in cluster 3 showed no significant differences compared to cluster 2. However, HR was higher in the outcome of primary 1 ((1.027 (0.702, 1.502), P-value: 0.8920), secondary 2 ((1.370 (0.475, 3.950), P-value: 0.5596), and secondary 3 ((1.056 (0.650, 1.718), P-value: 0.8249) when compared to cluster 3 with cluster 2. In cluster 4, the risk was significantly higher compared to cluster 2 for primary 1 (LC + HCC + all-cause mortality) ((1.896 (1.052, 3.417), P-value: 0.0332) and secondary 3 (all-cause mortality) ((2.934 (1.540, 5.589), P-value: 0.0011).

### Study step 3

This subsection summarizes a supervised modeling approach to identify a parsimonious set of key variables that best distinguish the most severe cluster and least severe cluster phenotypes, with the aim of characterizing phenotypic features rather than predicting clinical outcomes.

#### Selection of clusters for supervised comparison

Based on the findings from steps 1 and 2, cluster 2 represented the least severe phenotype, whereas cluster 1 represented the most severe phenotype, and these two clusters were therefore selected for comparison in step 3.

#### The process of selecting variables for the full model

**Figure 5:**
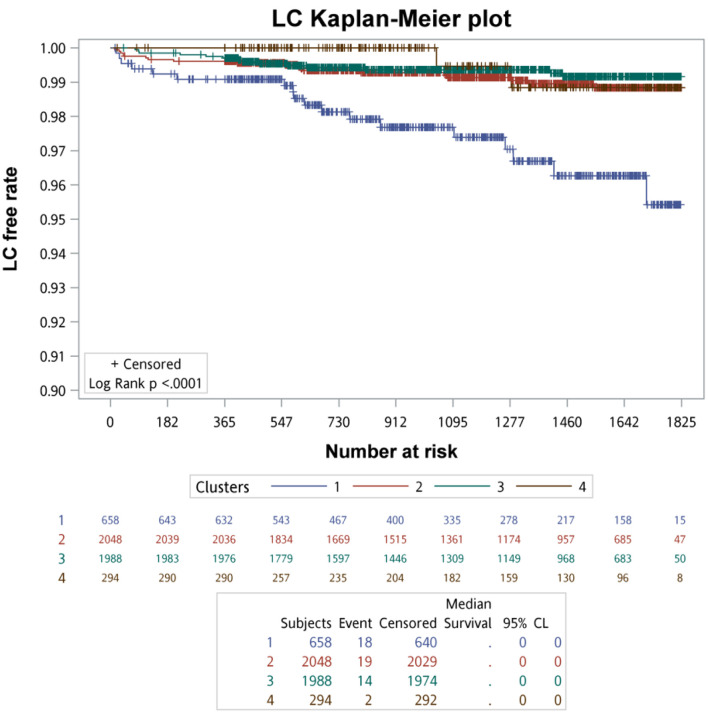
Comparison of the ROC curve between cluster 1 and cluster 2. ROC: receiver operating characteristic.

A supervised modeling framework was applied to compare cluster 1 and cluster 2, beginning with 478 candidate variables and proceeding through seven selection stages to derive a parsimonious set of 17 variables for cluster discrimination which will be separated into explanatory variables, exploratory variables, and unknown variables shown in Table 2 (Associations observed between predictors variables and cluster membership reflect statistical relationships within the data and do not imply causality). The remaining variables (estimate) including age (0.0576), chronic kidney disease (CKD, 1.5086), other urinary system disease (1.9307), sodium chloride (2.3511), metformin (1.3551), partial thromboplastin time, plasma (PTTp, 0.1988), low-density lipoprotein cholesterol (LDL-C, −0.0282), pCO_2_ (−0.1475), glucose level (0.0203), C-reactive protein (CRP, 0.0152), total protein (0.00358), lymphocyte (−0.1564), hemoglobin (Hgb, −1.741), red cell distribution width-standard deviation (RDW-SD, 0.2298), blood urea nitrogen (BUN, 0.1023), estimated glomerular filtration rate (eGFR, −0.0238), and urine creatinine (−0.01) would be calculated using ROC curve model (the outcome would use the cluster 1 as the event cluster). This is shown in Figure 5. Table 2 shows the logistic model estimates. The supervised model demonstrated excellent discrimination for cluster membership (AUC = 0.987). Using the Youden index, an optimal probability cutoff of 0.1648 was identified for separating highest- versus lowest-risk clusters, such that patients with a predicted probability above this threshold, based on the 17 variables, were more likely to be classified into cluster 1, which represented the higher-risk phenotypic group.

**Table 2:** Cluster 1 Compared With Cluster 2 (17 Variables of the Full Model)

Full model	Cluster 1 (n = 658)	Cluster 2 (n = 2,048)	Variables type	Odds ratio, 95% CI	P-value	Estimate (β)
Intercept						12.3011
Age (years)	67.9 (59.70, 75.90)	55.3 (43.65, 63.9)	Explanatory	1.77 (1.51, 2.10)	< 0.0001	0.0576
Chronic kidney disease	137 (20.82)	39 (1.90)	Explanatory	4.521 (2.028, 10.074)	0.0002	1.5086
Other urinary system disease	172 (26.14)	49 (2.39)	Explanatory/exploratory	6.894 (3.285, 14.470)	< 0.0001	1.9307
Sodium chloride	473 (71.88)	378 (18.46)	Unknown/exploratory	10.497 (6.750, 16.325)	< 0.0001	2.3511
Metformin	228 (34.65)	307 (14.99)	Explanatory	3.877 (2.382, 6.311)	< 0.0001	1.3551
PTTp (s)	27.75 (26.56, 29.58)	27.62 (26.87, 28.70)	Explanatory/exploratory	7.31 (3.98, 13.35)	< 0.0001	0.1988
LDL-C (mg/dL)	92.95 (71.00, 111.20)	108.86 (92.45, 125.45)	Explanatory/exploratory	0.75 (0.69, 0.82)	< 0.0001	−0.0282
pCO_2_ (mm Hg)	40.98 (38.00, 43.80)	42.24 (39.86, 44.44)	Explanatory/exploratory	0.23 (0.15, 0.36)	< 0.0001	−0.1475
Glucose level (mg/dL)	123 (104, 159)	109 (101, 124)	Explanatory/exploratory	1.23 (1.16, 1.29)	< 0.0001	0.0203
CRP (mg/L)	11.06 (3.18, 33.27)	8.81 (3.59, 19.56)	Explanatory	1.16 (1.08, 1.26)	< 0.0001	0.0152
Total protein (mg/dL)	17.8 (10.68, 34.56)	16.42 (10.6, 30.2)	Explanatory/exploratory	1.04 (1.01, 1.06)	0.0026	0.00358
Lymphocyte (%)	21.83 (14.60, 27.20)	27.34 (23.44, 30.96)	Explanatory/exploratory	0.21 (0.15, 0.29)	< 0.0001	−0.1564
Hgb (g/dL)	12.20 (11.00, 13.10)	14.23 (13.50, 15.10)	Explanatory/exploratory	0.175 (0.140, 0.220)	< 0.0001	−1.7410
RDW-SD (fL)	43.40 (41.80, 45.60)	42.25 (41.00, 43.52)	Explanatory/exploratory	9.91 (4.66, 21.1)	< 0.0001	0.2298
BUN (mg/dL)	17.10 (13.00, 24.30)	14.11 (12.22, 16.40)	Explanatory/exploratory	2.79 (1.98, 3.87)	< 0.0001	0.1023
eGFR (mL/min/1.73 m^2^)	70.20 (48.80, 88.90)	90.75 (77.30, 104.70)	Explanatory/exploratory	0.78 (0.73, 0.85)	< 0.0001	−0.0238
Urine creatinine (mg/dL)	108.68 (79.44, 143.70)	126.28 (101.08, 151.46)	Explanatory/exploratory	0.90 (0.86, 0.95)	< 0.0001	−0.0100

Odds ratios for continuous variables are presented using clinically meaningful increments (e.g., per 10 units) to enhance clinical interpretability (except for hemoglobin (g/dL), for which a one-unit increment may be clinically substantial). BUN: blood urea nitrogen; CI: confidence interval; CRP: C-reactive protein; eGFR: estimated glomerular filtration rate; Hgb: hemoglobin; LDL-C: low-density lipoprotein cholesterol; PTTp: partial thromboplastin time, plasma; RDW-SD: red cell distribution width-standard deviation.

### Sensitivity analysis

#### The first sensitivity analysis of the model

Since the previous analysis considered medication ingredients independently with diseases, tests, and procedures, the first sensitivity analysis combined medications of the same class. Clusters 1 and 2 were selected, and the step 3 process was rerun to check for differences in variable selection in the final model. The final model included 17 variables shown in Table 3. The result was exactly the same as when the same class of medications was not combined (using these 17 variables to calculate the ROC curve, shown in Supplementary Material 11, jocmr.elmerjournals.com).

**Table 3:** The First Sensitivity Analysis Cluster 1 Compared With Cluster 2 (17 Variables of Full Model)

Variables	Variables type	Odds ratio, 95% CI	P-value	Estimate (β)
Intercept				15.8739
Age (years)	Explanatory	1.77 (1.51, 2.10)	< 0.0001	0.0576
CKD	Explanatory	4.521 (2.028, 10.074)	0.0002	0.7543
Other urinary system disease	Explanatory/exploratory	6.894 (3.285, 14.470)	< 0.0001	0.9653
Sodium chloride	Unknown/exploratory	10.497 (6.750, 16.325)	< 0.0001	1.1756
Metformin	Explanatory	3.877 (2.382, 6.311)	< 0.0001	0.6776
PTTp (s)	Explanatory/exploratory	7.31 (3.98, 13.35)	< 0.0001	0.1988
LDL-C (mg/dL)	Explanatory/exploratory	0.75 (0.69, 0.82)	< 0.0001	−0.0282
pCO_2_ (mm Hg)	Explanatory/exploratory	0.23 (0.15, 0.36)	< 0.0001	−0.1475
Glucose level (mg/dL)	Explanatory/exploratory	1.23 (1.16, 1.29)	< 0.0001	0.0203
CRP (mg/L)	Explanatory	1.16 (1.08, 1.26)	< 0.0001	0.0152
Total protein (mg/dL)	Explanatory/exploratory	1.04 (1.01, 1.06)	0.0026	0.00358
Lymphocyte (%)	Explanatory/exploratory	0.21 (0.15, 0.29)	< 0.0001	−0.1564
Hgb (g/dL)	Explanatory/exploratory	0.175 (0.140, 0.220)	< 0.0001	−1.7410
RDW-SD (fL)	Explanatory/exploratory	9.91 (4.66, 21.1)	< 0.0001	0.2298
BUN (mg/dL)	Explanatory/exploratory	2.79 (1.98, 3.87)	< 0.0001	0.1023
eGFR (mL/min/1.73 m^2^)	Explanatory/exploratory	0.78 (0.73, 0.85)	< 0.0001	−0.0238
Urine creatinine (mg/dL)	Explanatory/exploratory	0.90 (0.86, 0.95)	< 0.0001	−0.0100

Odds ratios for continuous variables are presented using clinically meaningful increments (e.g., per 10 units) to enhance clinical interpretability (except for hemoglobin (g/dL), for which a one-unit increment may be clinically substantial). BUN: blood urea nitrogen; CKD: chronic kidney disease; CRP: C-reactive protein; eGFR: estimated glomerular filtration rate; Hgb: hemoglobin; LDL-C: low-density lipoprotein cholesterol; PTTp: partial thromboplastin time, plasma; RDW-SD: red cell distribution width-standard deviation.

#### The secondary sensitivity analysis of the model

In the secondary sensitivity analysis, as shown in Table 4, after comparing the cluster with almost the highest severity of disease (new cluster 4, n = 505) with the cluster with almost the lowest severity (new cluster 1, n = 1,846) and performing step 2 and step 3, a total of 16 variables were retained. Among these, 10 variables overlapped with those in the primary analysis, including sodium chloride, age, lymphocyte, glucose, RDW-SD, pCO_2_, LDL-C, BUN, CRP, and CKD.

**Table 4:** The Category of the Clusters in the Secondary Sensitivity Analysis

New clusters (seven clusters)	Old clusters (five clusters)					
	0	1	2	3	4	Total
0	11	0	0	0	0	11
	0.18	0	0	0	0	0.18
	100	0	0	0	0	
	100	0	0	0	0	
1	0	16	1,846	139	13	2,014
	0	0.27	30.93	2.33	0.22	33.74
	0	0.79	91.66	6.9	0.65	
	0	2.04	73.99	5.97	3.71	
2	0	0	0	0	315	315
	0	0	0	0	5.28	5.28
	0	0	0	0	100	
	0	0	0	0	90	
3	0	71	1	3	1	76
	0	1.19	0.02	0.05	0.02	1.27
	0	93.42	1.32	3.95	1.32	
	0	9.03	0.04	0.13	0.29	
4	0	505	173	193	8	879
	0	8.46	2.9	3.23	0.13	14.73
	0	57.45	19.68	21.96	0.91	
	0	64.25	6.93	8.29	2.29	
5	0	176	412	883	7	1,478
	0	2.95	6.9	14.79	0.12	24.76
	0	11.91	27.88	59.74	0.47	
	0	22.39	16.51	37.95	2	
6	0	18	63	1109	6	1,196
	0	0.3	1.06	18.58	0.1	20.04
	0	1.51	5.27	92.73	0.5	
	0	2.29	2.53	47.66	1.71	
Total	11	786	2495	2327	350	5,969
	0.18	13.17	41.8	38.98	5.86	100

## Discussion

The findings of this study should be interpreted in three distinct aspects. First, the identified clusters represent clinically meaningful NAFLD phenotypes derived from unsupervised clustering of EMRs, serving primarily as a descriptive framework of disease heterogeneity. Second, the observed differences in long-term outcomes across clusters provide validation of their prognostic separation, supporting the clinical relevance of these phenotypes in risk stratification. However, because HCC and liver-related mortality events were infrequent, the corresponding HR estimates were imprecise and should be interpreted as exploratory. Furthermore, though clusters 2 and 3 exhibited similar outcome risks and survival patterns, the clustering process was driven by multidimensional baseline features rather than clinical outcomes. Consequently, overlap in prognostic profiles does not necessarily imply redundancy in phenotypic classification. These clusters may reflect related or intermediate phenotypes, and their distinction should be interpreted cautiously. Third, the predictors identified in the supervised model should be interpreted as associative features reflecting underlying disease severity, clinical context, or healthcare utilization, rather than as causal risk factors. The supervised 17-variable model was designed to classify patients into phenotypic clusters rather than to predict hard clinical endpoints, and its primary utility lies in facilitating phenotype assignment and baseline risk stratification. Accordingly, associations between cluster membership and subsequent outcomes should be interpreted at the phenotype level. Given the exploratory and classification-oriented nature of the clustering framework, the absence of causal modeling, and the lack of formal internal validation, model performance metrics (e.g., AUC) may be optimistic. Also, the identified cutoff was data-driven and cohort-specific, and therefore should be interpreted as exploratory rather than as a clinically validated decision threshold. The model should therefore be regarded as hypothesis-generating and requires external validation before any consideration of clinical implementation. Nevertheless, the identified variables may help inform future research directions and clinical vigilance in NAFLD.

The variables found in the full model were explored (Especially for continuous variables, ORs reflect rescaling to clinically meaningful increments and indicate relative differences in cluster classification rather than causal effects). The first variable was age. Older age was also a risk factor for liver fat accumulation, higher mortality, and progression to fibrosis and HCC in the elderly [12]. Next, considering CKD, eGFR, total protein, urine creatinine, and BUN, CKD and NAFLD are linked by obesity, metabolic syndrome, insulin resistance, inflammation, and oxidative stress [13]. They exacerbate each other, with greater liver stiffness in NAFLD increasing CKD risk [14]. Advanced fibrosis raises CKD prevalence [15]. CKD in NAFLD leads to lower eGFR [16], higher urine protein, reduced urine creatinine, and increased BUN, indicating kidney dysfunction. Also, cirrhosis further reduces renal function through decreased cardiac output and renal vasoconstriction [17–20].

The next risk factor was other disorders of the urinary system, including urinary tract infection (UTI) and urinary incontinence (UI). NAFLD was associated with recurrent UTI in premenopausal women, independent of metabolic syndrome, suggesting a link in specific populations [21]. However, this study had limitations due to the higher obesity rate in the case group. Further research is needed to confirm the NAFLD-UTI association and explore its link to NAFLD progression. A US study found a strong positive correlation between NAFLD and UI in adult women [22].

Also, another factor CRP, an elevated marker of inflammatory responses, was a strong predictor of NAFLD [23]. The inflammation-damaged immune system also led to a decrease in lymphocyte counts [24]. Also, the relationship between NAFLD and the factor of RDW-SD rises from chronic inflammation and oxidative stress, causing irregular red blood cells [25]. NAFLD patients have higher RDW than non-NAFLD individuals [26], and NASH patients show higher RDW than those with simple steatosis or healthy controls. Higher RDW correlates with disease progression, with advanced fibrosis patients having the highest RDW [27]. The next factors were metformin and glucose levels. Metformin use was more common in cluster 1, which had more severe disease and more T2DM patients, suggesting that metformin use reflects higher disease severity rather than causing NAFLD worsening. Insulin resistance contributes to T2DM and NAFLD, causing increased glucose levels [28]. For NAFLD patients, factors like PPTp and Hgb indicated potential liver deterioration. Cirrhosis, resulting from liver damage, could prolong prothrombin time and cause thrombocytopenia [29], and lead to anemia, reducing Hgb levels [30]. The next factor was LDL-C. NAFLD patients were twice as likely to have plasma lipid abnormalities and more atherogenic lipid subfractions than those without NAFLD [31]. As NAFLD progressed to cirrhosis, serum lipids normalized due to liver synthetic failure [30]. Similarly, our study found that LDL-C levels were lower in cluster 1 compared to cluster 2. The next factor was sodium chloride. This drug usage was higher in cluster 1 due to more hospitalized patients in this group, where it was used for fluid balance or as a diluent. The last factor was pCO_2_. pCO_2_ was lower in cluster 1 than in cluster 2, possibly due to increased respiratory rates from inflammation in worsening NAFLD. Further research is needed to clarify the relationship between sodium chloride, pCO_2_ and NAFLD.

In the lab data, it was found that PTTp, glucose level, RDW-SD, total protein, and BUN were higher in cluster 1 compared to cluster 2, but all were still within the normal range. On the other hand, LDL-C, lymphocyte, Hgb, pCO_2_, urine creatinine, and eGFR were lower in cluster 1 compared to cluster 2, but also within the normal range. Therefore, using these variables in the full model indicated that even if they were slightly elevated or decreased within the normal range, the risk of future deterioration into liver fibrosis, cirrhosis, or liver cancer remained high. Thus, these lab data could serve as a clinical warning. Although the sensitivity analysis revealed substantial variability in cluster assignments, its primary purpose was to evaluate the robustness of the variable selection process implemented in step 3. The substantial overlap between variables retained in the primary and secondary analyses indicates consistency in the identification of key phenotype-driving features. This finding supports the importance of these variables, even in the absence of strict reproducibility of cluster labels. However, it is important to note that established non-invasive risk scores, such as fibrosis-4 (FIB-4) and AST-to-platelet ratio index (APRI), are designed to predict advanced fibrosis and support clinical decision-making using a small set of predefined variables. In contrast, the 17-variable model developed in this study focuses on phenotypic classification based on multidimensional clinical data, aiming to capture heterogeneity among NAFLD patients and to stratify risk at the phenotype level rather than to predict specific clinical outcomes. The proposed model should therefore be considered complementary to existing risk scores, with potential utility in research or population-level risk stratification, rather than a replacement for validated tools in routine clinical practice. Also, from a clinical perspective, the proposed framework uses routinely available demographic and laboratory data from EMRs and does not require specialized imaging or invasive testing, allowing application after an initial period of data accrual following NAFLD diagnosis. Although the model is exploratory and not intended for direct clinical decision-making, phenotype-based risk stratification may support population-level risk assessment and research-oriented patient categorization, with potential to inform closer monitoring in selected subgroups. External validation and prospective evaluation are required before any clinical implementation.

### Strengths and limitations

The strength of this study was the first study to apply unsupervised machine learning combined with model building to predict the disease of NAFLD progression. Also in our study, the secondary sensitivity analysis further confirmed that the clustering method in this study was valid. This multi-model approach used in this study to explore the factors influencing the progression of NAFLD may be extrapolated to other diseases in the future. In this study, we developed an approach to stratify the risk of progression to severe NAFLD-related outcomes, including liver cirrhosis, HCC, all-cause mortality, and liver-related mortality. While this approach represents a promising step toward improved risk stratification, external validation is required before any clinical application. Nevertheless, the proposed framework may inform future research aimed at developing more tailored monitoring and supportive strategies for high-risk NAFLD populations.

This study had several limitations. Firstly, liver biopsy is the gold standard for diagnosing NAFLD but is rarely used in routine practice because of its invasive nature and associated risks. Instead, noninvasive approaches such as ultrasonography and clinical assessment, including evaluation of alcohol consumption, are commonly applied. In this study, NAFLD cases were identified using ICD codes, which may be less definitive than histological confirmation, and information bias due to potential misclassification of ICD-coded diagnoses cannot be excluded. However, the use of the top 10 NAFLD-related comorbidities described by Harrison et al was intended to improve case identification accuracy. In addition, the generalizability of our findings should be interpreted with caution, as the study primarily included outpatients and inpatients with metabolic comorbidities receiving specialist care. Consequently, the identified phenotypes may not be directly applicable to broader NAFLD populations in primary care settings or to lean NAFLD, which may involve distinct pathophysiological mechanisms. Future population-based, primary care-based, and lean NAFLD-focused studies are warranted to validate and extend these findings. Secondly, several laboratory results were excluded from cluster analysis due to significant missing data, with a 90% threshold set to avoid information bias. Other missing values were imputed using multiple imputation. This study included 481 clinical variables for cluster analysis, far more than the 12 to 60 variables used in previous studies, enhancing the reliability of the results. Third, as shown in the timeline schematic in step 2 (Supplementary Material 4, jocmr.elmerjournals.com), this study is subject to potential immortal time bias, as patients were required to survive and remain free of major liver-related events during the first 365 days after NAFLD diagnosis in order to be eligible for clustering and subsequent outcome analyses. Such a design may preferentially exclude early progressors with more aggressive disease, thereby potentially leading to an underestimation of HRs and an overoptimistic assessment of long-term prognosis, particularly in higher-risk phenotypes. To mitigate this issue, we adopted a landmark design with a predefined landmark at 365 days, with follow-up for outcomes initiated thereafter. Consequently, HR estimates should be interpreted as conditional on patients having remained event-free during the first year, and may not be generalizable to individuals experiencing rapid disease progression shortly after diagnosis. Fourth, as with many EMR-based studies, residual confounding cannot be fully excluded. Lifestyle factors, alcohol consumption, and socioeconomic status were not routinely captured in the available EMR data, and these unmeasured factors may have influenced both NAFLD progression and subsequent clinical outcomes. Also, the single-region, single-healthcare system design may limit external validity. Therefore, the observed associations should be interpreted with caution and should not be considered causal. In the future, additional studies in other healthcare settings and populations are needed to support the external applicability of these results.

### Conclusions

This study combined multiple models to explore the potential factors in the progression of NAFLD. In the final model, 17 risk factors were identified, each of which had previously been individually investigated for their association with NAFLD. However, combining these 17 factors could be applied in clinical practice to collect patient data and test the reliability of this scale. If the scale proves to be reliable, it could then be used clinically to alert NAFLD patients to the possibility of disease progression, thereby helping to prevent the worsening of the condition.

## Supplementary Material

Suppl 1 The schematic diagram of step 1 study design.

Suppl 2 The inclusion and exclusion criteria in step 1.

Suppl 3 Flowchart for data preprocessing in step 1 of the study.

Suppl 4 The schematic diagram of step 2 study timeline and landmark design.

Suppl 5 Description of cluster 0.

Suppl 6 The outcome definitions.

Suppl 7 Flow chart of building the model in study step 3.

Suppl 8 Elbow plot for determining the optimal number of clusters.

Suppl 9 The baseline characteristics of the four clusters.

Suppl 10 The total variables of step 1.

Suppl 11 Comparison of the ROC curve for the first sensitivity between cluster 1 and cluster 2.

## Data Availability

Corresponding author (Prof. Chung-Yu Chen) had full access to all the data in the study and takes responsibility for the integrity of the data and the accuracy of the data analysis. The data used in this study were accessed from the Kaohsiung Medical University Hospital database. The data are not publicly available but can be accessed upon reasonable request through the hospital’s data access procedures.

## Abbreviations

aHR: adjusted hazard ratio
AST: aspartate aminotransferase
AUC: area under the curve
BMI: body mass index
BUN: blood urea nitrogen
CAD: coronary artery disease
CI: confidence interval
CKD: chronic kidney disease
EMRs: electronic medical records
HCC: hepatocellular carcinoma
NAFLD: non-alcoholic fatty liver disease
NASH: non-alcoholic steatohepatitis
ROC: receiver operating characteristic
T2DM: type 2 diabetes mellitus
UI: urinary incontinence
UTI: urinary tract infection
