# Supplementary figures and images for: Investigating Factors Influencing Disease Progression in Patients With Non-Alcoholic Fatty Liver Disease
Source: J Clin Med Res. 2026 Feb 28;18(2):83–98. doi: 10.14740/jocmr6424 (PMC12978391; doi:10.14740/jocmr6424)

**Suppl 1.** The schematic diagram of step 1 study design.


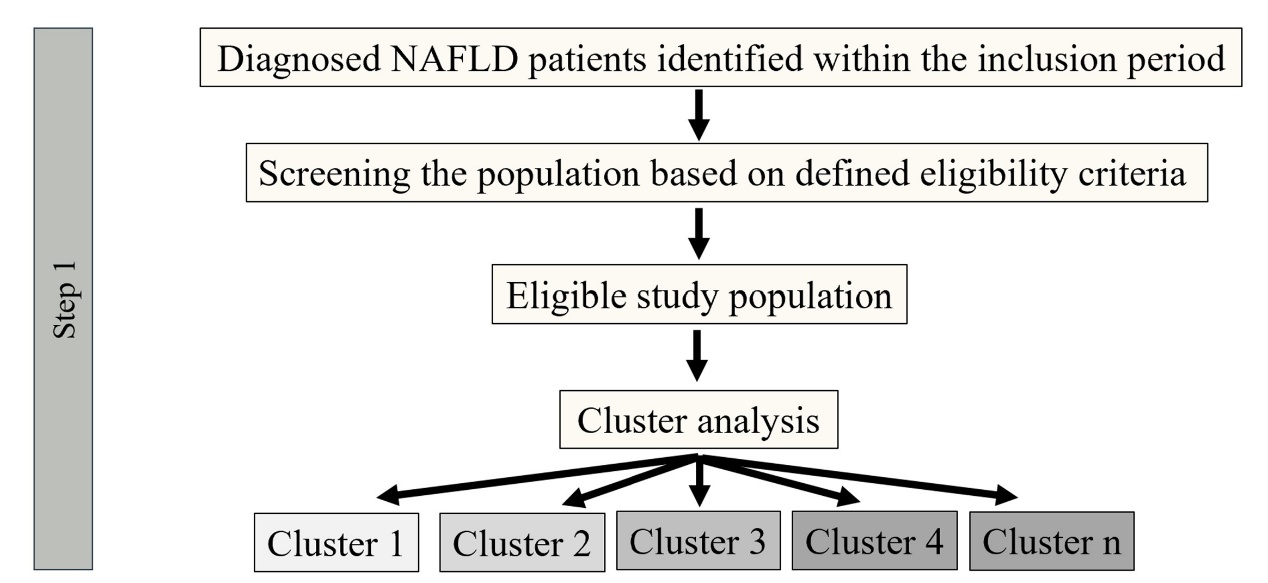

Supplement: Suppl 1 — The schematic diagram of step 1 study design. [file jocmr-18-02-083-s001.docx]

**Suppl 2.** The inclusion and exclusion criteria in step 1.


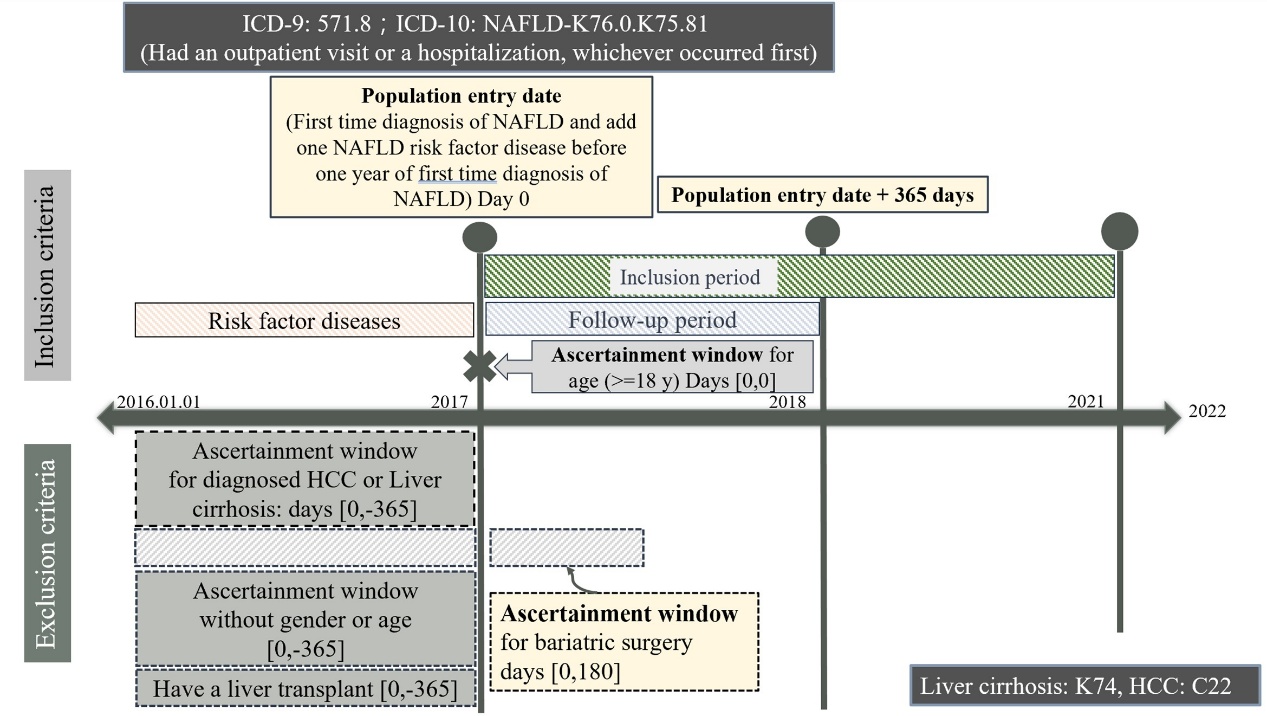

Supplement: Suppl 2 — The inclusion and exclusion criteria in step 1. [file jocmr-18-02-083-s002.docx]

**Suppl 3.** Flowchart for data preprocessing in step 1 of the study.


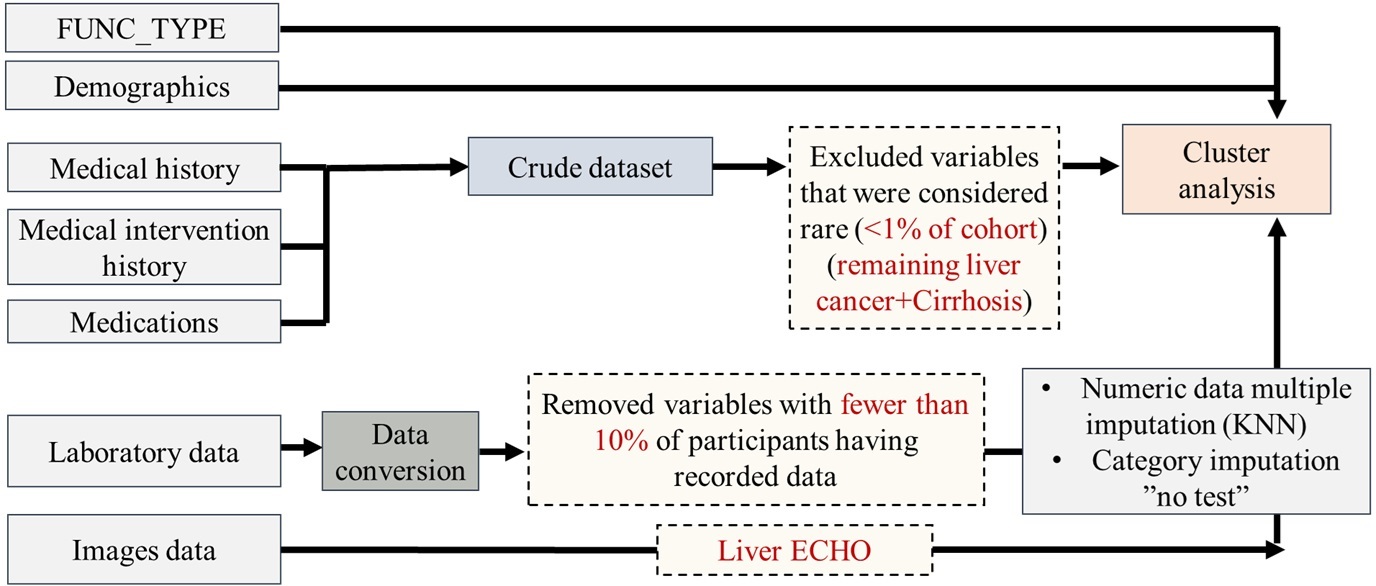

Supplement: Suppl 3 — Flowchart for data preprocessing in step 1 of the study. [file jocmr-18-02-083-s003.docx]

**Suppl 4.** The schematic diagram of step 2 study timeline and landmark design.


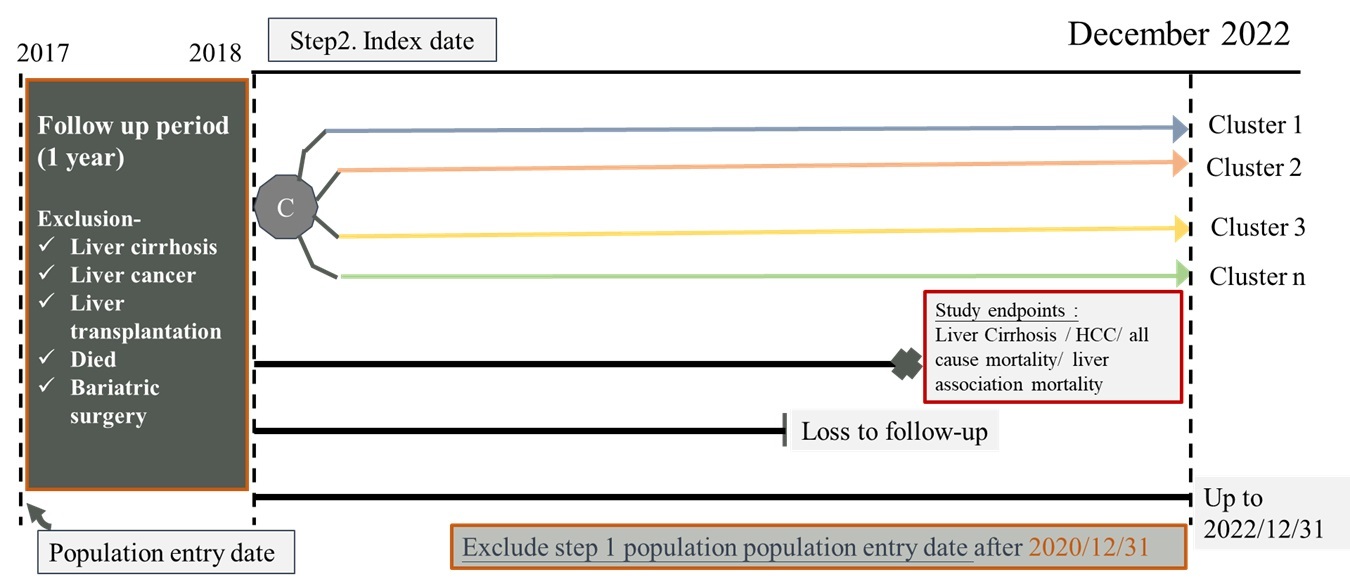

Supplement: Suppl 4 — The schematic diagram of step 2 study timeline and landmark design. [file jocmr-18-02-083-s004.docx]

**Suppl 5.** Description of cluster 0.


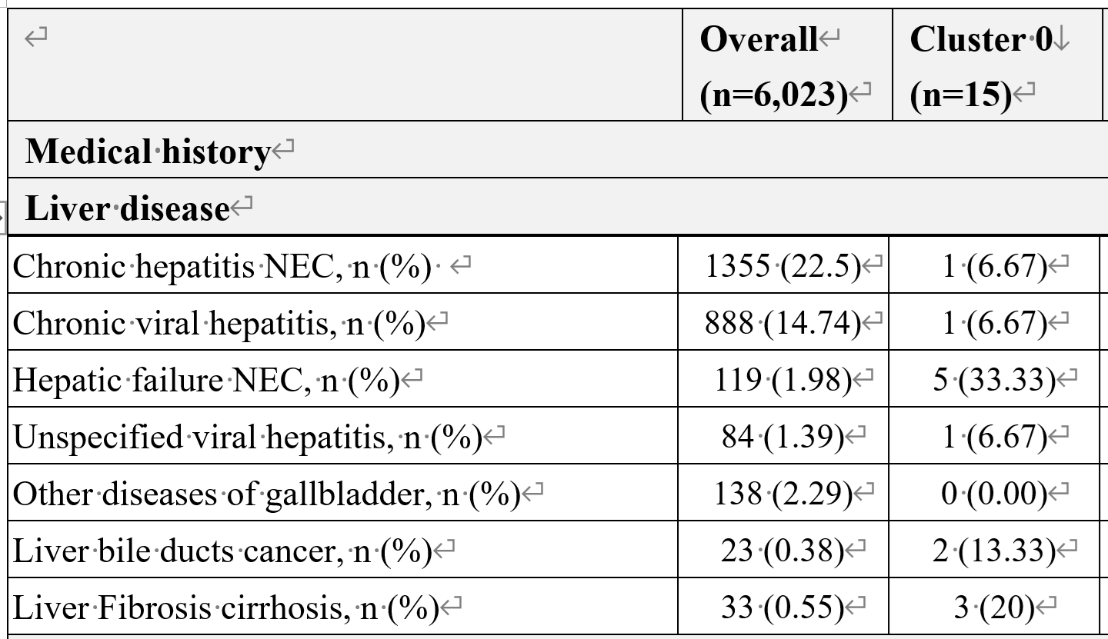

Supplement: Suppl 5 — Description of cluster 0. [file jocmr-18-02-083-s005.docx]

**Suppl 7.** Flow chart of building the model in study step 3.


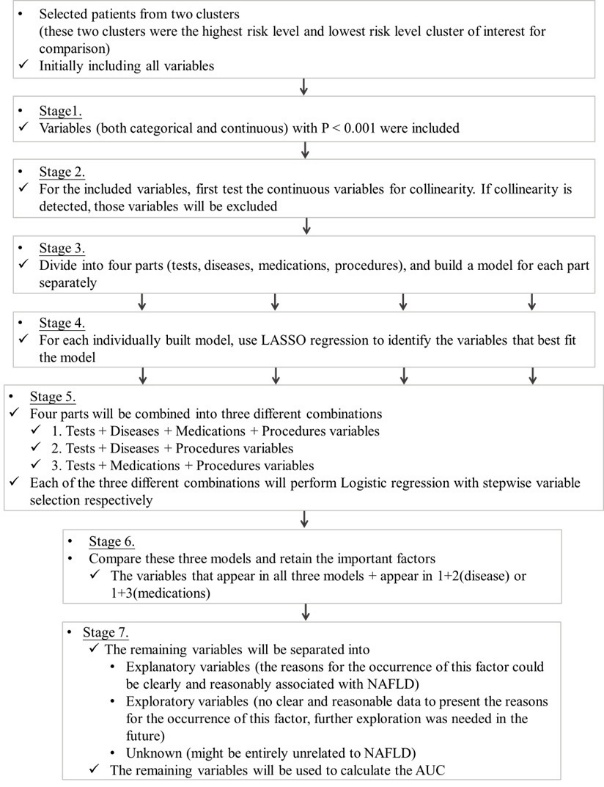

Supplement: Suppl 7 — Flow chart of building the model in study step 3. [file jocmr-18-02-083-s007.docx]

**Suppl 8.** Elbow plot for determining the optimal number of clusters.


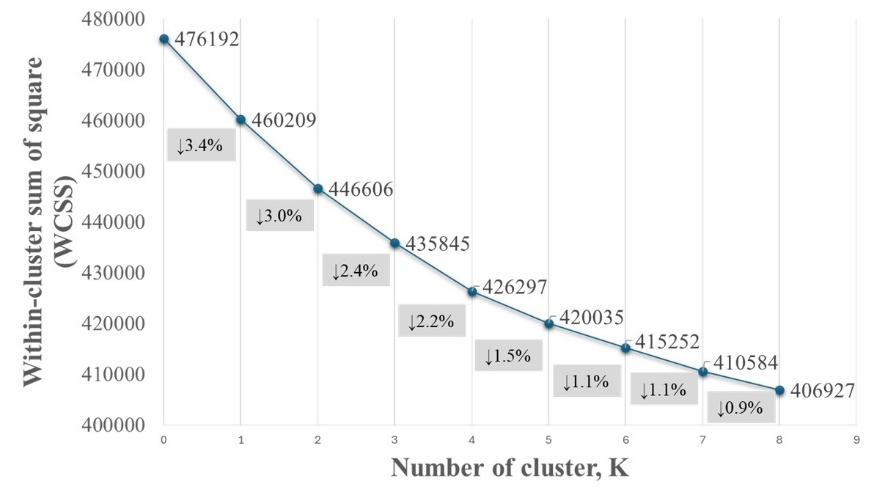

Supplement: Suppl 8 — Elbow plot for determining the optimal number of clusters. [file jocmr-18-02-083-s008.docx]

**Suppl 11.** Comparison of the ROC curve for the first sensitivity between cluster 1 and cluster 2.


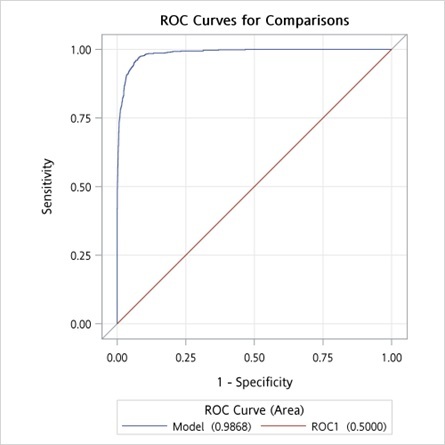

Supplement: Suppl 11 — Comparison of the ROC curve for the first sensitivity between cluster 1 and cluster 2. [file jocmr-18-02-083-s011.docx]
