# Supplementary material for: Investigating Factors Influencing Disease Progression in Patients With Non-Alcoholic Fatty Liver Disease
Source: J Clin Med Res. 2026 Feb 28;18(2):83–98. doi: 10.14740/jocmr6424 (PMC12978391; doi:10.14740/jocmr6424)
Supplement: Suppl 6 — The outcome definitions. [file jocmr-18-02-083-s006.docx]

**Suppl 6.** The outcome definitions.

| **Outcome** | **Definition** | **ICD-9-CM codes** | **ICD-10-CM codes** | **Cause-of-death code** | **Algorithm** |
| --- | --- | --- | --- | --- | --- |
| Liver fibrosis/Liver cirrhosis | Diagnosis of liver fibrosis/Diagnosis of cirrhosis | 571.5 | K74 | – | ≥1 inpatient or  ≥1 outpatient records after landmark |
| HCC | Hepatocellular carcinoma | 155.0 | C22.0 | – | ≥1 inpatient or  ≥1 outpatient records |
| All-cause mortality | Death from any cause | – | – | A value of “1” in the registry was interpreted as indicating death | ≥1 cause-of-death records |
| Liver-related mortality | Death due to liver disease | – | – | 23,C220,C221,K7290,C228 | ≥1 cause-of-death records |
