# Supplementary material for: Investigating Factors Influencing Disease Progression in Patients With Non-Alcoholic Fatty Liver Disease
Source: J Clin Med Res. 2026 Feb 28;18(2):83–98. doi: 10.14740/jocmr6424 (PMC12978391; doi:10.14740/jocmr6424)
Supplement: Suppl 9 — The baseline characteristics of the four clusters. [file jocmr-18-02-083-s009.docx]

**Suppl 9.** The baseline characteristics of the four clusters.

| **Clusters** | **Cluster 1 (n=807)** | **Cluster 2 (n=2,504)** | **Cluster 3 (n=2,345)** | **Cluster 4 (n=352)** |
| --- | --- | --- | --- | --- |
| **Demographics** | - This cluster showed a balanced gender distribution (44.36% men and 55.64% women) and included the oldest patients among all clusters, with a median age of 68.1 years. | - This cluster was predominantly male (60.06%) and had a median age of 55.5 years. | - This cluster had a slightly higher proportion of men (57.61%) compared with women (42.39%), and the median age was 57.8 years. | - This cluster showed a balanced gender distribution (46.88% men vs. 53.13% women) and included the youngest patients among all clusters, with a median age of 54.0 years. |
| **Medication history** | - Nearly all medication usage rates are the highest compared to other groups | - Relatively the healthiest, with nearly the lowest proportions of medication | - The highest medication usage rates compared to other groups include alginic acid (19.40%), rabeprazole (18.81%), glucosamine (1.71%), otilonium bromide (9.77%), mosapride (18.51%), and scopolamine (23.11%) (most of which are gastrointestinal medications) | - The highest medication usage rates compared to other clusters include fenofibrate (8.52%), empagliflozin (4.26%), iron (7.10%), brotizolam (3.98%), and bromazepam (2.56%) |

**Suppl 9.** The baseline characteristics of the four clusters (*continued)*

| **Clusters** | **Cluster 1 (n=807)** | **Cluster 2 (n=2,504)** | **Cluster 3 (n=2,345)** | **Cluster 4 (n=352)** |
| --- | --- | --- | --- | --- |
| **Medical history** | - Nearly all diseases have the highest proportion compared to other groups | - This cluster appeared to be relatively the healthiest, showing nearly the lowest proportions of most diseases. Only a few diseases—such as chronic hepatitis (26.64%) and obesity (3.67%)—had the highest proportions compared with the other clusters.) | - This cluster exhibited notably higher proportions of gastroesophageal reflux and intestinal diseases and was the most inclined to undergo medical examinations among the four clusters | - In this cluster, the highest proportions compared to other clusters include anemias (12.50%), and hemorrhoids and perianal venous thrombosis (4.83%) |
| **Medical intervention  history** | - Also almost the highest, including those for the heart, gallbladder, abdomen, chemotherapy, and lymph nodes surgery-related procedures | - Almost all procedures have the lowest proportions, except for cholecystectomy and lymphatic resection | - The highest number of people underwent upper gastrointestinal endoscopy (21.92%) and lower gastrointestinal endoscopy (10.19%) | - The proportion of surgeries compared to other clusters generally remains at intermediate levels |

**Suppl 9.** The baseline characteristics of the four clusters *(continued)*

| **Clusters** | **Cluster 1 (n=807)** | **Cluster 2 (n=2,504)** | **Cluster 3 (n=2,345)** | **Cluster 4 (n=352)** |
| --- | --- | --- | --- | --- |
| **Liver echo** | - The lowest proportion had a liver echo grade of severe (2.97%) | - The lowest proportion of participants had a mild liver echogenicity grade (6.51%), whereas the highest proportion had no liver echo test performed (72.04%) | - The highest proportion had a liver echo grade of Severe (5.46%). The highest proportion had a liver echo grade of Mild (13.13%) |  |
