# Supplementary material for: Investigating Factors Influencing Disease Progression in Patients With Non-Alcoholic Fatty Liver Disease
Source: J Clin Med Res. 2026 Feb 28;18(2):83–98. doi: 10.14740/jocmr6424 (PMC12978391; doi:10.14740/jocmr6424)
Supplement: Suppl 10 — The total variables of step 1. [file jocmr-18-02-083-s010.docx]

**Suppl 10.** The total variables of step 1.

|  | **Overall**  **(n=6,008)** | **Cluster1 (n=807)** | **Cluster2 (n=2,504)** | **Cluster3 (n=2,345)** | **Cluster4**  **(n=352)** | ***p-value*** |
| --- | --- | --- | --- | --- | --- | --- |
| **Demographics** |  |  |  |  |  | |
| **Age** |  |  |  |  |  |  |
| Age (years), median (IQR) | 58.1  (46.5, 66.5) | 68.1  (60.1, 75.6) | 55.5  (43.9, 64.2) | 57.8  (46.1, 65.3) | 54.0  (45.4, 64.5) | <.0001 |
| <65, n (%) | 4237 (70.52) | 312 (38.66) | 1927 (76.96) | 1731 (73.82) | 267 (75.85) | <.0001 |
| 65-74, n (%) | 1265 (21.06) | 277 (34.32) | 454 (18.13) | 479 (20.43) | 55 (15.63) | <.0001 |
| 75-84, n (%) | 423 (7.04) | 161 (19.95) | 112 (4.47) | 125 (5.33) | 25 (7.10) | <.0001 |
| ≥85, n (%) | 83 (1.38) | 57 (7.06) | 11 (0.44) | 10 (0.43) | 5 (1.42) | <.0001 |
| **Gender** |  |  |  |  |  | <.0001 |
| Male, n (%) | 3378 (56.23) | 358 (44.36) | 1504 (60.06) | 1351 (57.61) | 165 (46.88) |  |
| Female, n (%) | 2630 (43.77) | 449 (55.64) | 1000 (39.94) | 994 (42.39) | 187 (53.13) |  |

| **Medical departments on population entry date** | <.0001 |
| --- | --- |

| Family medicine, n (%) | 401 (6.67) | 10 (1.24) | 174 (6.95) | 196 (8.36) | 21 (5.97) | <.0001 |
| --- | --- | --- | --- | --- | --- | --- |
| Internal medicine, n (%) | 200 (3.33) | 35 (4.34) | 72 (2.88) | 88 (3.75) | 5 (1.42) | 0.0240 |
| Surgery, n (%) | 329 (5.48) | 75 (9.29) | 165 (6.59) | 69 (2.94) | 20 (5.68) | <.0001 |
| Paediatrics, n (%) | 14 (0.23) | 1 (0.12) | 3 (0.12) | 9 (0.38) | 1 (0.28) | 0.2322 |
| Gynaecology and obstetrics, n (%) | 6 (0.10) | 2 (0.25) | 2 (0.08) | 2 (0.09) | 0 (0.00) | 0.5713 |
| Orthopaedics, n (%) | 15 (0.25) | 3 (0.37) | 5 (0.20) | 3 (0.13) | 4 (1.14) | 0.0041 |

**Suppl 10.** The total variables of step 1 *(continued)*

|  | **Overall**  **(n=6,008)** | **Cluster 1(n=807)** | **Cluster2(n=2,504)** | **Cluster3(n=2,345)** | **Cluster4(n=352)** | ***p-value*** |
| --- | --- | --- | --- | --- | --- | --- |
| Neurosurgery, n (%) | 21 (0.35) | 5 (0.62) | 7 (0.28) | 8 (0.34) | 1 (0.28) | 0.5240 |
| Urology, n (%) | 54 (0.90) | 15 (1.86) | 12 (0.48) | 24 (1.02) | 3 (0.85) | 0.0033 |
| Otolaryngology, n (%) | 60 (1.00) | 17 (2.11) | 27 (1.08) | 11 (0.47) | 5 (1.42) | 0.0006 |
| Ophthalmology, n (%) | 1 (0.02) | 0 (0.00) | 1 (0.04) | 0 (0.00) | 0 (0.00) | 1.0000 |
| Dermatology, n (%) | 3 (0.05) | 0 (0.00) | 2 (0.08) | 1 (0.04) | 0 (0.00) | 1.0000 |
| Neurology, n (%) | 184 (3.06) | 27 (3.35) | 24 (0.96) | 122 (5.20) | 11 (3.13) | <.0001 |
| Psychiatry, n (%) | 10 (0.17) | 1 (0.12) | 3 (0.12) | 4 (0.17) | 2 (0.57) | 0.2609 |
| Rehabilitation medicine, n (%) | 4 (0.07) | 0 (0.00) | 0 (0.00) | 4 (0.17) | 0 (0.00) | 0.1664 |
| Plastic surgery, n (%) | 12 (0.20) | 4 (0.50) | 2 (0.08) | 5 (0.21) | 1 (0.28) | 0.1166 |
| Emergency medicine, n (%) | 9 (0.15) | 1 (0.12) | 5 (0.20) | 2 (0.09) | 1 (0.28) | 0.0004 |
| Occupational medicine, n (%) | 154 (2.56) | 5 (0.62) | 76 (3.04) | 60 (2.56) | 13 (3.69) | 0.0010 |
| Dentistry, n (%) | 6 (0.10) | 1 (0.12) | 4 (0.16) | 1 (0.04) | 0 (0.00) | 0.6180 |
| Traditional chinese medicine, n (%) | 1 (0.02) | 0 (0.00) | 1 (0.04) | 0 (0.00) | 0 (0.00) | 1.0000 |
| Gastroenterology, n (%) | 3635 (60.50) | 366 (45.35) | 1629 (65.06) | 1433 (61.11) | 207 (58.81) | <.0001 |
| Cardiology, n (%) | 262 (4.36) | 32 (3.97) | 127 (5.07) | 89 (3.80) | 14 (3.98) | 0.1537 |
| Pulmonary medicine, n (%) | 72 (1.20) | 30 (3.72) | 20 (0.80) | 19 (0.81) | 3 (0.85) | <.0001 |
| Nephrology, n (%) | 98 (1.63) | 22 (2.73) | 13 (0.52) | 57 (2.43) | 6 (1.70) | <.0001 |
| Rheumatology and immunology, n (%) | 13 (0.22) | 4 (0.50) | 3 (0.12) | 6 (0.26) | 0 (0.00) | 0.2241 |
| Haematology and oncology, n (%) | 26 (0.43) | 16 (1.98) | 4 (0.16) | 4 (0.17) | 2 (0.57) | <.0001 |

**Suppl 10. The total variables of step 1 (continued)**

|  | **Overall**  **(n=6,008)** | **Cluster1 (n=807)** | **Cluster2 (n=2,504)** | **Cluster3 (n=2,345)** | **Cluster4 (n=352)** | ***p-value*** |
| --- | --- | --- | --- | --- | --- | --- |
| Endocrinology, n (%) | 131 (2.18) | 29 (3.59) | 33 (1.32) | 58 (2.47) | 11 (3.13) | 0.0003 |
| Infectious diseases, n (%) | 109 (1.81) | 48 (5.95) | 15 (0.60) | 34 (1.45) | 12 (3.41) | <.0001 |
| Geriatrics and gerontology, n (%) | 14 (0.23) | 4 (0.50) | 6 (0.24) | 3 (0.13) | 1 (0.28) | 0.2418 |
| Colorectal surgery, n (%) | 65 (1.08) | 25 (3.10) | 28 (1.12) | 8 (0.34) | 4 (1.14) | <.0001 |
| Cardiovascular surgery, n (%) | 5 (0.08) | 2 (0.25) | 1 (0.04) | 1 (0.04) | 1 (0.28) | 0.1157 |
| Thoracic surgery, n (%) | 28 (0.47) | 9 (1.12) | 11 (0.44) | 8 (0.34) | 0 (0.00) | 0.0692 |
| Digestive surgery, n (%) | 64 (1.07) | 17 (2.11) | 28 (1.12) | 16 (0.68) | 3 (0.85) | 0.0082 |
| Radiation oncology, n (%) | 1 (0.02) | 1 (0.12) | 0 (0.00) | 0 (0.00) | 0 (0.00) | 0.1949 |
| Oral and maxillofacial surgery, n (%) | 1 (0.02) | 0 (0.00) | 1 (0.04) | 0 (0.00) | 0 (0.00) | 1.0000 |

**Suppl 10.** The total variables of step 1 *(continued)*

| **Medical history** | **Overall**  **(n=6,008)** | **Cluster1 (n=807)** | | | | **Cluster2 (n=2,504)** | | | **Cluster3 (n=2,345)** | | | **Cluster4 (n=352)** | | | ***p-value*** | | |
| --- | --- | --- | --- | --- | --- | --- | --- | --- | --- | --- | --- | --- | --- | --- | --- | --- | --- |
| **Liver disease** | | | | | | | | | | | | | | | | | |
| Chronic hepatitis NEC, n (%) | | | 1354 (22.54) | | 131 (16.23) | | | 667 (26.64) | | | 492 (20.98) | | | 64 (18.18) | | | <.0001 |
| Chronic viral hepatitis, n (%) | | | 887 (14.76) | | 94 (11.65) | | | 394 (15.73) | | | 341 (14.54) | | | 58 (16.48) | | | 0.0291 |
| Hepatic failure NEC, n (%) | | | 114 (1.90) | | 33 (4.09) | | | 18 (0.72) | | | 47 (2.00) | | | 16 (4.55) | | | <.0001 |
| Unspecified viral hepatitis, n (%) | | | 83 (1.38) | | 27 (3.35) | | | 18 (0.72) | | | 33 (1.41) | | | 5 (1.42) | | | <.0001 |
| Other diseases of gallbladder, n (%) | | | 138 (2.30) | | 16 (1.98) | | | 69 (2.76) | | | 42 (1.79) | | | 11 (3.13) | | | 0.0916 |
| Liver bile ducts cancer, n (%) | | | 21 (0.35) | | 9 (1.12) | | | 5 (0.20) | | | 7 (0.30) | | | 0 (0.00) | | | 0.0054 |
| Liver Fibrosis cirrhosis, n (%) | | | 30 (0.50) | | 13 (1.61) | | | 3 (0.12) | | | 12 (0.51) | | | 2 (0.57) | | | <.0001 |
| **Metabolic diseases** | | | | | | | | | | | | | | | | | |
| Disorders lipidemias, n (%) | | | 3984 (66.31) | | 409 (50.68) | | | 1634 (65.26) | | | 1714 (73.09) | | | 227 (64.49) | | | <.0001 |
| NAFLD, n (%) | | | 6008 (100.0) | | 807 (100.00) | | | 2504 (100.00) | | | 2345 (100.00) | | | 352 (100.00) | | | NA |
| Type 2 diabetes mellitus, n (%) | | | 1923 (32.01) | | 431 (53.41) | | | 593 (23.68) | | | 778 (33.18) | | | 121 (34.38) | | | <.0001 |
| Other Specified DM, n (%) | | | 338 (5.63) | | 78 (9.67) | | | 63 (2.52) | | | 167 (7.12) | | | 30 (8.52) | | | <.0001 |
| Other Nontoxic goiter, n (%) | | | 200 (3.33) | | 24 (2.97) | | | 84 (3.35) | | | 75 (3.20) | | | 17 (4.83) | | | 0.4059 |
| Other metabolic disease, n (%) | | | 72 (1.20) | | 9 (1.12) | | | 23 (0.92) | | | 31 (1.32) | | | 9 (2.56) | | | 0.0578 |
| Elevated blood glucose level, n (%) | | | 319 (5.31) | | 24 (2.97) | | | 82 (3.27) | | | 193 (8.23) | | | 20 (5.68) | | | <.0001 |
| Other Hypothyroidism, n (%) | | | 128 (2.13) | | 22 (2.73) | | | 45 (1.80) | | | 54 (2.30) | | | 7 (1.99) | | | 0.3801 |
| DM due to underlying disease, n (%) | | | 153 (2.55) | | 57 (7.06) | | | 27 (1.08) | | | 63 (2.69) | | | 6 (1.70) | | | <.0001 |
| Obesity, n (%) | | | 180 (3.00) | | 11 (1.36) | | | 92 (3.67) | | | 67 (2.86) | | | 10 (2.84) | | | 0.0091 |
| **Suppl 10. The total variables of step 1 (continued)** | | | | | | | | | | | | | | | | | |
| **Medical history** | **Overall**  **(n=6,008)** | **Cluster1 (n=807)** | | | | **Cluster2 (n=2,504)** | | | **Cluster3 (n=2,345)** | | | **Cluster4 (n=352)** | | | ***p-value*** | | |
| Thyrotoxicosis, n (%) | 71 (1.18) | | | 9 (1.12) | | | 27 (1.08) | | | 28 (1.19) | | | 7 (1.99) | | | 0.5269 | |
| **Cardiovascular disorders** | | | | | | | | | | | | | | | | | |
| Primary hypertension, n (%) | 2322 (38.65) | | | 502 (62.21) | | | 788 (31.47) | | | 894 (38.12) | | | 138 (39.20) | | | <.0001 | |
| Hypertensive heart disease, n (%) | 671 (11.17) | | | 114 (14.13) | | | 282 (11.26) | | | 237 (10.11) | | | 38 (10.80) | | | 0.0199 | |
| Chronic ischemic heart, n (%) | 495 (8.24) | | | 108 (13.38) | | | 166 (6.63) | | | 198 (8.44) | | | 23 (6.53) | | | <.0001 | |
| Angina pectoris, n (%) | 165 (2.75) | | | 35 (4.34) | | | 62 (2.48) | | | 60 (2.56) | | | 8 (2.27) | | | 0.0302 | |
| Atrial fibrillation and flutter, n (%) | 149 (2.48) | | | 47 (5.82) | | | 47 (1.88) | | | 45 (1.92) | | | 10 (2.84) | | | <.0001 | |
| Heart failure, n ( %) | 181 (3.01) | | | 79 (9.79) | | | 50 (2.00) | | | 43 (1.83) | | | 9 (2.56) | | | <.0001 | |
| Abnormalities of heart beat, n ( %) | 135 (2.25) | | | 29 (3.59) | | | 52 (2.08) | | | 45 (1.92) | | | 9 (2.56) | | | 0.0403 | |
| Hypertensive chronic kidney disease, n ( %) | 65 (1.08) | | | 49 (6.07) | | | 3 (0.12) | | | 9 (0.38) | | | 4 (1.14) | | | <.0001 | |
| Other cardiac arrhythmias, n ( %) | 275 (4.58) | | | 51 (6.32) | | | 95 (3.79) | | | 108 (4.61) | | | 21 (5.97) | | | 0.0136 | |
| Acute myocardial infarction, n ( %) | 77 (1.28) | | | 18 (2.23) | | | 28 (1.12) | | | 29 (1.24) | | | 2 (0.57) | | | 0.0521 | |
| Presence of cardiac and vascular implants and grafts, n ( %) | 65 (1.08) | | | 25 (3.10) | | | 6 (0.24) | | | 26 (1.11) | | | 8 (2.27) | | | <.0001 | |
| **Cerebrovascular diseases** | | | | | | | | | | | | | | | | | |
| Other cerebrovascular diseases, n ( %) | 151 (2.51) | | | 32 (3.97) | | | 25 (1.00) | | | 86 (3.67) | | | 8 (2.27) | | | <.0001 | |
| Sequelae of cerebrovascular disease, n ( %) | 68 (1.13) | | | 20 (2.48) | | | 11 (0.44) | | | 31 (1.32) | | | 6 (1.70) | | | <.0001 | |
| Cerebral infarction, n ( %) | 182 (3.03) | | | 46 (5.70) | | | 45 (1.80) | | | 76 (3.24) | | | 15 (4.26) | | | <.0001 | |
| Transient cerebral ischemic, n ( %) | 93 (1.55) | | | 13 (1.61) | | | 12 (0.48) | | | 64 (2.73) | | | 4 (1.14) | | | <.0001 | |
| **Gastrointestinal diseases** | | | | | | | | | | | | | | | | | |
| Suppl 10. The total variables of step 1 (continued) | | | | | | | | | | | | | | | | | |
|  | **Overall (n=6,008)** | | | **Cluster1 (n=807)** | | | **Cluster2 (n=2,504)** | | | **Cluster3 (n=2,345)** | | | **Cluster4 (n=352)** | | | ***p-value*** | |
| Peptic ulcer, n (%) | 571 (9.50) | | | 119 (14.75) | | | 184 (7.35) | | | 231 (9.85) | | | 37 (10.51) | | | <.0001 | |
| Cholelithiasis, n (%) | 626 (10.42) | | | 128 (15.86) | | | 275 (10.98) | | | 197 (8.40) | | | 26 (7.39) | | | <.0001 | |
| Other disease of digestive system, n (%) | 274 (4.56) | | | 71 (8.80) | | | 55 (2.20) | | | 129 (5.50) | | | 19 (5.40) | | | <.0001 | |
| Other disease digestive and abdomen, n (%) | 106 (1.76) | | | 24 (2.97) | | | 17 (0.68) | | | 58 (2.47) | | | 7 (1.99) | | | <.0001 | |
| Gastro esophageal reflux, n (%) | 1527 (25.42) | | | 230 (28.50) | | | 385 (15.38) | | | 811 (34.58) | | | 101 (28.69) | | | <.0001 | |
| Other and unspecified noninfective gastroenteritis and colitis, n (%) | 135 (2.25) | | | 39 (4.83) | | | 30 (1.20) | | | 60 (2.56) | | | 6 (1.70) | | | <.0001 | |
| Duodenal ulcer, n (%) | 278 (4.63) | | | 58 (7.19) | | | 40 (1.60) | | | 164 (6.99) | | | 16 (4.55) | | | <.0001 | |
| Other diseases of stomach and duodenum, n (%) | 104 (1.73) | | | 20 (2.48) | | | 33 (1.32) | | | 45 (1.92) | | | 6 (1.70) | | | 0.1299 | |
| Gastritis and duodenitis, n (%) | 330 (5.49) | | | 73 (9.05) | | | 100 (3.99) | | | 136 (5.8) | | | 21 (5.97) | | | <.0001 | |
| Other functional intestinal disorders, n (%) | 313 (5.21) | | | 77 (9.54) | | | 79 (3.15) | | | 135 (5.76) | | | 22 (6.25) | | | <.0001 | |
| Gastric ulcer, n (%) | 233 (3.88) | | | 72 (8.92) | | | 62 (2.48) | | | 82 (3.50) | | | 17 (4.83) | | | <.0001 | |
| Other diseases of biliary tract, n (%) | 61 (1.02) | | | 28 (3.47) | | | 16 (0.64) | | | 14 (0.60) | | | 3 (0.85) | | | <.0001 | |
| Benign neoplasm of colon, rectum, anus and anal canal, n (%) | 66 (1.10) | | | 8 (0.99) | | | 25 (1.00) | | | 28 (1.19) | | | 5 (1.42) | | | 0.8377 | |
| Benign neoplasm digestive, n (%) | 106 (1.76) | | | 11 (1.36) | | | 16 (0.64) | | | 76 (3.24) | | | 3 (0.85) | | | <.0001 | |
| Other diseases of intestine, n (%) | 150 (2.50) | | | 18 (2.23) | | | 39 (1.56) | | | 85 (3.62) | | | 8 (2.27) | | | <.0001 | |
| Functional dyspepsia, n (%) | 242 (4.03) | | | 41 (5.08) | | | 76 (3.04) | | | 107 (4.56) | | | 18 (5.11) | | | 0.0093 | |
| Irritable bowel syndrome, n (%) | 128 (2.13) | | | 16 (1.98) | | | 45 (1.80) | | | 57 (2.43) | | | 10 (2.84) | | | 0.3498 | |
| Suppl 10. The total variables of step 1 (continued) | | | | | | | | | | | | | | | | | |
|  | **Overall (n=6,008)** | | | **Cluster1 (n=807)** | | | **Cluster2 (n=2,504)** | | | **Cluster3 (n=2,345)** | | | **Cluster4 (n=352)** | | | ***p-value*** | |
| Hemorrhoids and perianal venous thrombosis, n (%) | 207 (3.45) | | | 29 (3.59) | | | 65 (2.60) | | | 96 (4.09) | | | 17 (4.83) | | | 0.0149 | |
| Acute pancreatitis, n (%) | 115 (1.91) | | | 33 (4.09) | | | 36 (1.44) | | | 40 (1.71) | | | 6 (1.70) | | | <.0001 | |
| Infectious gastroenteritis and colitis, n (%) | 64 (1.07) | | | 17 (2.11) | | | 12 (0.48) | | | 28 (1.19) | | | 7 (1.99) | | | 0.0002 | |
| **General diseases** | | | | | | | | | | | | | | | | | |
| Disorders of vestibular function, n (%) | 212 (3.53) | | | 28 (3.47) | | | 75 (3.00) | | | 95 (4.05) | | | 14 (3.98) | | | 0.2416 | |
| Other headache syndromes, n (%) | 78 (1.30) | | | 8 (0.99) | | | 29 (1.16) | | | 34 (1.45) | | | 7 (1.99) | | | 0.4390 | |
| Other Anemias, n (%) | 354 (5.89) | | | 72 (8.92) | | | 32 (1.28) | | | 206 (8.78) | | | 44 (12.50) | | | <.0001 | |
| Contact with and (suspected) exposure to communicable diseases, n (%) | 255 (4.24) | | | 66 (8.18) | | | 66 (2.64) | | | 106 (4.52) | | | 17 (4.83) | | | <.0001 | |
| Dizziness and giddiness, n (%) | 283 (4.71) | | | 68 (8.43) | | | 81 (3.23) | | | 111 (4.73) | | | 23 (6.53) | | | <.0001 | |
| Other disorders of fluid, electrolyte and acid-base balance, n (%) | 189 (3.15) | | | 96 (11.90) | | | 25 (1.00) | | | 54 (2.30) | | | 14 (3.98) | | | <.0001 | |
| Pain in throat and chest, n (%) | 359 (5.98) | | | 64 (7.93) | | | 131 (5.23) | | | 139 (5.93) | | | 25 (7.10) | | | 0.0326 | |
| Encounter for immunization, n (%) | 930 (15.48) | | | 130 (16.11) | | | 348 (13.90) | | | 396 (16.89) | | | 56 (15.91) | | | 0.0346 | |
| Encounter for screening for malignant neoplasms, n (%) | 1082 (18.01) | | | 144 (17.84) | | | 336 (13.42) | | | 526 (22.43) | | | 76 (21.59) | | | <.0001 | |
| Encounter for screening disease, n (%) | 129 (2.15) | | | 5 (0.62) | | | 58 (2.32) | | | 58 (2.47) | | | 8 (2.27) | | | 0.0146 | |
| Fever of other and unknown origin, n (%) | 179 (2.98) | | | 77 (9.54) | | | 24 (0.96) | | | 67 (2.86) | | | 11 (3.13) | | | <.0001 | |
| Encounter other aftercare, n (%) | 188 (3.13) | | | 95 (11.77) | | | 40 (1.60) | | | 46 (1.96) | | | 7 (1.99) | | | <.0001 | |
| Suppl 10. The total variables of step 1 (continued) | | | | | | | | | | | | | | | | | |
|  | **Overall (n=6,008)** | | | **Cluster1 (n=807)** | | | **Cluster2 (n=2,504)** | | | **Cluster3 (n=2,345)** | | | **Cluster4 (n=352)** | | | ***p-value*** | |
| Encounter other special exam, n (%) | 92 (1.53) | | | 29 (3.59) | | | 32 (1.28) | | | 27 (1.15) | | | 4 (1.14) | | | <.0001 | |
| Dorsalgia, n (%) | 311 (5.18) | | | 62 (7.68) | | | 82 (3.27) | | | 152 (6.48) | | | 15 (4.26) | | | <.0001 | |
| Headache, n (%) | 112 (1.86) | | | 20 (2.48) | | | 32 (1.28) | | | 52 (2.22) | | | 8 (2.27) | | | 0.0404 | |
| Other postprocedural states, n (%) | 93 (1.55) | | | 13 (1.61) | | | 40 (1.60) | | | 34 (1.45) | | | 6 (1.70) | | | 0.9663 | |
| Abnormal results of function, n (%) | 342 (5.69) | | | 56 (6.94) | | | 123 (4.91) | | | 139 (5.93) | | | 24 (6.82) | | | 0.1001 | |
| Unspecified multiple injuries, n (%) | 81 (1.35) | | | 18 (2.23) | | | 25 (1.00) | | | 35 (1.49) | | | 3 (0.85) | | | 0.0451 | |
| Amnestic known physiological, n (%) | 126 (2.10) | | | 22 (2.73) | | | 29 (1.16) | | | 69 (2.94) | | | 6 (1.70) | | | 0.0001 | |
| Presence of other functional implants, n (%) | 109 (1.81) | | | 39 (4.83) | | | 21 (0.84) | | | 39 (1.66) | | | 10 (2.84) | | | <.0001 | |
| **Respiratory diseases** | | | | | | | | | | | | | | | | | |
| Vasomotor and allergic rhinitis, n (%) | 268 (4.46) | | | 30 (3.72) | | | 97 (3.87) | | | 128 (5.46) | | | 13 (3.69) | | | 0.0288 | |
| Bronchitis not specified as acute or chronic, n (%) | 211 (3.51) | | | 49 (6.07) | | | 67 (2.68) | | | 82 (3.50) | | | 13 (3.69) | | | 0.0001 | |
| Chronic rhinitis, nasopharyngitis and pharyngitis, n (%) | 120 (2.00) | | | 11 (1.36) | | | 40 (1.60) | | | 63 (2.69) | | | 6 (1.70) | | | 0.0228 | |
| Chronic sinusitis, n (%) | 69 (1.15) | | | 8 (0.99) | | | 27 (1.08) | | | 32 (1.36) | | | 2 (0.57) | | | 0.5138 | |
| Cough, n (%) | 103 (1.71) | | | 17 (2.11) | | | 33 (1.32) | | | 51 (2.17) | | | 2 (0.57) | | | 0.0325 | |
| Abnormalities of breathing, n (%) | 146 (2.43) | | | 40 (4.96) | | | 36 (1.44) | | | 61 (2.60) | | | 9 (2.56) | | | <.0001 | |
| Asthma, n (%) | 183 (3.05) | | | 33 (4.09) | | | 62 (2.48) | | | 74 (3.16) | | | 14 (3.98) | | | 0.0766 | |
| Other chronic obstructive pulmonary disease, n (%) | 107 (1.78) | | | 31 (3.84) | | | 31 (1.24) | | | 40 (1.71) | | | 5 (1.42) | | | <.0001 | |

| Suppl 10. The total variables of step 1 (continued) | | | | | | |
| --- | --- | --- | --- | --- | --- | --- |
|  | **Overall (n=6,008)** | **Cluster1 (n=807)** | **Cluster2 (n=2,504)** | **Cluster3 (n=2,345)** | **Cluster4 (n=352)** | ***p-value*** |
| **Neurologic disorders** | | | | | | |
| Nerve root and plexus disorders, n (%) | 136 (2.26) | 24 (2.97) | 44 (1.76) | 60 (2.56) | 8 (2.27) | 0.1290 |
| Other degenerative nervous NEC, n (%) | 72 (1.20) | 18 (2.23) | 9 (0.36) | 42 (1.79) | 3 (0.85) | <.0001 |
| Mononeuropathies of upper limb, n (%) | 70 (1.17) | 7 (0.87) | 19 (0.76) | 40 (1.71) | 4 (1.14) | 0.0172 |
| Epilepsy and recurrent seizures, n (%) | 65 (1.08) | 19 (2.35) | 20 (0.8) | 21 (0.90) | 5 (1.42) | 0.0016 |
| **Musculoskeletal diseases** | | | | | | |
| Cervical disc disorders, n (%) | 62 (1.03) | 10 (1.24) | 22 (0.88) | 29 (1.24) | 1 (0.28) | 0.2832 |
| Fracture of rib(s), sternum and thoracic spine, n (%) | 63 (1.05) | 20 (2.48) | 19 (0.76) | 19 (0.81) | 5 (1.42) | 0.0002 |
| Osteoporosis not pathological fracture, n (%) | 288 (4.79) | 74 (9.17) | 83 (3.31) | 109 (4.65) | 22 (6.25) | <.0001 |
| Other Unspecified arthropathy, n (%) | 230 (3.83) | 37 (4.58) | 66 (2.64) | 109 (4.65) | 18 (5.11) | 0.0008 |
| Abdominal and pelvic pain, n (%) | 348 (5.79) | 90 (11.15) | 101 (4.03) | 131 (5.59) | 26 (7.39) | <.0001 |
| Gout, n (%) | 455 (7.57) | 61 (7.56) | 189 (7.55) | 175 (7.46) | 30 (8.52) | 0.9196 |
| Other unspecified osteoarthritis, n (%) | 98 (1.63) | 21 (2.60) | 36 (1.44) | 37 (1.58) | 4 (1.14) | 0.1163 |
| Thoracolumbar disc disease, n (%) | 99 (1.65) | 17 (2.11) | 27 (1.08) | 51 (2.17) | 4 (1.14) | 0.0138 |
| Osteoarthritis of knee, n (%) | 310 (5.16) | 73 (9.05) | 97 (3.87) | 123 (5.25) | 17 (4.83) | <.0001 |
| Other deforming dorsopathies, n (%) | 124 (2.06) | 24 (2.97) | 44 (1.76) | 50 (2.13) | 6 (1.70) | 0.1910 |
| Spondylosis, n (%) | 312 (5.19) | 66 (8.18) | 86 (3.43) | 146 (6.23) | 14 (3.98) | <.0001 |
| Other enthesopathies, n (%) | 126 (2.10) | 24 (2.97) | 39 (1.56) | 48 (2.05) | 15 (4.26) | 0.0022 |
| Fracture of lumbar spine and pelvis, n (%) | 79 (1.31) | 26 (3.22) | 21 (0.84) | 26 (1.11) | 6 (1.70) | <.0001 |
| Suppl 10. The total variables of step 1 (continued) | | | | | | |
|  | **Overall (n=6,008)** | **Cluster1 (n=807)** | **Cluster2 (n=2,504)** | **Cluster3 (n=2,345)** | **Cluster4 (n=352)** | ***p-value*** |
| Shoulder lesions, n (%) | 69 (1.15) | 11 (1.36) | 23 (0.92) | 31 (1.32) | 4 (1.14) | 0.5487 |
| **Renal disorders** | | | | | | |
| Chronic kidney disease (CKD), n (%) | 339 (5.64) | 169 (20.94) | 51 (2.04) | 96 (4.09) | 23 (6.53) | <.0001 |
| Other disease kidney and ureter NEC, n (%) | 100 (1.66) | 34 (4.21) | 12 (0.48) | 45 (1.92) | 9 (2.56) | <.0001 |
| Calculus of kidney and ureter, n (%) | 395 (6.57) | 61 (7.56) | 79 (3.15) | 235 (10.02) | 20 (5.68) | <.0001 |
| Acute kidney failure, n (%) | 73 (1.22) | 48 (5.95) | 4 (0.16) | 18 (0.77) | 3 (0.85) | <.0001 |
| **Bleeding events** | | | | | | |
| Hematuria, n (%) | 259 (4.31) | 42 (5.20) | 13 (0.52) | 186 (7.93) | 18 (5.11) | <.0001 |
| **Urinary tract disease** | | | | | | |
| Other disease urinary system, n (%) | 736 (12.25) | 208 (25.77) | 54 (2.16) | 418 (17.83) | 56 (15.91) | <.0001 |
| Benign prostatic hyperplasia, n (%) | 379 (6.31) | 77 (9.54) | 83 (3.31) | 201 (8.57) | 18 (5.11) | <.0001 |
| Pain micturition, n (%) | 75 (1.25) | 15 (1.86) | 12 (0.48) | 46 (1.96) | 2 (0.57) | <.0001 |
| Polyuria, n (%) | 303 (5.04) | 67 (8.30) | 49 (1.96) | 170 (7.25) | 17 (4.83) | <.0001 |
| Proteinuria, n (%) | 106 (1.76) | 13 (1.61) | 17 (0.68) | 66 (2.81) | 10 (2.84) | <.0001 |
| Obstructive and reflux uropathy, n (%) | 134 (2.23) | 35 (4.34) | 14 (0.56) | 78 (3.33) | 7 (1.99) | <.0001 |
| **Sleep problem** | | | | | | |
| Sleep disorders, n (%) | 414 (6.89) | 72 (8.92) | 117 (4.67) | 203 (8.66) | 22 (6.25) | <.0001 |
| Sleep disorders not substance or physiological, n (%) | 261 (4.34) | 47 (5.82) | 95 (3.79) | 105 (4.48) | 14 (3.98) | 0.0982 |
| **Bacterial infection** | | | | | | |
| Suppl 10. The total variables of step 1 (continued) | | | | | | |
|  | **Overall (n=6,008)** | **Cluster1 (n=807)** | **Cluster2 (n=2,504)** | **Cluster3 (n=2,345)** | **Cluster4 (n=352)** | ***p-value*** |
| Other bacterial cause disease, n (%) | 167 (2.78) | 46 (5.70) | 35 (1.40) | 77 (3.28) | 9 (2.56) | <.0001 |
| Other Sepsis, n (%) | 296 (4.93) | 183 (22.68) | 21 (0.84) | 76 (3.24) | 16 (4.55) | <.0001 |
| Pneumonia unspecified site, n (%) | 202 (3.36) | 102 (12.64) | 25 (1.00) | 62 (2.64) | 13 (3.69) | <.0001 |
| Acute upper respiratory infections of multiple and unspecified sites, n (%) | 133 (2.21) | 15 (1.86) | 51 (2.04) | 62 (2.64) | 5 (1.42) | 0.2770 |
| Cellulitis and acute lymphangitis, n (%) | 122 (2.03) | 34 (4.21) | 31 (1.24) | 50 (2.13) | 7 (1.99) | <.0001 |
| **Skin disorder** | | | | | | |
| Other and unspecified dermatitis, n (%) | 182 (3.03) | 35 (4.34) | 70 (2.80) | 66 (2.81) | 11 (3.13) | 0.1361 |
| Herpes zoster, n (%) | 69 (1.15) | 24 (2.97) | 20 (0.80) | 20 (0.85) | 5 (1.42) | <.0001 |
| Other benign neoplasms of skin, n (%) | 100 (1.66) | 8 (0.99) | 40 (1.60) | 48 (2.05) | 4 (1.14) | 0.1719 |
| Dermatophytosis, n (%) | 104 (1.73) | 22 (2.73) | 30 (1.20) | 43 (1.83) | 9 (2.56) | 0.0152 |
| Urticaria, n (%) | 75 (1.25) | 11 (1.36) | 26 (1.04) | 33 (1.41) | 5 (1.42) | 0.6715 |
| Other disorders of skin and subcutaneous NEC, n (%) | 70 (1.17) | 18 (2.23) | 24 (0.96) | 25 (1.07) | 3 (0.85) | 0.0246 |
| Other Unspecified soft tissue NEC, n (%) | 92 (1.53) | 21 (2.60) | 22 (0.88) | 45 (1.92) | 4 (1.14) | 0.0012 |
| Other systemic connective tissue, n (%) | 99 (1.65) | 21 (2.60) | 26 (1.04) | 47 (2.00) | 5 (1.42) | 0.0066 |
| **Immune diseases** | | | | | | |
| Other disease immune NEC, n (%) | 120 (2.00) | 17 (2.11) | 47 (1.88) | 49 (2.09) | 7 (1.99) | 0.9530 |
| **Psychiatric disorders** | | | | | | |
| Unspecified dementia, n (%) | 94 (1.56) | 35 (4.34) | 24 (0.96) | 31 (1.32) | 4 (1.14) | <.0001 |
| Suppl 10. The total variables of step 1 (continued) | | | | | | |
|  | **Overall (n=6,008)** | **Cluster1 (n=807)** | **Cluster2 (n=2,504)** | **Cluster3 (n=2,345)** | **Cluster4 (n=352)** | ***p-value*** |
| Other anxiety disorders, n (%) | 232 (3.86) | 43 (5.33) | 80 (3.19) | 91 (3.88) | 18 (5.11) | 0.0272 |
| Depressive episode, n (%) | 88 (1.46) | 23 (2.85) | 24 (0.96) | 36 (1.54) | 5 (1.42) | 0.0016 |
| **Behavioural disorders** | | | | | | |
| Nicotine dependence, n (%) | 146 (2.43) | 28 (3.47) | 39 (1.56) | 74 (3.16) | 5 (1.42) | 0.0004 |
| **Diseases of oral cavity and salivary glands** | | | | | | |
| Dental caries, n (%) | 125 (2.08) | 18 (2.23) | 43 (1.72) | 56 (2.39) | 8 (2.27) | 0.4131 |
| Gingivitis and periodontal disease, n (%) | 392 (6.52) | 72 (8.92) | 151 (6.03) | 145 (6.18) | 24 (6.82) | 0.0279 |
| Stomatitis and related lesions, n (%) | 175 (2.91) | 35 (4.34) | 61 (2.44) | 68 (2.90) | 11 (3.13) | 0.0490 |
| **Gynecological diseases** | | | | | | |
| Unspecified lump in breast, n (%) | 96 (1.60) | 11 (1.36) | 37 (1.48) | 39 (1.66) | 9 (2.56) | 0.4513 |
| Menopausal and other perimenopausal disorders, n (%) | 80 (1.33) | 20 (2.48) | 23 (0.92) | 33 (1.41) | 4 (1.14) | 0.0092 |
| **Eye disorders** | | | | | | |
| Disorders of lacrimal system, n (%) | 179 (2.98) | 40 (4.96) | 43 (1.72) | 81 (3.45) | 15 (4.26) | <.0001 |
| Glaucoma, n (%) | 94 (1.56) | 20 (2.48) | 23 (0.92) | 43 (1.83) | 8 (2.27) | 0.0038 |
| Conjunctivitis, n (%) | 127 (2.11) | 28 (3.47) | 28 (1.12) | 64 (2.73) | 7 (1.99) | <.0001 |
| Visual disturbances, n (%) | 118 (1.96) | 20 (2.48) | 39 (1.56) | 49 (2.09) | 10 (2.84) | 0.1827 |
| Age related cataract, n (%) | 207 (3.45) | 53 (6.57) | 52 (2.08) | 89 (3.80) | 13 (3.69) | <.0001 |
| Other retinal disorders, n (%) | 125 (2.08) | 27 (3.35) | 32 (1.28) | 55 (2.35) | 11 (3.13) | 0.0007 |
| **Ear disorders** | | | | | | |
| Suppl 10. The total variables of step 1 (continued) | | | | | | |
|  | **Overall (n=6,008)** | **Cluster1 (n=807)** | **Cluster2 (n=2,504)** | **Cluster3 (n=2,345)** | **Cluster4 (n=352)** | ***p-value*** |
| Other Unspecified hearing loss, n (%) | 87 (1.45) | 11 (1.36) | 24 (0.96) | 42 (1.79) | 10 (2.84) | 0.0119 |
| Benign neoplasm of middle ear and respiratory system, n (%) | 65 (1.08) | 11 (1.36) | 27 (1.08) | 22 (0.94) | 5 (1.42) | 0.6994 |
| Other disorders ear NEC, n (%) | 75 (1.25) | 6 (0.74) | 32 (1.28) | 32 (1.36) | 5 (1.42) | 0.5666 |
| **Malignancy** | | | | | | |
| Abnormal tumor markers, n (%) | 70 (1.17) | 12 (1.49) | 20 (0.80) | 33 (1.41) | 5 (1.42) | 0.1690 |
| Neoplasm oral cavity and digestive, n (%) | 454 (7.56) | 36 (4.46) | 241 (9.62) | 142 (6.06) | 35 (9.94) | <.0001 |
| Breast cancer, n (%) | 118 (1.96) | 18 (2.23) | 57 (2.28) | 39 (1.66) | 4 (1.14) | 0.2701 |
| Neoplasms of unspecified behavior, n (%) | 127 (2.11) | 30 (3.72) | 51 (2.04) | 38 (1.62) | 8 (2.27) | 0.0049 |

Suppl 10. The total variables of step 1 (continued)

|  | **Overall (n=6,008)** | **Cluster1 (n=807)** | **Cluster2 (n=2,504)** | **Cluster3 (n=2,345)** | **Cluster4 (n=352)** | ***p-value*** |
| --- | --- | --- | --- | --- | --- | --- |
| **Medical intervention history** | | | | | | |
| Cholecystectomy, n(%) | 157 (2.61) | 36 (4.46) | 69 (2.76) | 47 (2.00) | 5 (1.42) | 0.0009 |
| Cardiac catheterization, n(%) | 130 (2.16) | 31 (3.84) | 24 (0.96) | 68 (2.90) | 7 (1.99) | <.0001 |
| Coronary arteriography, n(%) | 128 (2.13) | 30 (3.72) | 23 (0.92) | 68 (2.90) | 7 (1.99) | <.0001 |
| Upper gastrointestinal endoscopy, n(%) | 793 (13.20) | 122 (15.12) | 105 (4.19) | 514 (21.92) | 52 (14.77) | <.0001 |
| Lower gastrointestinal endoscopy, n(%) | 372 (6.19) | 48 (5.95) | 51 (2.04) | 239 (10.19) | 34 (9.66) | <.0001 |
| CT scan abdomen, n(%) | 135 (2.25) | 55 (6.82) | 29 (1.16) | 42 (1.79) | 9 (2.56) | <.0001 |
| Partial skin resection, n(%) | 67 (1.12) | 13 (1.61) | 21 (0.84) | 30 (1.28) | 3 (0.85) | 0.2281 |
| Injection of neoplastic drug, n(%) | 87 (1.45) | 54 (6.69) | 7 (0.28) | 22 (0.94) | 4 (1.14) | <.0001 |
| MRI of head, n(%) | 94 (1.56) | 16 (1.98) | 12 (0.48) | 58 (2.47) | 8 (2.27) | <.0001 |
| Resection lymphatic, n(%) | 63 (1.05) | 24 (2.97) | 21 (0.84) | 17 (0.72) | 1 (0.28) | <.0001 |
| Check month and throat, n(%) | 122 (2.03) | 19 (2.35) | 38 (1.52) | 57 (2.43) | 8 (2.27) | 0.1256 |

Suppl 10. The total variables of step 1 (continued)

|  | **Overall (n=6,008)** | **Cluster1 (n=807)** | **Cluster2 (n=2,504)** | **Cluster3 (n=2,345)** | **Cluster4 (n=352)** | ***p-value*** |
| --- | --- | --- | --- | --- | --- | --- |
| **Antiulcer drugs** | | | | | | |
| Dimethicone | 1299 (21.62) | 296 (36.68) | 293 (11.70) | 631 (26.91) | 79 (22.44) | <.0001 |
| **H2 receptor antagonists** | | | | | | |
| Ranitidine, n (%) | 962 (16.01) | 207 (25.65) | 282 (11.26) | 409 (17.44) | 64 (18.18) | <.0001 |
| Famotidine, n (%) | 566 (9.42) | 140 (17.35) | 162 (6.47) | 226 (9.64) | 38 (10.80) | <.0001 |
| **Antacids** | | | | | | |
| Alginic acid, n (%) | 777 (12.93) | 107 (13.26) | 158 (6.31) | 455 (19.40) | 57 (16.19) | <.0001 |
| **Proton pump inhibitors** | | | | | | |
| Dexlansoprazole, n (%) | 545 (9.07) | 109 (13.51) | 146 (5.83) | 256 (10.92) | 34 (9.66) | <.0001 |
| Rabeprazole, n (%) | 632 (10.52) | 85 (10.53) | 64 (2.56) | 441 (18.81) | 42 (11.93) | <.0001 |
| Esomeprazole, n (%) | 184 (3.06) | 57 (7.06) | 62 (2.48) | 50 (2.13) | 15 (4.26) | <.0001 |
| Pantoprazole, n (%) | 147 (2.45) | 86 (10.66) | 21 (0.84) | 33 (1.41) | 7 (1.99) | <.0001 |
| **ARB** | | | | | | |
| Valsartan, n (%) | 1205 (20.06) | 257 (31.85) | 395 (15.77) | 480 (20.47) | 73 (20.74) | <.0001 |
| Azilsartan, n (%) | 281 (4.68) | 59 (7.31) | 101 (4.03) | 107 (4.56) | 14 (3.98) | 0.0015 |
| Olmesartan, n (%) | 233 (3.88) | 58 (7.19) | 77 (3.08) | 91 (3.88) | 7 (1.99) | <.0001 |
| **ACEI** | | | | | | |
| Captopril, n (%) | 380 (6.32) | 137 (16.98) | 94 (3.75) | 122 (5.20) | 27 (7.67) | <.0001 |
| Benazepril, n (%) | 74 (1.23) | 6 (0.74) | 30 (1.20) | 32 (1.36) | 6 (1.70) | 0.4589 |

| Suppl 10. The total variables of step 1 (continued) | | | | | | |
| --- | --- | --- | --- | --- | --- | --- |
|  | **Overall (n=6,008)** | **Cluster1 (n=807)** | **Cluster2 (n=2,504)** | **Cluster3 (n=2,345)** | **Cluster4 (n=352)** | ***p-value*** |
| **CCB** | | | | | | |
| Amlodipine, n (%) | 1556 (25.90) | 342 (42.38) | 525 (20.97) | 598 (25.50) | 91 (25.85) | <.0001 |
| Nifedipine, n (%) | 241 (4.01) | 86 (10.66) | 59 (2.36) | 80 (3.41) | 16 (4.55) | <.0001 |
| Diltiazem, n (%) | 119 (1.98) | 39 (4.83) | 39 (1.56) | 34 (1.45) | 7 (1.99) | <.0001 |
| Lercanidipine, n (%) | 134 (2.23) | 32 (3.97) | 51 (2.04) | 41 (1.75) | 10 (2.84) | 0.0021 |
| **Alpha and beta-blockers** | | | | | | |
| Carvedilol , n (%) | 138 (2.30) | 41 (5.08) | 45 (1.80) | 43 (1.83) | 9 (2.56) | <.0001 |
| Labetalol, n (%) | 65 (1.08) | 27 (3.35) | 15 (0.60) | 16 (0.68) | 7 (1.99) | <.0001 |
| **Beta-blockers** | | | | | | |
| Bisoprolol, n (%) | 735 (12.23) | 170 (21.07) | 255 (10.18) | 269 (11.47) | 41 (11.65) | <.0001 |
| Propranolol, n (%) | 361 (6.01) | 59 (7.31) | 124 (4.95) | 154 (6.57) | 24 (6.82) | 0.0283 |
| Nebivolol, n (%) | 95 (1.58) | 17 (2.11) | 42 (1.68) | 29 (1.24) | 7 (1.99) | 0.2905 |
| **Alpha1-blockers** | | | | | | |
| Tamsulosin, n (%) | 321 (5.34) | 90 (11.15) | 57 (2.28) | 162 (6.91) | 12 (3.41) | <.0001 |
| Doxazosin, n (%) | 135 (2.25) | 48 (5.95) | 32 (1.28) | 46 (1.96) | 9 (2.56) | <.0001 |
| Silodosin, n (%) | 104 (1.73) | 30 (3.72) | 16 (0.64) | 55 (2.35) | 3 (0.85) | <.0001 |
| **Nitrates** | | | | | | |
| Isosorbide mononitrate, n (%) | 153 (2.55) | 58 (7.19) | 26 (1.04) | 59 (2.52) | 10 (2.84) | <.0001 |
| Nitroglycerin, n (%) | 256 (4.26) | 78 (9.67) | 74 (2.96) | 90 (3.84) | 14 (3.98) | <.0001 |
| Suppl 10. The total variables of step 1 (continued) | | | | | | |
|  | **Overall (n=6,008)** | **Cluster1 (n=807)** | **Cluster2 (n=2,504)** | **Cluster3 (n=2,345)** | **Cluster4 (n=352)** | ***p-value*** |
| **Vasodilators** | | | | | | |
| Nicorandil, n (%) | 108 (1.80) | 38 (4.71) | 34 (1.36) | 33 (1.41) | 3 (0.85) | <.0001 |
| **Diuretics** | | | | | | |
| Furosemide , n (%) | 266 (4.43) | 143 (17.72) | 42 (1.68) | 68 (2.90) | 13 (3.69) | <.0001 |
| Spironolactone, n (%) | 125 (2.08) | 57 (7.06) | 25 (1.00) | 34 (1.45) | 9 (2.56) | <.0001 |
| Hydrochlorothiazide, n (%) | 319 (5.31) | 71 (8.80) | 120 (4.79) | 108 (4.61) | 20 (5.68) | <.0001 |
| Indapamide, n (%) | 108 (1.80) | 19 (2.35) | 40 (1.60) | 42 (1.79) | 7 (1.99) | 0.5603 |
| **Antiplatelet agents** | | | | | | |
| Dipyridamole , n (%) | 248 (4.13) | 82 (10.16) | 41 (1.64) | 109 (4.65) | 16 (4.55) | <.0001 |
| Aspirin, n (%) | 805 (13.40) | 205 (25.40) | 215 (8.59) | 339 (14.46) | 46 (13.07) | <.0001 |
| Clopidogrel, n (%) | 218 (3.63) | 66 (8.18) | 56 (2.24) | 85 (3.62) | 11 (3.13) | <.0001 |
| Ticagrelor, n (%) | 63 (1.05) | 16 (1.98) | 16 (0.64) | 28 (1.19) | 3 (0.85) | 0.0096 |
| **Anticoagulants** | | | | | | |
| Heparin, n (%) | 242 (4.03) | 93 (11.52) | 39 (1.56) | 96 (4.09) | 14 (3.98) | <.0001 |
| Heparinoid, n (%) | 70 (1.17) | 22 (2.73) | 20 (0.80) | 25 (1.07) | 3 (0.85) | 0.0001 |
| Rivaroxaban, n (%) | 89 (1.48) | 35 (4.34) | 25 (1.00) | 24 (1.02) | 5 (1.42) | <.0001 |
| **Antiarrhythmic drugs** | | | | | | |
| Amiodarone, n (%) | 85 (1.41) | 36 (4.46) | 21 (0.84) | 26 (1.11) | 2 (0.57) | <.0001 |

Suppl 10. The total variables of step 1 (continued)

|  | **Overall (n=6,008)** | **Cluster1 (n=807)** | **Cluster2 (n=2,504)** | **Cluster3 (n=2,345)** | **Cluster4 (n=352)** | ***p-value*** |
| --- | --- | --- | --- | --- | --- | --- |
| **Lipid-lowering drugs** | | | | | | |
| **Statins** | | | | | | |
| Atorvastatin, n (%) | 1254 (20.87) | 185 (22.92) | 437 (17.45) | 569 (24.26) | 63 (17.90) | <.0001 |
| Rosuvastatin, n (%) | 831 (13.83) | 143 (17.72) | 284 (11.34) | 374 (15.95) | 30 (8.52) | <.0001 |
| Fluvastatin, n (%) | 107 (1.78) | 15 (1.86) | 37 (1.48) | 52 (2.22) | 3 (0.85) | 0.1308 |
| Simvastatin, n (%) | 135 (2.25) | 15 (1.86) | 57 (2.28) | 54 (2.30) | 9 (2.56) | 0.8612 |
| Lovastatin, n (%) | 141 (2.35) | 24 (2.97) | 29 (1.16) | 81 (3.45) | 7 (1.99) | <.0001 |
| Pravastatin, n (%) | 76 (1.26) | 9 (1.12) | 34 (1.36) | 29 (1.24) | 4 (1.14) | 0.9445 |
| **Other medications** |  |  |  |  |  |  |
| Ezetimibe, n (%) | 352 (5.86) | 51 (6.32) | 141 (5.63) | 141 (6.01) | 19 (5.40) | 0.8535 |
| Fenofibrate, n (%) | 422 (7.02) | 61 (7.56) | 145 (5.79) | 186 (7.93) | 30 (8.52) | 0.0158 |
| Niacin, n (%) | 141 (2.35) | 24 (2.97) | 29 (1.16) | 81 (3.45) | 7 (1.99) | <.0001 |
| **Hypoglycemic agents** | | | | | | |
| **Sulfonylurea** | | | | | | |
| Gliclazide, n (%) | 287 (4.78) | 96 (11.90) | 49 (1.96) | 123 (5.25) | 19 (5.40) | <.0001 |
| Glimepiride, n (%) | 263 (4.38) | 61 (7.56) | 73 (2.92) | 115 (4.90) | 14 (3.98) | <.0001 |
| **DPP-4 inhibitors** | | | | | | |
| Sitagliptin, n (%) | 273 (4.54) | 68 (8.43) | 75 (3.00) | 118 (5.03) | 12 (3.41) | <.0001 |

Suppl 10. The total variables of step 1 (continued)

|  | **Overall (n=6,008)** | **Cluster1 (n=807)** | **Cluster2 (n=2,504)** | **Cluster3 (n=2,345)** | **Cluster4 (n=352)** | ***p-value*** |
| --- | --- | --- | --- | --- | --- | --- |
| Linagliptin, n (%) | 246 (4.09) | 100 (12.39) | 38 (1.52) | 91 (3.88) | 17 (4.83) | <.0001 |
| Vildagliptin, n (%) | 412 (6.86) | 105 (13.01) | 82 (3.27) | 195 (8.32) | 30 (8.52) | <.0001 |
| **SGLT-2 inhibitors** | | | | | | |
| Dapagliflozin, n (%) | 198 (3.30) | 34 (4.21) | 60 (2.40) | 90 (3.84) | 14 (3.98) | 0.0109 |
| Empagliflozin, n (%) | 143 (2.38) | 31 (3.84) | 24 (0.96) | 73 (3.11) | 15 (4.26) | <.0001 |
| **Insulin** | | | | | | |
| Insulin aspart, n (%) | 192 (3.2) | 89 (11.03) | 16 (0.64) | 69 (2.94) | 18 (5.11) | <.0001 |
| Insulin glargine , n (%) | 177 (2.95) | 76 (9.42) | 19 (0.76) | 64 (2.73) | 18 (5.11) | <.0001 |
| Insulin Human , n (%) | 235 (3.91) | 126 (15.61) | 23 (0.92) | 66 (2.81) | 20 (5.68) | <.0001 |
| **Other medications** |  |  |  |  |  |  |
| Metformin, n (%) | 1303 (21.69) | 277 (34.32) | 359 (14.34) | 588 (25.07) | 79 (22.44) | <.0001 |
| Repaglinide, n (%) | 76 (1.26) | 38 (4.71) | 13 (0.52) | 21 (0.90) | 4 (1.14) | <.0001 |
| Pioglitazone, n (%) | 313 (5.21) | 94 (11.65) | 70 (2.80) | 135 (5.76) | 14 (3.98) | <.0001 |
| Acarbose, n (%) | 128 (2.13) | 47 (5.82) | 22 (0.88) | 50 (2.13) | 9 (2.56) | <.0001 |
| **Thyroid medication** | | | | | | |
| Levothyroxine, n (%) | 230 (3.83) | 51 (6.32) | 71 (2.84) | 94 (4.01) | 14 (3.98) | 0.0001 |
| **Vitamin** | | | | | | |
| Riboflavin, n (%) | 624 (10.39) | 150 (18.59) | 175 (6.99) | 259 (11.04) | 40 (11.36) | <.0001 |

Suppl 10. The total variables of step 1 (continued)

|  | **Overall (n=6,008)** | **Cluster1 (n=807)** | **Cluster2 (n=2,504)** | **Cluster3 (n=2,345)** | **Cluster4 (n=352)** | ***p-value*** |
| --- | --- | --- | --- | --- | --- | --- |
| Thiamine, n (%) | 655 (10.90) | 162 (20.07) | 180 (7.19) | 270 (11.51) | 43 (12.22) | <.0001 |
| Vitamin A, n (%) | 261 (4.34) | 76 (9.42) | 73 (2.92) | 95 (4.05) | 17 (4.83) | <.0001 |
| Vitamin D3, n (%) | 243 (4.04) | 65 (8.05) | 71 (2.84) | 91 (3.88) | 16 (4.55) | <.0001 |
| Pyridoxine, n (%) | 88 (1.46) | 52 (6.44) | 7 (0.28) | 21 (0.90) | 8 (2.27) | <.0001 |
| Folic Acid, n (%) | 109 (1.81) | 45 (5.58) | 20 (0.80) | 36 (1.54) | 8 (2.27) | <.0001 |
| Mecobalamin, n (%) | 238 (3.96) | 67 (8.30) | 40 (1.60) | 111 (4.73) | 20 (5.68) | <.0001 |
| **Other medications** | | | | | | |
| Silymarin, n (%) | 1498 (24.93) | 222 (27.51) | 593 (23.68) | 595 (25.37) | 88 (25.00) | 0.1578 |
| Strocain, n (%) | 1659 (27.61) | 317 (39.28) | 424 (16.93) | 802 (34.20) | 116 (32.95) | <.0001 |
| Flopropione, n (%) | 185 (3.08) | 50 (6.20) | 68 (2.72) | 61 (2.60) | 6 (1.70) | <.0001 |
| Acetaminophen, n (%) | 2359 (39.26) | 576 (71.38) | 678 (27.08) | 952 (40.60) | 153 (43.47) | <.0001 |
| Dextrose Monohydrate , n (%) | 262 (4.36) | 109 (13.51) | 47 (1.88) | 89 (3.80) | 17 (4.83) | <.0001 |
| Sodium Chloride, n (%) | 1923 (32.01) | 583 (72.24) | 472 (18.85) | 741 (31.60) | 127 (36.08) | <.0001 |
| Sodium lactate, n (%) | 402 (6.69) | 140 (17.35) | 107 (4.27) | 137 (5.84) | 18 (5.11) | <.0001 |
| Pseudoephedrine, n (%) | 145 (2.41) | 18 (2.23) | 54 (2.16) | 65 (2.77) | 8 (2.27) | 0.5469 |
| Glucose, n (%) | 524 (8.72) | 196 (24.29) | 122 (4.87) | 173 (7.38) | 33 (9.38) | <.0001 |
| Lysozyme, n (%) | 645 (10.74) | 168 (20.82) | 172 (6.87) | 266 (11.34) | 39 (11.08) | <.0001 |
| Ursodeoxycholic acid, n (%) | 581 (9.67) | 88 (10.90) | 249 (9.94) | 202 (8.61) | 42 (11.93) | 0.0829 |

Suppl 10. The total variables of step 1 (continued)

|  | **Overall (n=6,008)** | **Cluster1 (n=807)** | **Cluster2 (n=2,504)** | **Cluster3 (n=2,345)** | **Cluster4 (n=352)** | ***p-value*** |
| --- | --- | --- | --- | --- | --- | --- |
| Lidocaine, n (%) | 651 (10.84) | 170 (21.07) | 191 (7.63) | 246 (10.49) | 44 (12.50) | <.0001 |
| Epinephrine, n (%) | 104 (1.73) | 38 (4.71) | 19 (0.76) | 41 (1.75) | 6 (1.70) | <.0001 |
| Magnesium Oxide , n (%) | 514 (8.56) | 139 (17.22) | 125 (4.99) | 216 (9.21) | 34 (9.66) | <.0001 |
| Propofol , n (%) | 68 (1.13) | 24 (2.97) | 18 (0.72) | 23 (0.98) | 3 (0.85) | <.0001 |
| Tears Naturale, n (%) | 160 (2.66) | 40 (4.96) | 45 (1.80) | 65 (2.77) | 10 (2.84) | <.0001 |
| Urea, n (%) | 104 (1.73) | 31 (3.84) | 30 (1.20) | 39 (1.66) | 4 (1.14) | <.0001 |
| Zinc Oxide, n (%) | 73 (1.22) | 20 (2.48) | 17 (0.68) | 29 (1.24) | 7 (1.99) | 0.0003 |
| Cysteine , n (%) | 66 (1.10) | 18 (2.23) | 27 (1.08) | 16 (0.68) | 5 (1.42) | 0.0035 |
| Tranexamic acid, n (%) | 509 (8.47) | 151 (18.71) | 120 (4.79) | 206 (8.78) | 32 (9.09) | <.0001 |
| Tetanus toxoid , n (%) | 99 (1.65) | 14 (1.73) | 29 (1.16) | 43 (1.83) | 13 (3.69) | 0.0040 |
| Glucosamine, n (%) | 74 (1.23) | 10 (1.24) | 21 (0.84) | 40 (1.71) | 3 (0.85) | 0.0475 |
| Influenza vaccine, n (%) | 958 (15.95) | 140 (17.35) | 315 (12.58) | 434 (18.51) | 69 (19.60) | <.0001 |
| Antimony potassium tartrate , n (%) | 254 (4.23) | 70 (8.67) | 63 (2.52) | 103 (4.39) | 18 (5.11) | <.0001 |
| Camphorated opium tincture, n (%) | 254 (4.23) | 70 (8.67) | 63 (2.52) | 103 (4.39) | 18 (5.11) | <.0001 |
| Glycyrrhiza Extract, n (%) | 254 (4.23) | 70 (8.67) | 63 (2.52) | 103 (4.39) | 18 (5.11) | <.0001 |
| Carbomer, n (%) | 82 (1.36) | 18 (2.23) | 20 (0.80) | 39 (1.66) | 5 (1.42) | 0.0073 |
| Piracetam, n (%) | 95 (1.58) | 22 (2.73) | 23 (0.92) | 41 (1.75) | 9 (2.56) | 0.0009 |
| Pentoxifylline, n (%) | 101 (1.68) | 33 (4.09) | 26 (1.04) | 37 (1.58) | 5 (1.42) | <.0001 |

Suppl 10. The total variables of step 1 (continued)

|  | **Overall (n=6,008)** | **Cluster1 (n=807)** | **Cluster2 (n=2,504)** | **Cluster3 (n=2,345)** | **Cluster4 (n=352)** | ***p-value*** |
| --- | --- | --- | --- | --- | --- | --- |
| Hydroxychloroquine, n (%) | 61 (1.02) | 19 (2.35) | 16 (0.64) | 22 (0.94) | 4 (1.14) | 0.0004 |
| Fructose, n (%) | 66 (1.10) | 39 (4.83) | 5 (0.20) | 17 (0.72) | 5 (1.42) | <.0001 |
| Pirenoxine, n (%) | 115 (1.91) | 30 (3.72) | 31 (1.24) | 49 (2.09) | 5 (1.42) | 0.0001 |
| Levetiracetam, n (%) | 61 (1.02) | 27 (3.35) | 14 (0.56) | 16 (0.68) | 4 (1.14) | <.0001 |
| Montelukast, n (%) | 62 (1.03) | 11 (1.36) | 17 (0.68) | 27 (1.15) | 7 (1.99) | 0.0601 |
| **Anti_dizziness_agent** | | | | | | |
| Flunarizine, n (%) | 239 (3.98) | 34 (4.21) | 82 (3.27) | 105 (4.48) | 18 (5.11) | 0.1078 |
| Diphenidol, n (%) | 479 (7.97) | 94 (11.65) | 143 (5.71) | 204 (8.70) | 38 (10.80) | <.0001 |
| **Medication for mental problems** | | | | | | |
| Alprazolam, n (%) | 548 (9.12) | 128 (15.86) | 149 (5.95) | 229 (9.77) | 42 (11.93) | <.0001 |
| Quetiapine, n (%) | 76 (1.26) | 34 (4.21) | 11 (0.44) | 23 (0.98) | 8 (2.27) | <.0001 |
| Trazodone, n (%) | 133 (2.21) | 41 (5.08) | 43 (1.72) | 40 (1.71) | 9 (2.56) | <.0001 |
| Imipramine, n (%) | 76 (1.26) | 8 (0.99) | 27 (1.08) | 32 (1.36) | 9 (2.56) | 0.1081 |
| Pregabalin, n (%) | 72 (1.20) | 28 (3.47) | 16 (0.64) | 23 (0.98) | 5 (1.42) | <.0001 |
| **Ion association drug** | | | | | | |
| Calcium phosphate tribasic, n (%) | 236 (3.93) | 64 (7.93) | 70 (2.80) | 86 (3.67) | 16 (4.55) | <.0001 |
| Calcium chloride , n (%) | 150 (2.50) | 88 (10.90) | 21 (0.84) | 34 (1.45) | 7 (1.99) | <.0001 |

Suppl 10. The total variables of step 1 (continued)

|  | **Overall (n=6,008)** | **Cluster1 (n=807)** | **Cluster2 (n=2,504)** | **Cluster3 (n=2,345)** | **Cluster4 (n=352)** | ***p-value*** |
| --- | --- | --- | --- | --- | --- | --- |
| Calcium Bromide, n (%) | 71 (1.18) | 46 (5.70) | 2 (0.08) | 16 (0.68) | 7 (1.99) | <.0001 |
| Sodium Phosphate, Dibasic, Anhydrous, n (%) | 171 (2.85) | 71 (8.80) | 46 (1.84) | 49 (2.09) | 5 (1.42) | <.0001 |
| Iron, n (%) | 60 (1.00) | 21 (2.60) | 5 (0.20) | 9 (0.38) | 25 (7.10) | <.0001 |
| Potassium Chloride, n (%) | 449 (7.47) | 198 (24.54) | 65 (2.60) | 160 (6.82) | 26 (7.39) | <.0001 |
| Potassium Gluconate , n (%) | 311 (5.18) | 150 (18.59) | 38 (1.52) | 104 (4.43) | 19 (5.40) | <.0001 |
| Sodium Bicarbonate, n (%) | 95 (1.58) | 60 (7.43) | 11 (0.44) | 19 (0.81) | 5 (1.42) | <.0001 |
| Calcium chloride dihydrate, n (%) | 164 (2.73) | 68 (8.43) | 30 (1.20) | 60 (2.56) | 6 (1.70) | <.0001 |
| Magnesium Sulfate, n (%) | 67 (1.12) | 50 (6.20) | 4 (0.16) | 9 (0.38) | 4 (1.14) | <.0001 |
| Iodine , n (%) | 79 (1.31) | 29 (3.59) | 14 (0.56) | 29 (1.24) | 7 (1.99) | <.0001 |
| **Opioids** | | | | | | |
| Loperamide, n (%) | 106 (1.76) | 47 (5.82) | 12 (0.48) | 41 (1.75) | 6 (1.70) | <.0001 |
| Meperidine , n (%) | 131 (2.18) | 53 (6.57) | 32 (1.28) | 41 (1.75) | 5 (1.42) | <.0001 |
| Tramadol , n (%) | 688 (11.45) | 257 (31.85) | 161 (6.43) | 224 (9.55) | 46 (13.07) | <.0001 |
| Morphine, n (%) | 337 (5.61) | 115 (14.25) | 94 (3.75) | 111 (4.73) | 17 (4.83) | <.0001 |
| Fentanyl, n (%) | 137 (2.28) | 69 (8.55) | 25 (1.00) | 36 (1.54) | 7 (1.99) | <.0001 |
| **Intestinal related drugs** | | | | | | |
| Otilonium bromide, n (%) | 353 (5.88) | 53 (6.57) | 44 (1.76) | 229 (9.77) | 27 (7.67) | <.0001 |
| Pinaverium, n (%) | 118 (1.96) | 15 (1.86) | 32 (1.28) | 61 (2.60) | 10 (2.84) | 0.0058 |

Suppl 10. The total variables of step 1 (continued)

|  | **Overall (n=6,008)** | **Cluster1 (n=807)** | **Cluster2 (n=2,504)** | **Cluster3 (n=2,345)** | **Cluster4 (n=352)** | ***p-value*** |
| --- | --- | --- | --- | --- | --- | --- |
| Smecta, n (%) | 363 (6.04) | 138 (17.10) | 48 (1.92) | 157 (6.70) | 20 (5.68) | <.0001 |
| Sodium phosphate monobasic, n (%) | 171 (2.85) | 71 (8.80) | 46 (1.84) | 49 (2.09) | 5 (1.42) | <.0001 |
| Bisacodyl, n (%) | 390 (6.49) | 183 (22.68) | 41 (1.64) | 146 (6.23) | 20 (5.68) | <.0001 |
| Sennoside, n (%) | 974 (16.21) | 328 (40.64) | 176 (7.03) | 407 (17.36) | 63 (17.90) | <.0001 |
| Lactulose , n (%) | 163 (2.71) | 80 (9.91) | 17 (0.68) | 59 (2.52) | 7 (1.99) | <.0001 |
| **Prokinetic agents** | | | | | | |
| Mosapride, n (%) | 752 (12.52) | 135 (16.73) | 124 (4.95) | 434 (18.51) | 59 (16.76) | <.0001 |
| Metoclopramide, n (%) | 529 (8.80) | 206 (25.53) | 120 (4.79) | 168 (7.16) | 35 (9.94) | <.0001 |
| Domperidone, n (%) | 282 (4.69) | 90 (11.15) | 78 (3.12) | 100 (4.26) | 14 (3.98) | <.0001 |
| **Urinary tract related drugs** | | | | | | |
| Solifenacin, n (%) | 88 (1.46) | 28 (3.47) | 15 (0.60) | 43 (1.83) | 2 (0.57) | <.0001 |
| Dutasteride, n (%) | 73 (1.22) | 18 (2.23) | 14 (0.56) | 38 (1.62) | 3 (0.85) | 0.0002 |
| Mirabegron, n (%) | 110 (1.83) | 27 (3.35) | 18 (0.72) | 63 (2.69) | 2 (0.57) | <.0001 |
| Phenazopyridine, n (%) | 133 (2.21) | 34 (4.21) | 9 (0.36) | 84 (3.58) | 6 (1.70) | <.0001 |
| **Musculoskeletal related drugs** | | | | | | |
| Chlorzoxazone, n (%) | 313 (5.21) | 59 (7.31) | 99 (3.95) | 138 (5.88) | 17 (4.83) | 0.0006 |
| **Anticholinergic agents** | | | | | | |
| Scopolamin, n (%) | 1016 (16.91) | 176 (21.81) | 232 (9.27) | 542 (23.11) | 66 (18.75) | <.0001 |

Suppl 10. The total variables of step 1 (continued)

|  | **Overall (n=6,008)** | **Cluster1 (n=807)** | **Cluster2 (n=2,504)** | **Cluster3 (n=2,345)** | **Cluster4 (n=352)** | ***p-value*** |
| --- | --- | --- | --- | --- | --- | --- |
| Mepenzolate, n (%) | 166 (2.76) | 43 (5.33) | 23 (0.92) | 88 (3.75) | 12 (3.41) | <.0001 |
| **First generation Antihistamines** | | | | | | |
| Diphenhydramine, n (%) | 566 (9.42) | 204 (25.28) | 134 (5.35) | 189 (8.06) | 39 (11.08) | <.0001 |
| Homochlorcyclizine, n (%) | 197 (3.28) | 55 (6.82) | 50 (2.00) | 78 (3.33) | 14 (3.98) | <.0001 |
| Ketotifen , n (%) | 139 (2.31) | 25 (3.10) | 48 (1.92) | 55 (2.35) | 11 (3.13) | 0.1736 |
| **Second generation Antihistamines** | | | | | | |
| Loratadine, n (%) | 352 (5.86) | 67 (8.30) | 111 (4.43) | 153 (6.52) | 21 (5.97) | 0.0002 |
| Levocetirizine, n (%) | 505 (8.41) | 101 (12.52) | 154 (6.15) | 216 (9.21) | 34 (9.66) | <.0001 |
| Epinastine , n (%) | 62 (1.03) | 17 (2.11) | 16 (0.64) | 26 (1.11) | 3 (0.85) | 0.0043 |
| **Mucolytic agents** | | | | | | |
| Acetylcysteine, n (%) | 517 (8.61) | 187 (23.17) | 135 (5.39) | 165 (7.04) | 30 (8.52) | <.0001 |
| Ambroxol, n (%) | 553 (9.20) | 161 (19.95) | 147 (5.87) | 210 (8.96) | 35 (9.94) | <.0001 |
| Potassium Cresolsulfonate, n (%) | 645 (10.74) | 168 (20.82) | 172 (6.87) | 266 (11.34) | 39 (11.08) | <.0001 |
| **Cough medicine** | | | | | | |
| Dextromethorphan, n (%) | 649 (10.8) | 169 (20.94) | 172 (6.87) | 268 (11.43) | 40 (11.36) | <.0001 |
| **Benzodiazepines** | | | | | | |
| Brotizolam, n (%) | 113 (1.88) | 24 (2.97) | 33 (1.32) | 42 (1.79) | 14 (3.98) | 0.0004 |
| Estazolam, n (%) | 227 (3.78) | 64 (7.93) | 56 (2.24) | 87 (3.71) | 20 (5.68) | <.0001 |

Suppl 10. The total variables of step 1 (continued)

|  | **Overall (n=6,008)** | **Cluster1 (n=807)** | **Cluster2 (n=2,504)** | **Cluster3 (n=2,345)** | **Cluster4 (n=352)** | ***p-value*** |
| --- | --- | --- | --- | --- | --- | --- |
| Lorazepam, n (%) | 359 (5.98) | 122 (15.12) | 93 (3.71) | 122 (5.20) | 22 (6.25) | <.0001 |
| Bromazepam, n (%) | 76 (1.26) | 15 (1.86) | 23 (0.92) | 29 (1.24) | 9 (2.56) | 0.0244 |
| Diazepam , n (%) | 92 (1.53) | 17 (2.11) | 34 (1.36) | 31 (1.32) | 10 (2.84) | 0.0733 |
| Zolpidem, n (%) | 176 (2.93) | 45 (5.58) | 52 (2.08) | 68 (2.9) | 11 (3.13) | <.0001 |
| Clonazepam, n (%) | 248 (4.13) | 61 (7.56) | 67 (2.68) | 102 (4.35) | 18 (5.11) | <.0001 |
| **Antibiotics** |  |  |  |  |  |  |
| **Penicillins** |  |  |  |  |  |  |
| Amoxicillin, n (%) | 461 (7.67) | 125 (15.49) | 120 (4.79) | 183 (7.80) | 33 (9.38) | <.0001 |
| Ampicillin, n (%) | 62 (1.03) | 25 (3.10) | 13 (0.52) | 18 (0.77) | 6 (1.70) | <.0001 |
| Sultamicillin, n (%) | 124 (2.06) | 46 (5.70) | 29 (1.16) | 40 (1.71) | 9 (2.56) | <.0001 |
| Piperacillin, n (%) | 136 (2.26) | 101 (12.52) | 6 (0.24) | 27 (1.15) | 2 (0.57) | <.0001 |
| **Cephalosporins** | | | | | | |
| Cefazolin, n (%) | 592 (9.85) | 204 (25.28) | 133 (5.31) | 215 (9.17) | 40 (11.36) | <.0001 |
| Cefaclor, n (%) | 110 (1.83) | 61 (7.56) | 11 (0.44) | 30 (1.28) | 8 (2.27) | <.0001 |
| Cefoperazone, n (%) | 59 (0.98) | 49 (6.07) | 1 (0.04) | 7 (0.30) | 2 (0.57) | <.0001 |
| Ceftriaxone, n (%) | 156 (2.60) | 88 (10.90) | 12 (0.48) | 47 (2.00) | 9 (2.56) | <.0001 |
| Cefmetazole, n (%) | 128 (2.13) | 75 (9.29) | 19 (0.76) | 24 (1.02) | 10 (2.84) | <.0001 |
| Cephradine, n (%) | 603 (10.04) | 163 (20.20) | 109 (4.35) | 291 (12.41) | 40 (11.36) | <.0001 |

Suppl 10. The total variables of step 1 (continued)

|  | **Overall (n=6,008)** | **Cluster1 (n=807)** | **Cluster2 (n=2,504)** | **Cluster3 (n=2,345)** | **Cluster4 (n=352)** | ***p-value*** |
| --- | --- | --- | --- | --- | --- | --- |
| Cefixime, n (%) | 183 (3.05) | 90 (11.15) | 21 (0.84) | 58 (2.47) | 14 (3.98) | <.0001 |
| **Beta_lactamase_inh** | | | | | | |
| Sulbactam, n (%) | 111 (1.85) | 66 (8.18) | 13 (0.52) | 24 (1.02) | 8 (2.27) | <.0001 |
| Tazobactam, n (%) | 136 (2.26) | 101 (12.52) | 6 (0.24) | 27 (1.15) | 2 (0.57) | <.0001 |
| Clavulanic acid, n (%) | 264 (4.39) | 93 (11.52) | 51 (2.04) | 100 (4.26) | 20 (5.68) | <.0001 |
| **Aminoglycoside** |  |  |  |  |  |  |
| Gentamicin, n (%) | 75 (1.25) | 31 (3.84) | 14 (0.56) | 25 (1.07) | 5 (1.42) | <.0001 |
| Neomycin, n (%) | 629 (10.47) | 184 (22.80) | 163 (6.51) | 238 (10.15) | 44 (12.50) | <.0001 |
| **Quinolone** |  |  |  |  |  |  |
| Levofloxacin, n (%) | 226 (3.76) | 103 (12.76) | 20 (0.80) | 91 (3.88) | 12 (3.41) | <.0001 |
| Moxifloxacin, n (%) | 154 (2.56) | 65 (8.05) | 27 (1.08) | 53 (2.26) | 9 (2.56) | <.0001 |
| Ciprofloxacin, n (%) | 85 (1.41) | 41 (5.08) | 8 (0.32) | 31 (1.32) | 5 (1.42) | <.0001 |
| **Macrocyclic_antibiotics** | | | | | | |
| Clarithromycin, n (%) | 89 (1.48) | 13 (1.61) | 23 (0.92) | 44 (1.88) | 9 (2.56) | 0.0127 |
| Azithromycin, n (%) | 66 (1.10) | 24 (2.97) | 13 (0.52) | 27 (1.15) | 2 (0.57) | <.0001 |
| Erythromycin, n (%) | 216 (3.60) | 56 (6.94) | 47 (1.88) | 98 (4.18) | 15 (4.26) | <.0001 |
| **Antifungal_drugs** | | | | | | |
| Sertaconazole, n (%) | 112 (1.86) | 29 (3.59) | 25 (1.00) | 48 (2.05) | 10 (2.84) | <.0001 |

Suppl 10. The total variables of step 1 (continued)

|  | **Overall (n=6,008)** | **Cluster1 (n=807)** | **Cluster2 (n=2,504)** | **Cluster3 (n=2,345)** | **Cluster4 (n=352)** | ***p-value*** |
| --- | --- | --- | --- | --- | --- | --- |
| Nystatin, n (%) | 331 (5.51) | 111 (13.75) | 66 (2.64) | 133 (5.67) | 21 (5.97) | <.0001 |
| **Other medications** |  |  |  |  |  |  |
| Flomoxef, n (%) | 150 (2.50) | 75 (9.29) | 27 (1.08) | 43 (1.83) | 5 (1.42) | <.0001 |
| Ertapenem, n (%) | 66 (1.10) | 47 (5.82) | 1 (0.04) | 12 (0.51) | 6 (1.70) | <.0001 |
| Gramicidin, n (%) | 295 (4.91) | 93 (11.52) | 63 (2.52) | 122 (5.20) | 17 (4.83) | <.0001 |
| Clindamycin, n (%) | 84 (1.40) | 17 (2.11) | 31 (1.24) | 29 (1.24) | 7 (1.99) | 0.1921 |
| Doxycycline, n (%) | 64 (1.07) | 15 (1.86) | 20 (0.8) | 24 (1.02) | 5 (1.42) | 0.0729 |
| Fusidic acid, n (%) | 105 (1.75) | 30 (3.72) | 25 (1.00) | 45 (1.92) | 5 (1.42) | <.0001 |
| Sulfamethoxazole, n (%) | 145 (2.41) | 37 (4.58) | 35 (1.40) | 57 (2.43) | 16 (4.55) | <.0001 |
| Metronidazole, n (%) | 94 (1.56) | 34 (4.21) | 18 (0.72) | 34 (1.45) | 8 (2.27) | <.0001 |
| **NSAIDs** | | | | | | |
| Diclofenac, n (%) | 785 (13.07) | 145 (17.97) | 246 (9.82) | 346 (14.75) | 48 (13.64) | <.0001 |
| Ketorolac, n (%) | 311 (5.18) | 79 (9.79) | 92 (3.67) | 118 (5.03) | 22 (6.25) | <.0001 |
| Celecoxib, n (%) | 338 (5.63) | 83 (10.29) | 100 (3.99) | 138 (5.88) | 17 (4.83) | <.0001 |
| Etofenamate, n (%) | 237 (3.94) | 60 (7.43) | 58 (2.32) | 101 (4.31) | 18 (5.11) | <.0001 |
| Mefenamic acid, n (%) | 118 (1.96) | 24 (2.97) | 34 (1.36) | 48 (2.05) | 12 (3.41) | 0.0047 |
| Etoricoxib , n (%) | 190 (3.16) | 49 (6.07) | 36 (1.44) | 93 (3.97) | 12 (3.41) | <.0001 |
| **Anti-gout agents** | | | | | | |

Suppl 10. The total variables of step 1 (continued)

|  | **Overall (n=6,008)** | **Cluster1 (n=807)** | **Cluster2 (n=2,504)** | **Cluster3 (n=2,345)** | **Cluster4 (n=352)** | ***p-value*** |
| --- | --- | --- | --- | --- | --- | --- |
| Febuxostat, n (%) | 182 (3.03) | 59 (7.31) | 45 (1.80) | 70 (2.99) | 8 (2.27) | <.0001 |
| Benzbromarone, n (%) | 207 (3.45) | 26 (3.22) | 86 (3.43) | 80 (3.41) | 15 (4.26) | 0.8411 |
| Colchicine, n (%) | 254 (4.23) | 57 (7.06) | 71 (2.84) | 108 (4.61) | 18 (5.11) | <.0001 |
| Allopurinol , n (%) | 64 (1.07) | 12 (1.49) | 19 (0.76) | 27 (1.15) | 6 (1.70) | 0.1630 |
| **Corticosteroids** | | | | | | |
| Hydrocortisone, n (%) | 319 (5.31) | 142 (17.60) | 54 (2.16) | 102 (4.35) | 21 (5.97) | <.0001 |
| Methylprednisolone, n (%) | 169 (2.81) | 90 (11.15) | 26 (1.04) | 40 (1.71) | 13 (3.69) | <.0001 |
| Fluocinonide, n (%) | 181 (3.01) | 46 (5.70) | 61 (2.44) | 62 (2.64) | 12 (3.41) | <.0001 |
| Dexamethasone, n (%) | 483 (8.04) | 148 (18.34) | 145 (5.79) | 155 (6.61) | 35 (9.94) | <.0001 |
| Betamethasone, n (%) | 229 (3.81) | 73 (9.05) | 57 (2.28) | 84 (3.58) | 15 (4.26) | <.0001 |
| Fluorometholone, n (%) | 118 (1.96) | 24 (2.97) | 27 (1.08) | 56 (2.39) | 11 (3.13) | 0.0003 |
| Prednisolone, n (%) | 416 (6.92) | 122 (15.12) | 106 (4.23) | 162 (6.91) | 26 (7.39) | <.0001 |
| Triamcinolone, n (%) | 357 (5.94) | 102 (12.64) | 83 (3.31) | 151 (6.44) | 21 (5.97) | <.0001 |
| Fluticasone, n (%) | 236 (3.93) | 40 (4.96) | 87 (3.47) | 97 (4.14) | 12 (3.41) | 0.2458 |
| Mometasone, n (%) | 114 (1.90) | 37 (4.58) | 37 (1.48) | 36 (1.54) | 4 (1.14) | <.0001 |
| Clobetasol, n (%) | 66 (1.10) | 13 (1.61) | 19 (0.76) | 32 (1.36) | 2 (0.57) | 0.0703 |
| **Parasympathetic Nerve Suppressants** | | | | | | |
| Ipratropium, n (%) | 100 (1.66) | 61 (7.56) | 13 (0.52) | 22 (0.94) | 4 (1.14) | <.0001 |

Suppl 10. The total variables of step 1 (continued)

|  | **Overall (n=6,008)** | **Cluster1 (n=807)** | **Cluster2 (n=2,504)** | **Cluster3 (n=2,345)** | **Cluster4 (n=352)** | ***p-value*** |
| --- | --- | --- | --- | --- | --- | --- |
| Neostigmine, n (%) | 147 (2.45) | 36 (4.46) | 45 (1.80) | 55 (2.35) | 11 (3.13) | 0.0003 |
| **β2 agonists** | | | | | | |
| Salbutamol , n (%) | 186 (3.10) | 84 (10.41) | 36 (1.44) | 52 (2.22) | 14 (3.98) | <.0001 |
| Vilanterol, n (%) | 84 (1.40) | 20 (2.48) | 30 (1.20) | 30 (1.28) | 4 (1.14) | 0.0466 |
| Procaterol, n (%) | 91 (1.51) | 32 (3.97) | 22 (0.88) | 29 (1.24) | 8 (2.27) | <.0001 |
| **PDE inhibitors** | | | | | | |
| Theophylline , n (%) | 313 (5.21) | 92 (11.40) | 78 (3.12) | 123 (5.25) | 20 (5.68) | <.0001 |
| Aminophylline, n (%) | 64 (1.07) | 21 (2.60) | 17 (0.68) | 25 (1.07) | 1 (0.28) | <.0001 |
| **Sympathomimetic stimulant drugs** | | | | | | |
| Ephedrine, n (%) | 187 (3.11) | 68 (8.43) | 49 (1.96) | 59 (2.52) | 11 (3.13) | <.0001 |
| Methylephedrine, n (%) | 191 (3.18) | 34 (4.21) | 62 (2.48) | 84 (3.58) | 11 (3.13) | 0.0447 |
| **Ergot alkaloid** | | | | | | |
| Nicergoline, n (%) | 258 (4.29) | 62 (7.68) | 60 (2.40) | 119 (5.07) | 17 (4.83) | <.0001 |
| **Antiemetics** | | | | | | |
| Palonosetron, n (%) | 78 (1.30) | 46 (5.70) | 13 (0.52) | 16 (0.68) | 3 (0.85) | <.0001 |
| Prochlorperazine, n (%) | 304 (5.06) | 108 (13.38) | 64 (2.56) | 108 (4.61) | 24 (6.82) | <.0001 |

Suppl 10. The total variables of step 1 (continued)

|  | **Overall (n=6,008)** | **Cluster1 (n=807)** | **Cluster2 (n=2,504)** | **Cluster3 (n=2,345)** | **Cluster4 (n=352)** | ***p-value*** |
| --- | --- | --- | --- | --- | --- | --- |
| **Examination** |  |  |  |  |  |  |
| **Liver** |  |  |  |  |  |  |
| **Liver ECHO** |  |  |  |  |  | <.0001 |
| **Mild, n (%)** | 600 (9.99) | 84 (10.41) | 163 (6.51) | 308 (13.13) | 45 (12.78) | <.0001 |
| **Mild to Moderate, n (%)** | 36 (0.60) | 5 (0.62) | 11 (0.44) | 18 (0.77) | 2 (0.57) | 0.5252 |
| **Moderate, n (%)** | 911 (15.16) | 131 (16.23) | 367 (14.66) | 364 (15.52) | 49 (13.92) | 0.5987 |
| **Moderate to Severe, n (%)** | 126 (2.10) | 9 (1.12) | 54 (2.16) | 53 (2.26) | 10 (2.84) | 0.1657 |
| **Severe, n (%)** | 269 (4.48) | 24 (2.97) | 105 (4.19) | 128 (5.46) | 12 (3.41) | 0.0120 |
| **No test , n (%)** | 4066 (67.68) | 554 (68.65) | 1804 (72.04) | 1474 (62.86) | 234 (66.48) | <.0001 |
| **Complete blood count** |  |  |  |  |  |  |
| **WBC (x103/μL), median (IQR)** | 6.74  (5.69, 8.04) | 7.00  (5.48, 9.04) | 6.81  (5.92, 7.89) | 6.62  (5.53, 7.95) | 6.56  (5.42, 8.22) | <.0001 |
| **RBC (x106/μL), median (IQR)** | 4.72  (4.37, 5.07) | 4.01  (3.57, 4.35) | 4.76  (4.5, 5.06) | 4.77  (4.46, 5.10) | 5.56  (5.00, 6.24) | <.0001 |
| **Hgb (g/dL), median (IQR)** | 14 (13, 15) | 12 (11, 13) | 14 (14, 15) | 14 (14, 15) | 12 (11, 14) | <.0001 |
| **HCT (%), median (IQR)** | 42.0  (39.5, 44.6) | 36.6  (33.1, 39.5) | 42.6  (40.6, 44.9) | 42.9  (40.7, 45.6) | 39.8  (36.2, 43.2) | <.0001 |
| **MCHC (g/dL), median (IQR)** | 33.4  (32.8, 33.9) | 33.2  (32.6, 33.8) | 33.4  (32.9, 33.9) | 33.5  (32.9, 34.1) | 31.3  (30.5, 32.1) | <.0001 |
| **MCH (pg), median (IQR)** | 30 (29, 31) | 30 (29, 32) | 30 (29, 31) | 30 (29, 31) | 22 (21, 23) | <.0001 |
| Suppl 10. The total variables of step 1 (continued) | | | | | | |
|  | **Overall (n=6,008)** | **Cluster1 (n=807)** | **Cluster2 (n=2,504)** | **Cluster3 (n=2,345)** | **Cluster4 (n=352)** | ***p-value*** |
| **MCV (fl), median (IQR)** | 90 (87, 93) | 92 (88, 95) | 90 (87, 92) | 90 (88, 93) | 70 (67, 75) | <.0001 |
| **PLT (x103/μL), median (IQR)** | 236 (202, 272) | 212(173, 258) | 239 (211, 268) | 235 (201, 273) | 254 (214, 320) | <.0001 |
| **RDW-CV (%), median (IQR)** | 13.0  (12.5, 13.7) | 13.4  (12.8, 14.3) | 13.0  (12.5, 13.5) | 12.8  (12.3, 13.3) | 15.8  (14.4, 17.4) | <.0001 |
| **RDW-SD (fl), median (IQR)** | 42.3  (40.9, 43.8) | 43.5  (41.9, 46.0) | 42.3  (41.0, 43.5) | 42.1  (40.6, 43.7) | 41.3  (38.2, 43.0) | <.0001 |
| **White blood cell classification** |  |  |  |  |  |  |
| **NEUT (%), median (IQR)** | 62.76  (58.70, 67.26) | 66.30  (61.26, 72.30) | 62.89  (59.10, 66.83) | 61.76  (57.44, 65.86) | 62.50  (57.05, 66.20) | <.0001 |
| **Monocyte (%), median (IQR)** | 6.10  (5.40, 6.90) | 6.04  (5.00, 7.28) | 6.14  (5.46, 6.80) | 6.10  (5.38, 6.92) | 6.10  (5.32, 6.81) | 0.0079 |
| **Basophil (%), median (IQR)** | 0.48  (0.38, 0.60) | 0.40  (0.20, 0.52) | 0.48  (0.40, 0.58) | 0.50  (0.40, 0.62) | 0.50  (0.40, 0.60) | <.0001 |
| **Segment (%), median (IQR)** | 66.10  (61.12, 71.50) | 69.00  (63.26, 77.00) | 66.01  (61.36, 71.45) | 65.26  (60.46, 70.48) | 65.07  (59.73, 71.37) | <.0001 |
| **Eosinophil (%), median (IQR)** | 2.10  (1.48, 2.90) | 1.70  (0.86, 2.70) | 2.10  (1.56, 2.86) | 2.18  (1.52, 3.00) | 2.10  (1.38, 3.00) | <.0001 |
| **Lymphocyte (%), median (IQR)** | 27.36  (23.04, 31.80) | 21.80  (14.30, 27.60) | 27.26  (23.48, 31.00) | 28.78  (24.60, 33.30) | 28.01  (23.76, 32.18) | <.0001 |
| Suppl 10. The total variables of step 1 (continued) | | | | | | |
|  | **Overall (n=6,008)** | **Cluster1 (n=807)** | **Cluster2 (n=2,504)** | **Cluster3 (n=2,345)** | **Cluster4 (n=352)** | ***p-value*** |
| **Normoblast (%), median (IQR)** | 0.00  (0.00, 0.02) | 0.00  (0.00, 0.02) | 0.00  (0.00, 0.02) | 0.00  (0.00, 0.02) | 0.00  (0.00, 0.04) | <.0001 |
| **Renal profile** |  |  |  |  |  |  |
| **Creatinine (mg/dL), median (IQR)** | 0.86  (0.71, 1.02) | 0.99  (0.78, 1.37) | 0.87  (0.74, 1.00) | 0.84  (0.68, 1.00) | 0.80  (0.65, 0.99) | <.0001 |
| **BUN (mg/dL), median (IQR)** | 14.2  (12.1, 16.9) | 17.3  (13.1, 24.3) | 14.1  (12.2, 16.4) | 13.8  (11.8, 16.2) | 13.6  (11.3, 16.3) | <.0001 |
| **eGFR (mL/min/1.73 m2),  median (IQR)** | 88.7  (74.0, 104.4) | 69.4  (48.5, 88.7) | 90.0  (76.6, 104.4) | 91.4  (77.5, 106.9) | 93.7  (76.6, 111.1) | <.0001 |
| <15, n (%) | 34 (0.57) | 33 (4.09) | 0 (0.00) | 0 (0.00) | 1 (0.28) | <.0001 |
| 15-29, n (%) | 71 (1.18) | 54 (6.69) | 9 (0.36) | 4 (0.17) | 4 (1.14) | <.0001 |
| 30-44, n (%) | 127 (2.11) | 82 (10.16) | 21 (0.84) | 22 (0.94) | 2 (0.57) | <.0001 |
| 45-59, n (%) | 382 (6.36) | 137 (16.98) | 122 (4.87) | 104 (4.43) | 19 (5.40) | <.0001 |
| 60-89, n (%) | 2519 (41.93) | 308 (38.17) | 1097 (43.81) | 983 (41.92) | 131 (37.22) | 0.0091 |
| ≥90, n (%) | 2875 (47.85) | 193 (23.92) | 1255 (50.12) | 1232( 52.54) | 195 (55.40) | <.0001 |
| **Glucose profile** |  |  |  |  |  |  |
| **Glucose level (mg/dL),  median (IQR)** | 109 (99, 126) | 122(103, 155) | 109 (101, 122) | 107 (98, 123) | 106 (97, 126) | <.0001 |

Suppl 10. The total variables of step 1 (continued)

|  | **Overall (n=6,008)** | **Cluster1 (n=807)** | **Cluster2 (n=2,504)** | **Cluster3 (n=2,345)** | **Cluster4 (n=352)** | ***p-value*** |
| --- | --- | --- | --- | --- | --- | --- |
| **HbA1c (%), median (IQR)** | 6.0 (5.7, 6.5) | 6.3 (5.8, 7.0) | 6.0 (5.7, 6.4) | 6.0 (5.7, 6.5) | 6.1 (5.7, 6.6) | <.0001 |
| **Cardiac enzymes** |  |  |  |  |  |  |
| **CK (IU/L), median (IQR)** | 122.2  (95.4, 172.0) | 115.0  (83.4, 169.4) | 121.7  (97.0, 171.0) | 123.8  (95.4, 172.4) | 119.2  (97.2, 171.2) | 0.0001 |
| Troponin-I (ng/mL), median (IQR) | 0.01  (0.01, 0.04) | 0.02  (0.01, 0.05) | 0.01  (0.01, 0.05) | 0.01  (0.01, 0.04) | 0.01  (0.01, 0.02) | <.0001 |
| **Coagulation profile** |  |  |  |  |  |  |
| PTc (second), median (IQR) | 10.82  (10.76, 10.90) | 10.84  (10.74, 10.90) | 10.82  (10.76, 10.90) | 10.82  (10.74, 10.90) | 10.82  (10.76, 10.90) | 0.1133 |
| PTp (second), median (IQR) | 10.40  (10.20, 10.70) | 10.52  (10.20, 11.02) | 10.40  (10.20, 10.70) | 10.40  (10.18, 10.64) | 10.42  (10.20, 10.70) | <.0001 |
| INR, median (IQR) | 1.00  (0.97, 1.03) | 1.01  (0.97, 1.06) | 1.00  (0.97, 1.02) | 0.99  (0.97, 1.02) | 1.00  (0.97, 1.02) | <.0001 |
| PTTc (second), median (IQR) | 26.52  (26.14, 26.82) | 26.56  (26.14, 26.90) | 26.50  (26.14, 26.80) | 26.52  (26.16, 26.84) | 26.52  (26.14, 26.80) | 0.0836 |
| PTTp (second), median (IQR) | 27.60  (26.76, 28.78) | 27.80  (26.58, 29.58) | 27.62  (26.84, 28.70) | 27.56  (26.74, 28.62) | 27.50  (26.50, 28.68) | <.0001 |
| **Lipid profile** |  |  |  |  |  |  |
| HDL-C (mg/dL), median (IQR) | 46 (40, 53) | 45 (38, 53) | 47 (41, 53) | 46 (40, 54) | 45 (39, 53) | <.0001 |

Suppl 10. The total variables of step 1 (continued)

|  | **Overall (n=6,008)** | **Cluster1 (n=807)** | | **Cluster2 (n=2,504)** | | | **Cluster3 (n=2,345)** | | **Cluster4 (n=352)** | | ***p-value*** | |
| --- | --- | --- | --- | --- | --- | --- | --- | --- | --- | --- | --- | --- |
| LDL-C (mg/dL), median (IQR) | 106 (88, 124) | 93 (70, 111) | | 109 (92, 125) | | | 108 (88, 129) | | 101 (88, 120) | | <.0001 | |
| Total cholesterol (mg/dL),  median (IQR) | 182 (160, 205) | 172(145, 192) | | 185 (166, 206) | | | 183 (160, 210) | | 177 (155, 199) | | <.0001 | |
| Triglyceride (mg/dL),  median (IQR) | 124 (90, 167) | 129 (90, 170) | | 128 (95, 169) | | | 118 (85, 164) | | 125 (81, 161) | | <.0001 | |
| **Liver profile** |  | |  | |  |  | |  | |  | |  |
| ALT (IU/L), median (IQR) | 32 (22, 51) | | 24 (18, 39) | | 36 (25, 54) | 32 (22, 51) | | 30 (21, 53) | | <.0001 | |  |
| AST (IU/L), median (IQR) | 30 (24, 39) | | 28 (23, 38) | | 30 (25, 39) | 30 (24, 39) | | 29 (23, 41) | | <.0001 | |  |
| ALK-P (IU/L), median (IQR) | 70 (60, 89) | | 73 (63, 101) | | 70 (59, 89) | 70 (59, 88) | | 70 (60, 85) | | <.0001 | |  |
| GGT(IU/L), median (IQR) | 39 (26, 59) | | 46 (29, 90) | | 39 (26, 58) | 38 (26, 57) | | 37 (26, 58) | | <.0001 | |  |
| Total bilirubin (mg/dL),  median (IQR) | 0.84  (0.70, 1.00) | | 0.80  (0.65, 1.02) | | 0.85  (0.72, 1.01) | 0.84  (0.72, 1.00) | | 0.81  (0.67, 0.97) | | <.0001 | |  |
| Bilirubin Direct (mg/dL),  median (IQR) | 0.17  (0.13, 0.23) | | 0.19  (0.14, 0.26) | | 0.17  (0.13, 0.23) | 0.17  (0.13, 0.22) | | 0.17  (0.13, 0.24) | | <.0001 | |  |
| Albumin (g/dL), median (IQR) | 4.32  (4.10, 4.50) | | 4.11  (3.80, 4.35) | | 4.34  (4.12, 4.49) | 4.37  (4.15, 4.53) | | 4.31  (4.11, 4.49) | | <.0001 | |  |
| **Electrolytes** |  | |  | |  |  | |  | |  | |  |

Suppl 10. The total variables of step 1 (continued)

|  | **Overall (n=6,008)** | **Cluster1 (n=807)** | **Cluster2 (n=2,504)** | **Cluster3 (n=2,345)** | **Cluster4 (n=352)** | ***p-value*** |
| --- | --- | --- | --- | --- | --- | --- |
| Na (mmol/L), median (IQR) | 138.2  (137.0, 139.2) | 138.0  (135.8, 139.2) | 138.2  (137.2, 139.2) | 138.2  (137.0, 139.4) | 138.2  (137.0, 139.2) | <.0001 |
| K (mmol/L), median (IQR) | 3.9 (3.7, 4.0) | 3.9 (3.7, 4.2) | 3.9 (3.7, 4.0) | 3.9 (3.7, 4.0) | 3.9 (3.7, 4.0) | <.0001 |
| Ca2+ (mg/dL), median (IQR) | 4.74  (4.65, 4.83) | 4.71  (4.59, 4.82) | 4.74  (4.65, 4.83) | 4.75  (4.66, 4.83) | 4.73  (4.64, 4.81) | <.0001 |
| **Arterial blood gas analysis** |  |  |  |  |  |  |
| Temperature of blood (℃), median (IQR) | 36.88  (36.68, 37.00) | 36.90  (36.64, 37.00) | 36.86  (36.68, 37.00) | 36.88  (36.7, 37.00) | 36.88  (36.65, 37.01) | 0.6015 |
| pH, median (IQR) | 7.39  (7.37, 7.41) | 7.39  (7.37, 7.42) | 7.39  (7.37, 7.41) | 7.39  (7.37, 7.41) | 7.39  (7.37, 7.41) | 0.0002 |
| pO2 (mmHg), median (IQR) | 56.52  (47.72, 69.30) | 59.70  (46.60, 75.10) | 56.52  (47.88, 69.29) | 55.96  (47.72, 68.16) | 56.54  (47.66, 67.06) | <.0001 |
| pCO2 (mmHg), median (IQR) | 42.15  (39.79, 44.46) | 41.02  (38.20, 43.90) | 42.26  (39.90, 44.52) | 42.32  (39.96, 44.64) | 42.10  (39.80, 44.32) | <.0001 |
| TCO2 (mmol/L), median (IQR) | 27.86  (25.50, 33.86) | 27.20  (24.32, 32.64) | 27.98  (25.60, 34.08) | 27.92  (25.54, 33.92) | 28.10  (25.44, 33.42) | <.0001 |
| BEb (mmol/L), median (IQR) | -0.14  (-1.16, 0.84) | -0.30  (-1.86, 0.90) | -0.14  (-1.16, 0.80) | -0.08  (-1.00, 0.88) | -0.14  (-1.41, 0.80) | <.0001 |

Suppl 10. The total variables of step 1 (continued)

|  | **Overall (n=6,008)** | **Cluster1 (n=807)** | **Cluster2 (n=2,504)** | **Cluster3 (n=2,345)** | **Cluster4 (n=352)** | ***p-value*** |
| --- | --- | --- | --- | --- | --- | --- |
| BEecf (mmol/L), median (IQR) | -0.06  (-1.24, 1.12) | -0.20  (-2.12, 1.12) | -0.06  (-1.18, 1.10) | 0.04  (-1.04, 1.16) | -0.10  (-1.71, 1.10) | <.0001 |
| %sO2c (%), median (IQR) | 79.70  (72.74, 85.96) | 83.52  (74.02, 89.72) | 79.20  (72.74, 85.30) | 78.94  (72.50, 85.38) | 80.56  (72.74, 86.12) | <.0001 |
| **Urine examination** |  |  |  |  |  |  |
| pH, median (IQR) | 6.0 (5.5, 6.2) | 6.0 (5.4, 6.4) | 5.9 (5.7, 6.2) | 6.0 (5.0, 6.5) | 6 (5.5, 6.2) | 0.0020 |
| Specific gravity, median (IQR) | 1.02  (1.01, 1.02) | 1.01  (1.01, 1.02) | 1.02  (1.01, 1.02) | 1.02  (1.01, 1.02) | 1.02  (1.01, 1.02) | <.0001 |
| Urine creatinine (mg/dL),  median (IQR) | 124 (95, 153) | 109 (79, 143) | 127 (101, 152) | 126 (95, 158) | 124 (98, 160) | <.0001 |
| Microalbumin (mg/dL), median (IQR) | 2.32  (1.08, 6.20) | 2.90  (1.18, 7.00) | 2.50  (1.16, 6.49) | 2.00  (0.98, 5.52) | 1.96  (0.92, 5.38) | <.0001 |
| **Color, n (%)** |  |  |  |  |  | <.0001 |
| Colorless | 189 (3.15) | 37 (4.58) | 3 (0.12) | 130 (5.54) | 19 (5.40) | <.0001 |
| Light Yellow | 1328 (22.10) | 296 (36.68) | 10 (0.40) | 936 (39.91) | 86 (24.43) | <.0001 |
| Yellow | 1403 (23.35) | 230 (28.50) | 17 (0.68) | 1060 (45.20) | 96 (27.27) | <.0001 |
| Dark Yellow | 98 (1.63) | 20 (2.48) | 4 (0.16) | 65 (2.77) | 9 (2.56) | <.0001 |
| Light Amber | 15 (0.25) | 2 (0.25) | 0 (0.00) | 11 (0.47) | 2 (0.57) | 0.0008 |

Suppl 10. The total variables of step 1 (continued)

|  | **Overall (n=6,008)** | **Cluster1 (n=807)** | **Cluster2 (n=2,504)** | **Cluster3 (n=2,345)** | **Cluster4 (n=352)** | ***p-value*** |
| --- | --- | --- | --- | --- | --- | --- |
| Amber | 31 (0.52) | 9 (1.12) | 1 (0.04) | 19 (0.81) | 2 (0.57) | <.0001 |
| Dark Amber | 18 (0.30) | 7 (0.87) | 2 (0.08) | 8 (0.34) | 1 (0.28) | 0.0050 |
| Light Orange | 32 (0.53) | 14 (1.73) | 3 (0.12) | 14 (0.60) | 1 (0.28) | <.0001 |
| Orange | 10 (0.17) | 6 (0.74) | 0 (0.00) | 4 (0.17) | 0 (0.00) | 0.0005 |
| Dark Brown | 3 (0.05) | 1 (0.12) | 2 (0.08) | 0 (0.00) | 0 (0.00) | 0.4026 |
| Red | 7 (0.12) | 1 (0.12) | 0 (0.00) | 5 (0.21) | 1 (0.28) | 0.0459 |
| Bloody | 1 (0.02) | 0 (0.00) | 0 (0.00) | 1 (0.04) | 0 (0.00) | 0.5832 |
| Straw | 147 (2.45) | 46 (5.70) | 1 (0.04) | 92 (3.92) | 8 (2.27) | <.0001 |
| No Test | 2726 (45.37) | 138 (17.10) | 2461 (98.28) | 0 (0.00) | 127 (36.08) | <.0001 |
| **Appearance, n (%)** |  |  |  |  |  | <.0001 |
| Clear | 1708 (28.43) | 348 (43.12) | 22 (0.88) | 1219 (51.98) | 119 (33.81) | <.0001 |
| Slightly Cloudy | 157 (2.61) | 49 (6.07) | 8 (0.32) | 82 (3.50) | 18 (5.11) | <.0001 |
| Cloudy | 128 (2.13) | 50 (6.20) | 9 (0.36) | 61 (2.60) | 8 (2.27) | <.0001 |
| Turbid | 15 (0.25) | 9 (1.12) | 2 (0.08) | 3 (0.13) | 1 (0.28) | <.0001 |
| No test | 4000 (66.58) | 351 (43.49) | 2463 (98.36) | 980 (41.79) | 206 (58.52) | <.0001 |
| **Occult blood, n (%)** |  |  |  |  |  | <.0001 |
| - | 2416 (40.21) | 400 (49.57) | 104 (4.15) | 1739 (74.16) | 173 (49.15) | <.0001 |
| +/- | 100 (1.66) | 34 (4.21) | 9 (0.36) | 49 (2.09) | 8 (2.27) | <.0001 |
| 1+ | 580 (9.65) | 147 (18.22) | 26 (1.04) | 373 (15.91) | 34 (9.66) | <.0001 |

Suppl 10. The total variables of step 1 (continued)

|  | **Overall (n=6,008)** | **Cluster1 (n=807)** | **Cluster2 (n=2,504)** | **Cluster3 (n=2,345)** | **Cluster4 (n=352)** | ***p-value*** |
| --- | --- | --- | --- | --- | --- | --- |
| 2+ | 233 (3.88) | 52 (6.44) | 20 (0.80) | 147 (6.27) | 14 (3.98) | <.0001 |
| 3+ | 92 (1.53) | 46 (5.70) | 7 (0.28) | 37 (1.58) | 2 (0.57) | <.0001 |
| No test | 2587 (43.06) | 128 (15.86) | 2338 (93.37) | 0 (0.00) | 121 (34.38) | <.0001 |
| **Red blood cell (/HPF), n (%)** |  |  |  |  |  | <.0001 |
| 0-2 | 2733 (45.49) | 531 (65.80) | 15 (0.60) | 1992 (84.95) | 195 (55.4) | <.0001 |
| 26-50 | 45 (0.75) | 26 (3.22) | 0 (0.00) | 18 (0.77) | 1 (0.28) | <.0001 |
| 51-99 | 21 (0.35) | 10 (1.24) | 2 (0.08) | 9 (0.38) | 0 (0.00) | 0.0001 |
| ≥100 | 64 (1.07) | 25 (3.10) | 4 (0.16) | 33 (1.41) | 2 (0.57) | <.0001 |
| No test | 3145 (52.35) | 215 (26.64) | 2483 (99.16) | 293 (12.49) | 154 (43.75) | <.0001 |
| **White blood cell (/HPF), n (%)** |  |  |  |  |  | <.0001 |
| 0-2 | 2643 (43.99) | 488 (60.47) | 17 (0.68) | 1959 (83.54) | 179 (50.85) | <.0001 |
| 26-50 | 95 (1.58) | 43 (5.33) | 2 (0.08) | 46 (1.96) | 4 (1.14) | <.0001 |
| 51-99 | 70 (1.17) | 33 (4.09) | 1 (0.04) | 30 (1.28) | 6 (1.70) | <.0001 |
| ≥100 | 3 (0.05) | 3 (0.37) | 0 (0.00) | 0 (0.00) | 0 (0.00) | 0.0040 |
| No test | 3197 (53.21) | 240 (29.74) | 2484 (99.2) | 310 (13.22) | 163 (46.31) | <.0001 |
| **Renal epithelial cell (/HPF), n (%)** |  |  |  |  |  | <.0001 |
| 0-2 | 1699 (28.28) | 322 (39.90) | 3 (0.12) | 1268 (54.07) | 106 (30.11) | <.0001 |
| 26-50 | 5 (0.08) | 0 (0.00) | 0 (0.00) | 5 (0.21) | 0 (0.00) | 0.0849 |

Suppl 10. The total variables of step 1 (continued)

|  | **Overall (n=6,008)** | **Cluster1 (n=807)** | **Cluster2 (n=2,504)** | **Cluster3 (n=2,345)** | **Cluster4 (n=352)** | ***p-value*** |
| --- | --- | --- | --- | --- | --- | --- |
| >100 | 1 (0.02) | 0 (0.00) | 0 (0.00) | 0 (0.00) | 1 (0.28) | 0.0586 |
| No test | 4303 (71.62) | 485 (60.10) | 2501 (99.88) | 1072 (45.71) | 245 (69.60) | <.0001 |
| **Glucose level, n (%)** |  |  |  |  |  | <.0001 |
| - | 2908 (48.40) | 512 (63.44) | 149 (5.95) | 2051 (87.46) | 196 (55.68) | <.0001 |
| +/- | 14 (0.23) | 4 (0.50) | 0 (0.00) | 10 (0.43) | 0 (0.00) | 0.0011 |
| 1+ | 83 (1.38) | 34 (4.21) | 5 (0.20) | 37 (1.58) | 7 (1.99) | <.0001 |
| 2+ | 55 (0.92) | 16 (1.98) | 2 (0.08) | 34 (1.45) | 3 (0.85) | <.0001 |
| 3+ | 208 (3.46) | 67 (8.30) | 4 (0.16) | 123 (5.25) | 14 (3.98) | <.0001 |
| 4+ | 102 (1.70) | 28 (3.47) | 2 (0.08) | 64 (2.73) | 8 (2.27) | <.0001 |
| No test | 2638 (43.91) | 146 (18.09) | 2342 (93.53) | 26 (1.11) | 124 (35.23) | <.0001 |
| **Total protein (mg/dL),  median (IQR)** | 15.66  (10.18, 29.36) | 17.60  (10.54, 35.00) | 16.21  (10.52, 30.10) | 14.86  (9.68, 27.90) | 15.20  (10.00, 28.95) | <.0001 |
| **Protein, n (%)** |  |  |  |  |  | <.0001 |
| - | 2741 (45.62) | 451 (55.89) | 124 (4.95) | 1988 (84.78) | 178 (50.57) | <.0001 |
| +/- | 139 (2.31) | 21 (2.60) | 10 (0.40) | 91 (3.88) | 17 (4.83) | <.0001 |
| 1+ | 266 (4.43) | 80 (9.91) | 17 (0.68) | 149 (6.35) | 20 (5.68) | <.0001 |
| 2+ | 140 (2.33) | 71 (8.80) | 6 (0.24) | 53 (2.26) | 10 (2.84) | <.0001 |
| 3+ | 37 (0.62) | 24 (2.97) | 0 (0.00) | 11 (0.47) | 2 (0.57) | <.0001 |
| 4+ | 2 (0.03) | 1 (0.12) | 0 (0.00) | 1 (0.04) | 0 (0.00) | 0.2366 |

Suppl 10. The total variables of step 1 (continued)

|  | **Overall (n=6,008)** | **Cluster1 (n=807)** | **Cluster2 (n=2,504)** | **Cluster3 (n=2,345)** | **Cluster4 (n=352)** | ***p-value*** |
| --- | --- | --- | --- | --- | --- | --- |
| No test | 2683 (44.66) | 159 (19.70) | 2347 (93.73) | 52 (2.22) | 125 (35.51) | <.0001 |
| **Bilirubin, n (%)** |  |  |  |  |  | <.0001 |
| - | 3268 (54.39) | 663 (82.16) | 40 (1.60) | 2342 (99.87) | 223 (63.35) | <.0001 |
| 1+ | 8 (0.13) | 2 (0.25) | 2 (0.08) | 3 (0.13) | 1 (0.28) | 0.3409 |
| 2+ | 4 (0.07) | 3 (0.37) | 1 (0.04) | 0 (0.00) | 0 (0.00) | 0.0210 |
| No test | 2728 (45.41) | 139 (17.22) | 2461 (98.28) | 0 (0.00) | 128 (36.36) | <.0001 |
| **Urobilinogen, n (%)** |  |  |  |  |  | <.0001 |
| Normal | 3104 (51.66) | 633 (78.44) | 31 (1.24) | 2231 (95.14) | 209 (59.38) | <.0001 |
| 1+ | 96 (1.60) | 16 (1.98) | 5 (0.20) | 64 (2.73) | 11 (3.13) | <.0001 |
| 2+ | 33 (0.55) | 12 (1.49) | 1 (0.04) | 20 (0.85) | 0 (0.00) | <.0001 |
| No test | 2775 (46.19) | 146 (18.09) | 2467 (98.52) | 30 (1.28) | 132 (37.50) | <.0001 |
| **Ketone, n (%)** |  |  |  |  |  | <.0001 |
| - | 2960 (49.27) | 590 (73.11) | 26 (1.04) | 2142 (91.34) | 202 (57.39) | <.0001 |
| +/- | 198 (3.30) | 48 (5.95) | 8 (0.32) | 126 (5.37) | 16 (4.55) | <.0001 |
| 1+ | 94 (1.56) | 27 (3.35) | 5 (0.20) | 57 (2.43) | 5 (1.42) | <.0001 |
| 2+ | 25 (0.42) | 4 (0.50) | 2 (0.08) | 18 (0.77) | 1 (0.28) | 0.0009 |
| 3+ | 2 (0.03) | 0 (0.00) | 2 (0.08) | 0 (0.00) | 0 (0.00) | 0.6746 |
| 4+ | 3 (0.05) | 0 (0.00) | 0 (0.00) | 2 (0.09) | 1 (0.28) | 0.1240 |
| No test | 2726 (45.37) | 138 (17.10) | 2461 (98.28) | 0 (0.00) | 127 (36.08) | <.0001 |

Suppl 10. The total variables of step 1 (continued)

|  | **Overall (n=6,008)** | **Cluster1 (n=807)** | **Cluster2 (n=2,504)** | **Cluster3 (n=2,345)** | **Cluster4 (n=352)** | ***p-value*** |
| --- | --- | --- | --- | --- | --- | --- |
| **Ascorbic acid, n (%)** |  |  |  |  |  | <.0001 |
| - | 2003 (33.34) | 405 (50.19) | 13 (0.52) | 1448 (61.75) | 137 (38.92) | <.0001 |
| 1+ | 169 (2.81) | 32 (3.97) | 3 (0.12) | 123 (5.25) | 11 (3.13) | <.0001 |
| 2+ | 258 (4.29) | 48 (5.95) | 3 (0.12) | 196 (8.36) | 11 (3.13) | <.0001 |
| No test | 3578 (59.55) | 322 (39.9) | 2485 (99.24) | 578 (24.65) | 193 (54.83) | <.0001 |
| **Bacteria, n (%)** |  |  |  |  |  | <.0001 |
| - | 1474 (24.53) | 243 (30.11) | 0 (0.00) | 1139 (48.57) | 92 (26.14) | <.0001 |
| +/- | 95 (1.58) | 26 (3.22) | 3 (0.12) | 60 (2.56) | 6 (1.70) | <.0001 |
| 1+ | 94 (1.56) | 41 (5.08) | 3 (0.12) | 47 (2.00) | 3 (0.85) | <.0001 |
| 2+ | 61 (1.02) | 24 (2.97) | 1 (0.04) | 26 (1.11) | 10 (2.84) | <.0001 |
| 3+ | 44 (0.73) | 21 (2.60) | 1 (0.04) | 21 (0.90) | 1 (0.28) | <.0001 |
| 4+ | 39 (0.65) | 22 (2.73) | 0 (0.00) | 14 (0.60) | 3 (0.85) | <.0001 |
| No test | 4201 (69.92) | 430 (53.28) | 2496 (99.68) | 1038 (44.26) | 237 (67.33) | <.0001 |
| **Leukocyte, n (%)** |  |  |  |  |  | <.0001 |
| - | 1621 (26.98) | 274 (33.95) | 11 (0.44) | 1236 (52.71) | 100 (28.41) | <.0001 |
| +/- | 168 (2.80) | 34 (4.21) | 0 (0.00) | 117 (4.99) | 17 (4.83) | <.0001 |
| 1+ | 151 (2.51) | 32 (3.97) | 3 (0.12) | 104 (4.43) | 12 (3.41) | <.0001 |
| 2+ | 115 (1.91) | 27 (3.35) | 2 (0.08) | 79 (3.37) | 7 (1.99) | <.0001 |
| 3+ | 131 (2.18) | 48 (5.95) | 4 (0.16) | 70 (2.99) | 9 (2.56) | <.0001 |

Suppl 10. The total variables of step 1 (continued)

|  | **Overall (n=6,008)** | **Cluster1 (n=807)** | **Cluster2 (n=2,504)** | **Cluster3 (n=2,345)** | **Cluster4 (n=352)** | ***p-value*** |
| --- | --- | --- | --- | --- | --- | --- |
| No test | 3822 (63.62) | 392 (48.57) | 2484 (99.2) | 739 (31.51) | 207 (58.81) | <.0001 |
| **Nitrite, n (%)** |  |  |  |  |  | <.0001 |
| - | 3178 (52.90) | 614 (76.08) | 41 (1.64) | 2307 (98.38) | 216 (61.36) | <.0001 |
| + | 73 (1.22) | 38 (4.71) | 2 (0.08) | 27 (1.15) | 6 (1.70) | <.0001 |
| No test | 2757 (45.89) | 155 (19.21) | 2461 (98.28) | 11 (0.47) | 130 (36.93) | <.0001 |
| **Squamous epithelial cell (/HPF), n (%)** |  |  |  |  |  | <.0001 |
| 0-2 | 1461 (24.32) | 361 (44.73) | 29 (1.16) | 975 (41.58) | 96 (27.27) | <.0001 |
| 26-50 | 7 (0.12) | 1 (0.12) | 0 (0.00) | 5 (0.21) | 1 (0.28) | 0.0459 |
| 51-99 | 1 (0.02) | 0 (0.00) | 0 (0.00) | 1 (0.04) | 0 (0.00) | 0.5832 |
| No test | 4539 (75.55) | 445 (55.14) | 2475 (98.84) | 1364 (58.17) | 255 (72.44) | <.0001 |
| **Stool examination** |  |  |  |  |  |  |
| **Occult blood , n (%)** |  |  |  |  |  | <.0001 |
| - | 804 (13.38) | 179 (22.18) | 39 (1.56) | 526 (22.43) | 60 (17.05) | <.0001 |
| +/- | 12 (0.20) | 5 (0.62) | 1 (0.04) | 3 (0.13) | 3 (0.85) | 0.0007 |
| 1+ | 75 (1.25) | 23 (2.85) | 4 (0.16) | 38 (1.62) | 10 (2.84) | <.0001 |
| 2+ | 54 (0.90) | 24 (2.97) | 2 (0.08) | 25 (1.07) | 3 (0.85) | <.0001 |
| 3+ | 46 (0.77) | 14 (1.73) | 4 (0.16) | 22 (0.94) | 6 (1.70) | <.0001 |
| 4+ | 26 (0.43) | 15 (1.86) | 1 (0.04) | 8 (0.34) | 2 (0.57) | <.0001 |
| No test | 4991 (83.07) | 547 (67.78) | 2453 (97.96) | 1723 (73.48) | 268 (76.14) | <.0001 |

Suppl 10. The total variables of step 1 (continued)

|  | **Overall (n=6,008)** | **Cluster1 (n=807)** | **Cluster2 (n=2,504)** | **Cluster3 (n=2,345)** | **Cluster4 (n=352)** | ***p-value*** |
| --- | --- | --- | --- | --- | --- | --- |
| **Blood , n (%)** |  |  |  |  |  | <.0001 |
| Negative | 758 (12.62) | 172 (21.31) | 30 (1.20) | 500 (21.32) | 56 (15.91) | <.0001 |
| No test | 5250 (87.38) | 635 (78.69) | 2474 (98.80) | 1845 (78.68) | 296 (84.09) | <.0001 |
| **Charact, n (%)** |  |  |  |  |  | <.0001 |
| Fluid | 8 (0.13) | 2 (0.25) | 0 (0.00) | 4 (0.17) | 2 (0.57) | 0.0089 |
| Semi-Fluid | 7 (0.12) | 2 (0.25) | 1 (0.04) | 4 (0.17) | 0 (0.00) | 0.2715 |
| Soft | 155 (2.58) | 46 (5.70) | 2 (0.08) | 98 (4.18) | 9 (2.56) | <.0001 |
| Formed | 582 (9.69) | 121 (14.99) | 26 (1.04) | 390 (16.63) | 45 (12.78) | <.0001 |
| Hard | 6 (0.10) | 1 (0.12) | 1 (0.04) | 4 (0.17) | 0 (0.00) | 0.5218 |
| No test | 5250 (87.38) | 635 (78.69) | 2474 (98.80) | 1845 (78.68) | 296 (84.09) | <.0001 |
| **Color, n (%)** |  |  |  |  |  | <.0001 |
| Black | 5 (0.08) | 4 (0.50) | 1 (0.04) | 0 (0.00) | 0 (0.00) | 0.0034 |
| Dark Brown | 5 (0.08) | 3 (0.37) | 0 (0.00) | 2 (0.09) | 0 (0.00) | 0.0198 |
| Brown | 793 (13.20) | 182 (22.55) | 31 (1.24) | 526 (22.43) | 54 (15.34) | <.0001 |
| Yellow | 35 (0.58) | 6 (0.74) | 1 (0.04) | 22 (0.94) | 6 (1.70) | <.0001 |
| Yellow Green | 1 (0.02) | 1 (0.12) | 0 (0.00) | 0 (0.00) | 0 (0.00) | 0.1929 |
| Green | 4 (0.07) | 3 (0.37) | 0 (0.00) | 0 (0.00) | 1 (0.28) | 0.0020 |
| Red | 1 (0.02) | 1 (0.12) | 0 (0.00) | 0 (0.00) | 0 (0.00) | 0.1929 |
| White | 1 (0.02) | 1 (0.12) | 0 (0.00) | 0 (0.00) | 0 (0.00) | 0.1929 |

Suppl 10. The total variables of step 1 (continued)

|  | **Overall (n=6,008)** | **Cluster1 (n=807)** | **Cluster2 (n=2,504)** | **Cluster3 (n=2,345)** | **Cluster4 (n=352)** | ***p-value*** |
| --- | --- | --- | --- | --- | --- | --- |
| No test | 5163 (85.94) | 606 (75.09) | 2471 (98.68) | 1795 (76.55) | 291 (82.67) | <.0001 |
| **Fat, n(%)** |  |  |  |  |  | <.0001 |
| Negative | 758 (12.62) | 172 (21.31) | 30 (1.20) | 500 (21.32) | 56 (15.91) | <.0001 |
| No test | 5250 (87.38) | 635 (78.69) | 2474 (98.80) | 1845 (78.68) | 296 (84.09) | <.0001 |
| **Gas bub, n(%)** |  |  |  |  |  | <.0001 |
| Negative | 756 (12.58) | 172 (21.31) | 30 (1.20) | 498 (21.24) | 56 (15.91) | <.0001 |
| Positive | 2 (0.03) | 0 (0.00) | 0 (0.00) | 2 (0.09) | 0 (0.00) | 0.5009 |
| No test | 5250 (87.38) | 635 (78.69) | 2474 (98.80) | 1845 (78.68) | 296 (84.09) | <.0001 |
| **Mucus, n (%)** |  |  |  |  |  | <.0001 |
| Negative | 755 (12.57) | 172 (21.31) | 29 (1.16) | 498 (21.24) | 56 (15.91) | <.0001 |
| Positive | 3 (0.05) | 0 (0.00) | 1 (0.04) | 2 (0.09) | 0 (0.00) | 0.7966 |
| No test | 5250 (87.38) | 635 (78.69) | 2474 (98.8) | 1845 (78.68) | 296 (84.09) | <.0001 |
| **Ova, n (%)** |  |  |  |  |  | <.0001 |
| Negative | 793 (13.20) | 180 (22.30) | 31 (1.24) | 525 (22.39) | 57 (16.19) | <.0001 |
| No test | 5215 (86.8) | 627 (77.70) | 2473 (98.76) | 1820 (77.61) | 295 (83.81) | <.0001 |
| **Pus cell, n (%)** |  |  |  |  |  | <.0001 |
| Negative | 768 (12.78) | 177 (21.93) | 31 (1.24) | 503 (21.45) | 57 (16.19) | <.0001 |
| No test | 5240 (87.22) | 630 (78.07) | 2473 (98.76) | 1842 (78.55) | 295 (83.81) | <.0001 |
| **Others** |  |  |  |  |  |  |

Suppl 10. The total variables of step 1 (continued)

|  | | **Overall (n=6,008)** | | **Cluster1 (n=807)** | | **Cluster2 (n=2,504)** | | **Cluster3 (n=2,345)** | **Cluster4 (n=352)** | ***p-value*** |
| --- | --- | --- | --- | --- | --- | --- | --- | --- | --- | --- |
| **HBsAg** |  | |  | |  | |  | | | 0.0497 |
| Reactive | | 271 (4.51) | | 30 (3.72) | | 105 (4.19) | | 116 (4.95) | 20 (5.68) | 0.2702 |
| Nonreactive | | 1721 (28.65) | | 267 (33.09) | | 695 (27.76) | | 657 (28.02) | 102 (28.98) | 0.0264 |
| No test | | 4016 (66.84) | | 510 (63.20) | | 1704 (68.05) | | 1572 (67.04) | 230 (65.34) | 0.0756 |
| **HCV IGG** |  | |  | |  | |  | | | <.0001 |
| Reactive | | 75 (1.25) | | 14 (1.73) | | 14 (0.56) | | 42 (1.79) | 5 (1.42) | 0.0007 |
| Nonreactive | | 1551 (25.82) | | 247 (30.61) | | 617 (24.64) | | 585 (24.95) | 102 (28.98) | 0.0026 |
| No test | | 4382 (72.94) | | 546 (67.66) | | 1873 (74.80) | | 1718 (73.26) | 245 (69.60) | 0.0005 |
| **Alpha-fetoprotein(AFP),  median (IQR)** | | 3.30  (2.69, 4.07) | | 3.34  (2.71, 4.10) | | 3.30  (2.70, 4.04) | | 3.29  (2.66, 4.05) | 3.29  (2.68, 4.15) | 0.1694 |
| **CEA (ng/mL), median (IQR)** | | 2.0  (1.6, 2.6) | | 2.0  (1.6, 2.6) | | 2.0  (1.6, 2.6) | | 2.0  (1.5, 2.6) | 2.0  (1.6, 2.5) | 0.0335 |
| **Uric Acid (mg/dL), median (IQR)** | | 6.06  (5.36, 6.72) | | 6.00  (5.24, 6.82) | | 6.10  (5.50, 6.70) | | 6.04  (5.30, 6.80) | 6.02  (5.20, 6.65) | 0.1250 |
| **C-reactive protein (mg/L),  median (IQR)** | | 7.78  (2.98, 18.86) | | 10.64  (3.08, 33.50) | | 8.72  (3.61, 19.67) | | 5.78  (2.40, 15.10) | 7.34  (2.47, 16.67) | <.0001 |
| **Lipase (U/L), median (IQR)** | | 38.4  (33.0, 45.4) | | 38.2  (32.4, 45.6) | | 38.6  (33.2, 45.6) | | 38.2  (32.8, 44.8) | 39.2  (34.0, 46.6) | 0.1538 |

| Suppl 10. The total variables of step 1 (continued) | | | | | | |
| --- | --- | --- | --- | --- | --- | --- |
|  | **Overall (n=6,008)** | **Cluster1 (n=807)** | **Cluster2 (n=2,504)** | **Cluster3 (n=2,345)** | **Cluster4 (n=352)** | ***p-value*** |
| **Free thyroxine (ng/dL), median (IQR)** | 0.91  (0.86, 0.97) | 0.92  (0.87, 0.99) | 0.91  (0.85, 0.97) | 0.91  (0.86, 0.97) | 0.91  (0.85, 0.96) | 0.0002 |
| **TSH (blood) (μIU/mL), median (IQR)** | 1.86  (1.41, 2.41) | 1.87  (1.40, 2.49) | 1.89  (1.45, 2.42) | 1.82  (1.37, 2.36) | 1.92  (1.46, 2.39) | 0.0249 |
| **CA-199 (U/mL), median (IQR)** | 17.70  (13.40, 24.95) | 19.36  (14.40, 29.08) | 17.70  (13.63, 24.80) | 17.26  (13.10, 24.28) | 17.86  (13.68, 24.80) | <.0001 |
